# The Science of Design: Introducing Design of Experiments (DoE) through a Hydrogel-Based High School Chemistry Investigation

**DOI:** 10.1021/acs.jchemed.6c00856

**Published:** 2026-09-09

**Authors:** Penelope E. Jankoski, Jamie Sorrell, Harrison A. Livingston, Dori C. Harcharik, Claire M. Clemons, Tristan D. Clemons

**Affiliations:** † School of Polymer Science and Engineering, 5104 University of Southern Mississippi, Hattiesburg, Mississippi 39406, United States; ‡ Sumrall High School, Sumrall, Mississippi 39402, United States

**Keywords:** Hydrogels, lab-based learning, Design of Experiments (DoE), Materials Science, High School Science

## Abstract

Creating lessons that connect classroom content to real-world application can enhance high school students’ interest in STEM curricula. The scientific method is one such component of the curriculum that can be meaningfully connected to techniques and practices used across a range of STEM fields. Specifically, Design of Experiments (DoE) is a statistical process widely used in STEM industries and research and development contexts to efficiently collect and analyze scientific data, yet it is rarely taught at the high school level. Importantly, DoE also introduces foundational concepts in structured data generation, multivariable analysis, and pattern recognition that underpin emerging artificial intelligence (AI) and machine learning (ML) approaches. To bridge this gap between methodological understanding and real-world application, this study implemented a hands-on DoE-based lesson focused on polymer hydrogels, which was assessed in a high school science classroom with students across grades 9–12. Pre- and postlesson assessments, consisting of multiple-choice, short-answer, and conceptual understanding questions, were used to evaluate shifts in students’ content knowledge and understanding of the DoE approach. The median correct response rate for the conceptual understanding section increased from 33.33% to 66.67% between the pre- and postassessments. Additionally, while response rates for conceptual questions increased following the intervention, a statistically significant improvement in response accuracy was not observed. This pilot study serves as a valuable starting point for designing STEM lessons that introduce students to DoE as a framework for experimental design, while also providing insight into student understanding of this approach—an essential skill set for preparing students for future STEM workforce demands.

## Graphical abstract

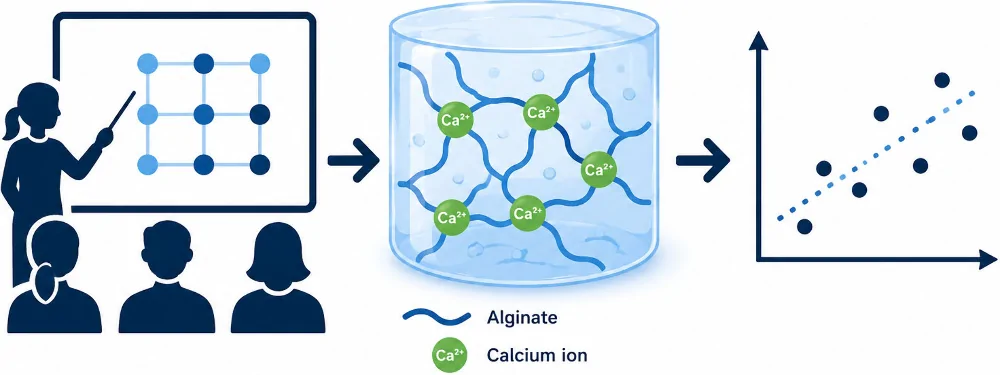

## Introduction

Studies have illustrated that pedagogies which provide exploratory opportunities for students to experience science, technology, engineering and mathematics (STEM) lessons that facilitate classroom-to-world connections are associated with sustained interest in STEM careers in high school students.^1^ Considering this evidence, there’s value in developing classroom activities that offer experiential exposure to relevant concepts and tools utilized within industry in STEM related fields. The scientific method and experimental design are two processes outlined in the nationally recognized Next Generation Science Standards (MS-ETS1 Engineering Design Next Generation Science Standards).^2^ Hence, curricula that present methodologies for collecting and analyzing scientific data provide an opportunity for students to explore industrially relevant tools that drive scientific inquiry, while for teachers still meeting national standard requirements.^3^

Design of experiments (DoE) is one such valuable experimental tool, highlighting which independent variable(s) have a statistically significant influence on experimental outcomes.^4^ Under the DoE methodology, multiple variables can be altered in tandem, a technique that differs from the scientific process traditionally taught in K-12 classrooms, which assesses an outcome as a result from one change in an independent variable at a time.^5^ While one-factor-at-a-time designs might be valuable for young students as an introduction to experimental processes, DoE methods are more relevant and useful, as they save time and resources and are implemented heavily across STEM industries for complex multivariate research problems.^6^ The DoE framework is ultimately a process optimization approach that can be applied to a myriad of projects, from assessing what experimental conditions lead to the best yields in a chemical reaction to adjusting different settings on a machine to assess their impact on part quality. Educating students about the importance of DoE presents additional opportunities to connect classroom content to tools that are industrially implemented, providing a well-rounded picture of how STEM industries optimize systems, performance, and resource use when they face complex scientific problems. In reality, optimization frameworks, such as DoE, are standard compared to the one-factor-at-a-time style of variable assessment taught in K-12 STEM classrooms, underscoring the importance of exposure to the DoE methodology and providing students with a deeper understanding of statistical tools they might actually use in the STEM workforce. Further, as a statistical method, DoE provides an effective gateway to artificial intelligence (AI) and machine learning (ML) in the high school classroom by emphasizing systematic data collection, multivariate relationships, and trend recognition.^7^ Although AI and ML themselves are not explicitly covered in this activity, the concepts introduced through DoE underpin modern approaches to analyzing large, complex data sets and are critical for developing future STEM workforce competencies. Given the importance of DoE strategies in STEM fields and the impact of relevant experiential learning opportunities on student outcomes, this study presents a lesson plan to introduce DoE concepts to students by building on previously developed experimental skills, taking into consideration several prior works as inspiration for the development of this project.^5,8,9^

Considering this intervention’s application in a high school biology classroom, there was motivation to facilitate connections between the DoE framework and curriculum-relevant content in the development of a hands-on experimental lesson plan suitable for high-school STEM classrooms. Hydrogels are soft, flexible, and porous polymers that can absorb and retain fluids up to 1000 times their original weight. Synthetic hydrogel polymers can mimic the permeability of physiological membranes, making them useful in tissue engineering, 3D-bioprinting, and drug delivery materials for medical applications.^10–12^ Such applications of hydrogels position the discussion of this topic as a relevant opportunity for connection to Biology I and AP Biology standard concepts of membranes and permeability, specifically Mississippi high school standards under *BIO 1. Cells as a System*.^12,13^ This standard outlines concepts that include membrane permeability, diffusion, homeostasis, and active transport.^13^ Through our laboratory lesson, students explored how to leverage diffusion for drug delivery using hydrogel materials while contributing to a class-wide full factorial DoE with nine different hydrogel compositions created. The lesson allowed students to draw conclusions about which factor had the biggest influence on the hydrogel properties and suggest an optimal hydrogel design given the data collected across the nine different samples. Importantly, the concept of a DoE approach to assessing experimental variables was introduced and conducted by the students to see the potential of this important industry relevant methodology for experimental design. Pre- and postsurveys validated by the Experimental Design Ability Test (EDAT) allowed for assessment of student knowledge gained as a result of this lesson.^14^ Ultimately, this lesson met two needs: it provided an opportunity for experiential learning about a polymer material valuable to the field of biology while introducing high school students to the DoE approach as a relevant data collection and analysis method commonly used in the STEM workforce.

## Experimental Section

### Materials and Consumables

The materials and consumables necessary as shown in Figure 1 are listed below:

- Sodium Alginate
- Calcium Chloride
- Sodium Chloride
- Deionized or Distilled Water
- Food Dye (Optional – Not Shown)
- Spatula
- Stir Rod
- Petri Dishes
- Beakers
- Scale (0.01 g Sensitivity)
- Ramp (Makeshift Using Books with a Disposable Slick Surface)
- Timer
- Ruler
- Weigh Boats
- Syringes

**Figure 1:**
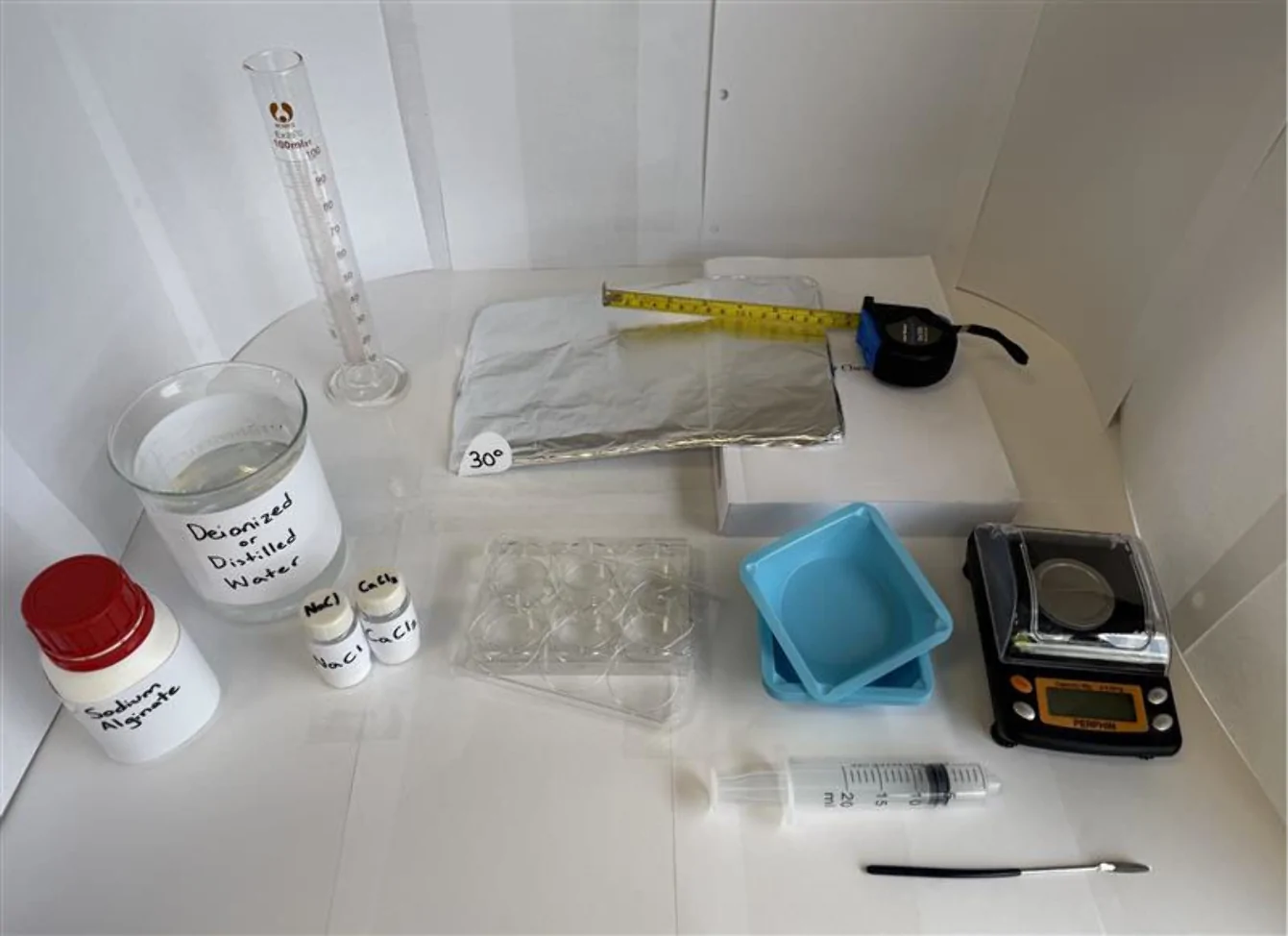
Experimental materials and consumables required for ionically cross-linked sodium alginate preparation and characterization.

Sodium alginate and calcium chloride were obtained from Flinn Scientific, and all other reagents and materials were obtained from Amazon and used as received. The materials used in this activity present minimal safety risks and are appropriate for a high-school laboratory setting. A detailed experimental guide for teachers interested in implementing this experiment in their own classroom is available in the Supporting Information, including learning objectives, curriculum integration, student lab assignments, and student worksheets (see Supporting Information Appendix 1). A visual overview of the key aspects of the experimental procedure is provided in Figure 2.

**Figure 2:**
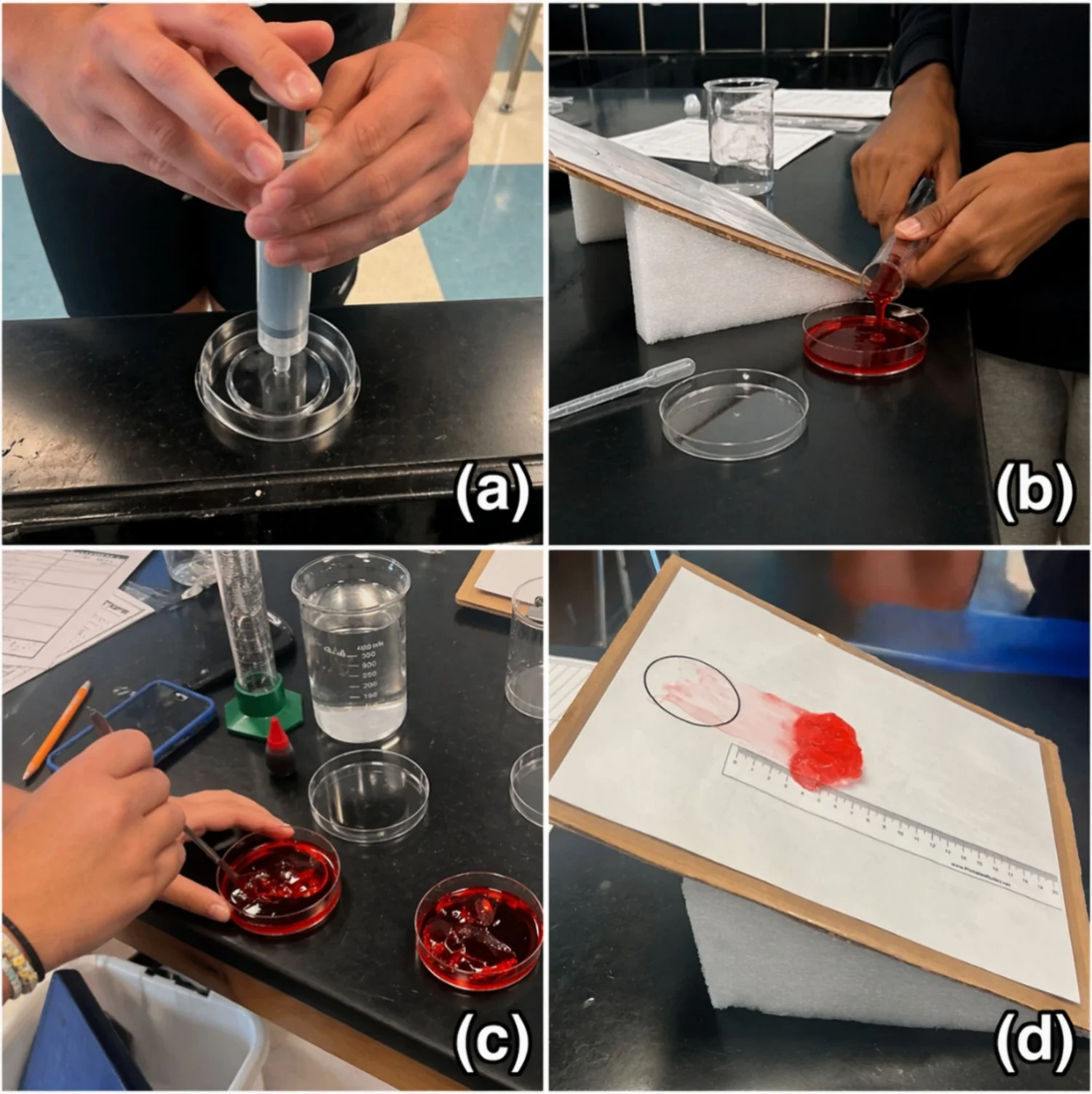
(a) Student extruding appropriate amount of sodium alginate solution onto Petri dish. (b) Student adding appropriate amount of calcium chloride solution with food dye to sodium alginate solution. (c) Student mixing to form ionic cross-link between Ca^2+^ ions and sodium alginate. (d) One of the two characterization methods demonstrated: mechanical assay of hydrogel to assess hydrogel mobility.

### Gel Cross-Linking

Alginate will ionically cross-link following exposure to divalent cations, such as the calcium­(II) ions in CaCl_2_, to produce a hydrogel, where hydrogel stiffness can be tuned by varying the concentration of alginate and calcium ions in the mixture. For the full factorial DoE (2 factor, 3 level), students were assigned to groups of 4 with each group having its own unique combination of alginate and calcium concentration. This resulted in a total of 9 specific formulations across the class (low + low, low + middle, low + high, etc.), as shown in Table 1. Following completion of the lab, students collated data from their classmates to have the full data set, allowing a DoE approach to be performed in only one class period. This lesson was implemented on three occasions, allowing for data to be collected in triplicate across each formulation to be shared among students, creating a complete and statistically robust data set for the DoE. Prior to student arrival, the sodium alginate was dissolved in water at 3 different concentrations, and the calcium chloride was prepared in water at 3 different concentrations (Table 1), to ensure best utilization of class time on student measurement and observations. To produce hydrogels, students were instructed to obtain 10 mL of alginate in their Petri dish (Figure 2a), and 10 mL of calcium chloride solution in a graduated cylinder (Figure 2b) at the desired concentrations for their DoE conditions. Students then carefully poured the cross-linking solution onto the alginate and mixed vigorously (Figure 2c), gels were allowed to set for 5 min, and excess cross-linking solution was drained before further analysis.

**Table 1:** DoE Factors and Levels for Hydrogel Formulation

	Level		
Factor	Low	Middle	High
Alginate Concentration (wt %)	1	1.5	2
Calcium Concentration (M)	0.02	0.05	0.1

### Hydrogel Ion-Exchange Observations

To simulate drug-delivery behavior and the ionic exchange processes that occur in physiological environments, the alginate hydrogels were incubated in a high-concentration sodium chloride solution. In this environment, diffusion of sodium ions into the gel and the corresponding diffusion of calcium ions out of the gel drive an ion-exchange process in which sodium competitively displaces calcium within the alginate network. As cross-links are gradually weakened or replaced, the hydrogel begins to relax, swell, or partially dissolve. This process models how hydrogels release encapsulated agents in the body by responding to local ionic conditions. These hydrogel changes arise directly from diffusion gradients, osmotic pressure differences, and relative ion concentration concepts central to high-school science standards. The measurement of this process provides a tangible, observable example of how molecular movement across concentration gradients governs macroscopic material behavior. Hydrogels were submersed in this concentrated solution for 5 min, analyzed, and then returned to the sodium chloride solution at 5 min increments until class time elapsed to observe changes over time.

### Material Performance Monitoring

To characterize the hydrogels and monitor changes resulting from ionic diffusion in a manner that is accessible and cost-effective, two complementary measurement approaches were implemented. First, the mass of each hydrogel was recorded over time using a standard laboratory balance and a Petri dish, allowing students to quantify changes in water content or structural swelling resulting from incubation in high-concentration sodium chloride solutions. Second, a simple, low-cost pseudomechanical assay was developed to assess the impact of these changes on hydrogel mobility. In this assay, a cardboard ramp was lined with laminated paper to reduce friction, and hydrogels were placed at the top of the ramp while the time required for each to slide to the bottom was recorded. Although this approach does not directly measure mechanical properties such as elastic modulus or viscosity, it provides a simple way to compare changes in hydrogel mobility as swelling and degradation progress over time. As the hydrogels swell, changes in their structural integrity and interactions with the ramp surface contribute to differences in the slide time. Because the angle of the ramp and the surface material can influence these measurements, the same ramp geometry and materials should be used throughout the activity to ensure consistent comparisons among student groups. Collectively, these methods offered students an economical and hands-on approach to investigating hydrogel behavior and the effects of ionic interactions over time as a function of the larger DoE operating within the class setting.

### Safety Hazards

This activity uses materials with minimal hazards, but standard laboratory precautions should still be followed. Students handle sodium alginate, dilute aqueous calcium chloride, and food-grade dyes, all of which are considered low risk when used at the concentrations described. Sodium alginate and food dye may cause minor eye or skin irritation; contact should be avoided, and any spills should be rinsed with water. Dilute calcium chloride solutions can also cause mild irritation; therefore, students should wear safety goggles and appropriate lab attire. The use of disposable gloves may be recommended or required in accordance with institutional policies or the instructor’s discretion. All solutions were clearly labeled. Surfaces should be cleaned after the activity, and all materials can be disposed of in regular municipal waste streams.

## Results and Discussion

### Factorial DoE

Full factorial design of experiments provides an extension of the scientific method and engineering design process, both of which are emphasized in high school science curricula. In contrast to traditional single-variable experiments, which isolate one independent variable at a time, factorial designs allow students to explore the combined effects of multiple variables on an outcome. For example, in a hypothesis-driven slime lab performed the week prior to implementation, students are traditionally instructed to vary only one component, such as the amount of borax or glue, while keeping all other factors constant to determine its effect on the slime’s material properties. However, students often express a desire to adjust multiple variables simultaneously to see “what happens,” reflecting curiosity and an intuitive understanding of system complexity. Introducing a full factorial approach enables students to systematically investigate interactions between variables, quantify their relative contributions, and make data-driven conclusions, thereby reinforcing critical thinking, experimental planning, and the iterative nature of engineering design.

### Mechanisms of Cross-linking

Alginate is a linear polysaccharide composed of β-D-mannuronic acid (M) and α-L-guluronic acid (G) residues, each containing carboxylate groups capable of coordinating multivalent cations (Figure 3).^15^ These residues naturally occur in three distinct arrangements: MG, MM, and GG blocks.^15^ Introduction of Ca^2+^ ions promote the formation of “egg-box” junction zones, as shown in the GG block, in which calcium ions form coordination cross-links with carboxylate groups on neighboring alginate chains, producing a three-dimensional polymer network (Figure 3).^16^ The different block arrangements promote varying degrees of ionic cross-linking, with GG-rich regions forming the characteristic “egg-box” junctions that produce the highest modulus, whereas MM-rich regions exhibit the lowest modulus because they coordinate calcium less effectively. Unlike covalent reactions such as transesterification, this gelation process occurs spontaneously under neutral pH and aqueous conditions. Electrostatic attraction brings Ca^2+^ ions into proximity with the negatively charged alginate chains, while specific ionic coordination between calcium ions and carboxylate groups forms the cross-links responsible for gelation. The extent of cross-linking depends on the local concentration of calcium ions. Low calcium levels produce only partial coordination and yield softer, more flexible hydrogels, while higher concentrations generate more junction zones and increasingly rigid hydrogels until the system nears saturation of available binding sites.^15^ These differences in cross-link density directly shaped the measurable outcomes observed by students. Hydrogels formed under lower-calcium conditions exhibited greater swelling, larger mass changes over time, and slower descent in the pseudomechanical ramp assay, consistent with a less cohesive network. In contrast, highly cross-linked gels demonstrated minimal swelling, retained mass more effectively, and traveled faster down the ramp. Together, these results demonstrate how controlling calcium ion concentration enables predictable tuning of alginate hydrogel structure and properties, supporting accessible classroom exploration of ionic bonding, diffusion, and structure–property relationships.

**Figure 3:**
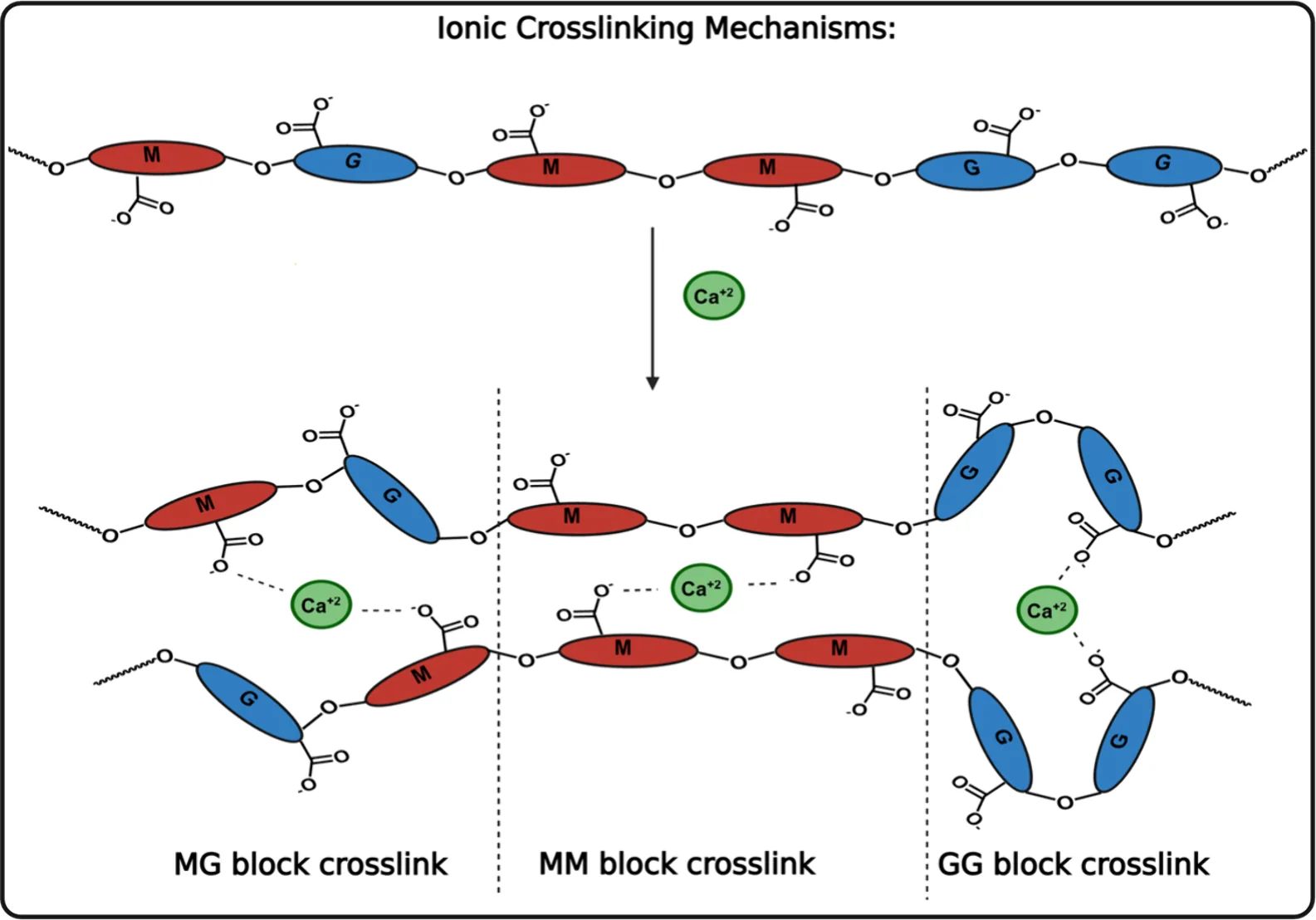
Ionic cross-linking mechanism of alginate-calcium system composed of β-D-mannuronic acid (M) and α-L-guluronic acid (G) residues.^15^

Students at the high school level typically have a foundational understanding of atoms, elements, and basic molecular structure. This prior knowledge allows for the introduction of ionic cross-linking by building on familiar concepts: functional groups can be presented as “puzzle pieces” with specific charges, where only complementary charges interact. For example, in alginate–calcium hydrogels, the negatively charged carboxylate groups of alginate chains interact with positively charged calcium ions to form a stable network. Framing the process this way leverages students’ existing understanding of molecular interactions and allows them to appreciate how ionic forces can drive macroscopic properties such as gelation. Furthermore, connecting this to familiar reaction principles, such as catalysis and reaction conditions, reinforces that reactions and interactions can be controlled under mild conditions when the appropriate components and forces are present.

### Monitoring Hydrogel Degradation

Incubation of the hydrogels in a concentrated sodium chloride solution, results in the displacement of divalent calcium ionic cross-links and observable changes in the structural integrity of the hydrogel. High school students are generally familiar with everyday concepts of force, stretch, and resistance, even if they have not formally studied material mechanics. This prior understanding can be leveraged to introduce mechanical characterization of hydrogels. For example, students can relate the stiffness or elasticity of an alginate–calcium hydrogel to how tightly the polymer chains are cross-linked by calcium ions. By drawing this connection, students can see that molecular interactions such as ionic cross-links have tangible effects on macroscopic properties like firmness, stretchability, and resilience. This approach allows students to connect molecular structure to observable mechanical behavior, reinforcing the idea that chemistry at the molecular scale governs macroscale material properties.

### Bridging Polymer Science to Biology

Although alginate–calcium hydrogels are structurally different from biological lipid bilayers, they provide a useful model for introducing transport phenomena commonly encountered in biology. High school students are generally familiar with the movement of substances into and out of cells through processes such as diffusion, osmosis, and permeability. This activity builds on that prior knowledge by allowing students to observe how the properties of a hydrogel change as the density of ionic cross-links within the polymer network changes. By measuring swelling and degradation, students can relate changes in hydrogel structure to the transport behavior of the material and discuss how these changes influence the movement of water and other molecules through hydrated polymer networks. As water enters the hydrogel, the polymer network swells because of the osmotic driving force generated by the ionically cross-linked alginate network.^15^ Unlike biological cells, which rely on a semipermeable lipid membrane to regulate water transport, alginate hydrogels do not contain a membrane. Instead, the fixed negatively charged carboxylate groups along the alginate chains and their associated mobile ions contribute to the osmotic driving force for water uptake, with swelling continuing until the elastic restoring force of the polymer network balances the osmotic pressure. While the hydrogel is not intended to represent the structure of a cell membrane, it provides a simple experimental system for exploring the principles that govern the movement of nutrients, signaling molecules, and therapeutic compounds through biological environments. In this way, the activity reinforces Biology I concepts related to diffusion, osmosis, and permeability while demonstrating how molecular interactions influence the macroscopic properties of materials.

### Classroom Activities

This activity was designed using standardized national and state-level educational goals (Table 2) and implemented in a local high school during a single 90 min class period, with approximately 70 students participating across three classes. IRB approval (Application number 25-0393, University of Southern Mississippi’s office of research integrity) as well as parental and student consent, was obtained prior to the laboratory activity. Students first completed a pretest designed to gauge their understanding of DoE, ranging from basic questions about the necessity of replicates to more complex questions regarding interactions between variables (all instructional materials and instructor notes are provided in the Supporting Information as Appendix 1 for the laboratory experiment and Appendix 2 for the extension lesson following the laboratory session). Students were then instructed to form groups of four and move to the laboratory space, although we note that the activity could also be adapted for a standard classroom setting. Our goal was to maximize hands-on laboratory time and allow students to actively explore DoE by completing their data tables and comparing results with peers who had different formulation parameters. Each team of four was divided into pairs to complete required characterization assignments within the allotted class period. This structure helped students stay focused while encouraging collaboration and discussion. By analyzing their own measurements alongside those of other teams, students could directly observe how changes in multiple formulation variables affected gel properties, reinforcing the practical application of factorial design, data interpretation, and the iterative nature of scientific and engineering inquiry. Students were then instructed on how to set up their experiments and make alginate gels, with laboratory handouts provided for them to work through. Alginate, calcium chloride, and sodium chloride solutions were premade in bulk prior to student arrival, and groups were assigned a parameter combination from the DoE. Students added food dye to help visualize their gels, and all pairs successfully made alginate hydrogels. One pair of students at the table worked on the mass loss and oversaw monitoring mass loss over time after hydrogels soaked in 5 min increments in concentrated sodium chloride. The other pair synthesized their own hydrogel and used it for the slide test. Following the initial slide down, gels were soaked in sodium chloride solution for 5 min, rinsed and the slide test was repeated (Supporting Information, Video 1). Students then repeated their characterization method and soaking until 20 min remained in the class period (approximately 6 experimental time points assessed). Students then disposed of their materials, washed their glassware, and collected data from their fellow student groups to complete the DoE.

**Table 2:** Pedagogical Goals Addressed by this DoE Alginate Hydrogel Laboratory Activity^a^

Standard/Learning Objective	Pedagogical Goal (Student Learning Outcome)
**BIO.1D** ^17^	Students will describe the **structure of the phospholipid bilayer** and explain how membrane components regulate the movement of substances to maintain **cellular homeostasis**.
**BIO.1D.1** ^17^	Students will **plan and conduct an investigation** demonstrating that cell membranes are **semipermeable**, allowing passive and active transport processes to maintain equilibrium with the environment.
**BIO.1D.2** ^17^	Students will **develop and use models** explaining how cells respond to **hypertonic, hypotonic, and isotonic conditions**, including mechanisms such as the **sodium–potassium pump**.
AP Biology Topic 2.4 – ENE-2.A^18^	Students will describe the **roles of the components of the plasma membrane** (phospholipids, proteins, carbohydrates, cholesterol) in maintaining the internal environment of the cell.
**AP Biology Topic 2.5 – ENE-2.B** ^18^	Students will describe the **mechanisms organisms use to maintain solute and water balance**, including osmosis and osmotic regulation.
**AP Biology Topic 2.6 – ENE-2.C** ^18^	Students will describe the **processes that allow ions and other molecules to move across membranes**, including diffusion, facilitated diffusion, osmosis, and active transport.
**ABET Outcome 1 – Apply Knowledge of Science and Engineering** ^19^	Students will apply principles of **diffusion, concentration gradients, and permeability** to explain how molecules move across biological membranes and comparable synthetic membranes.
**ABET Outcome 3 – Communication of Technical Concepts** ^19^	Students will communicate explanations of **membrane structure and transport mechanisms** using appropriate scientific terminology, diagrams, and models.
**ABET Outcome 6 – Experimentation and Data Analysis** ^19^	Students will design or analyze an experiment demonstrating **diffusion or osmosis across a semipermeable membrane**, interpreting data to explain membrane transport mechanisms.

a Pedagogical goals were derived from standards developed by the Mississippi Department of Education, the College Board AP Biology Course and Exam Description, and the College of Polymer Science and Engineering at The University of Southern Mississippi using ABET outcomes catered to the high-school education level.

Following completion of the lab, students returned to the classroom for the postactivity survey, to gauge how understanding of DoE evolved as a result of this hands-on intervention. While working on the sample analysis questions in a group discussion (Supporting Information Appendix 1), students expressed excitement at being able to change how much alginate and calcium was in the formulations and were able to visualize very different gels in the classroom, which helped tie together that the class collectively was working toward a full factorial DoE. Following the first trial with the highest academic level class, we realized that students had difficulties weighing the hydrogels, so this part of the experiment was removed for the remaining classes to simplify the goals of the activity. Furthermore, clarification was given on aspects of the experiment with scenarios that corresponded to specific short-answer questions within the quiz. The final class was also given a brief 2 min DoE lecture and its usefulness in industry prior to administration of the postquiz. All classes received the same instruction at the end of the laboratory exercise.

### Results from Intervention and Quiz Analysis

The prequiz and postquiz given to students consisted of seven questions and were divided into two sections including multiple choice/short answer (Q1–4) and conceptual understanding (Q5–7) (Pre/Post Quiz, Supporting Information Appendix 3). The quiz questions were designed using the Experimental Design Ability Test (EDAT) to ensure proper format of questions administered.^14^ Responses were collected and analyzed; the accuracy of short-answer questions was assessed by examining key phrases and overall comprehension. Comparisons of pre- and postexperiment quiz performance indicated minimal differences in response accuracy (Figure 4). The mean of the multiple-choice and short-answer sections decreased, while the mean of the conceptual understanding section increased, resulting in a postquiz average similar to the prequiz average.

**Figure 4:**
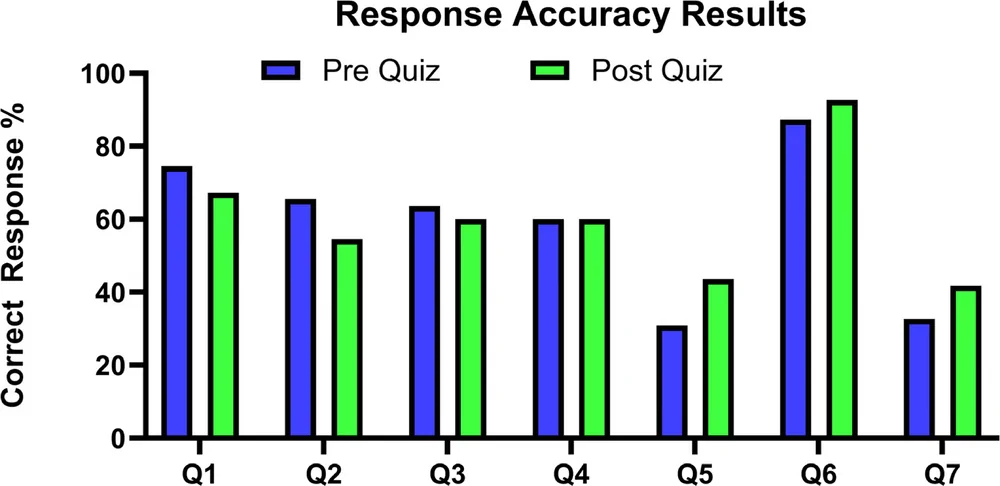
Percentage of accurate responses per question compiled from all three classes.

Although the overall accuracy of student responses did not change significantly between the pre- and postquizzes, the percentage of students who attempted the short-answer questions increased from 62.4% in the prequiz to over 86% in the postquiz (Figure 5). This increase suggests that students became more comfortable engaging with the concepts presented during the experiment and were more willing to provide a response even when they were unsure of the correct answer. While a greater response rate does not necessarily indicate an improved understanding, it does demonstrate increased confidence in attempting to apply newly introduced DoE concepts. All multiple-choice questions were answered; therefore, only answers that required writing were included in the total response rate percentage displayed.

**Figure 5:**
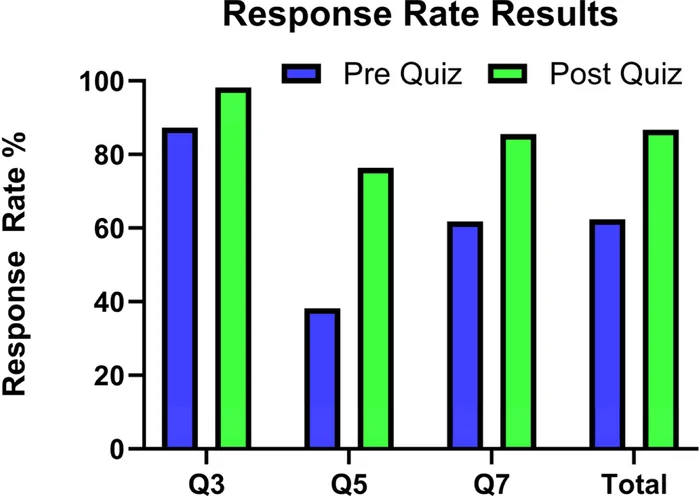
Percentage of response rate for only short answer responses and total of the three short answer questions.

Notably, the median accurate response rate of the questions designed to probe conceptual understanding (Q5–Q7 in pre/post quiz, Supporting Information Appendix 3), shifted from 33.33% in the prequiz to 66.67% in the postquiz. Although this increase was not reflected in a significant improvement in overall quiz accuracy, it suggests that students became more comfortable attempting conceptual questions and explaining their reasoning. Overall, the results from this sample size indicate that the experiment positively stimulated students’ critical thinking capabilities targeted at a topic atypically taught at the high school level. When considering the trends in data between pre- and post quizzes, it is important to note biases that could arise based upon changes in initial exposure to the DoE topic, as degree of exposure did change in the last group prior to the administration of the prequiz. However, the decision to change exposure and include a preliminary brief lecture was implemented to improve the design of instruction for the academic level of the students, as ensuring the greatest opportunity for educating students was and is always the highest priority in a classroom setting.

Despite the minor changes between the groups, all groups of students were very excited and engaged with the project from start to finish. For instance, one student on the prequiz who answered “idk” in response to *if you only tested one factor at a time, what kind of conclusion would you miss compared to a factorial design* (Question 7, pre/post quiz, Supporting Information Appendix 3) later recorded a response in the postquiz of “an effect from altering both temperature and pH simultaneously.” Another student who also initially responded “idk” to the same question, answered in the postquiz with the following: “how the 2 factors changed together and found how they affected each other.” Another student initially answered, “you wouldn’t really miss anything,” but later responded on the postquiz that “you would miss a lot of measurements.” Although this answer is incorrect in response to the question specifically, it does demonstrate the student’s recognition of the importance of DoE for systematically gathering information and understanding experimental outcomes. These qualitative examples highlight the increased willingness of students to engage with the concepts introduced during the activity. Although not every response was completely correct, many students progressed from providing no answer to attempting to explain their reasoning using ideas discussed. These observations suggest that exposing students to DoE, even at an introductory level, provides a valuable opportunity to develop scientific reasoning and familiarity with a topic that is not commonly taught at the high school level.

### Limitations and Future Works

Limitations in this project include a limited sample size of students and demographics limited to a single school within the district. The school district has an overall population consisting of 73% White, 20% Black or African American, 3% Hispanic or Latino, 1% Asian, and 2% two or more races as indicated by census data.^20,21^ A larger sample size that incorporates a diverse demographic of students from multiple school districts would strengthen findings on the efficacy of the experiment. As previously mentioned for the activity, the hydrogels became more difficult for students to handle and accurately weigh as swelling increased over time. Ultimately, this part of the experiment was abandoned after the first group’s attempt. In future versions of the activity, increasing the initial alginate concentration across all samples may help the hydrogels retain their shape and improve handling during mass measurements. Decreasing the amount of time the hydrogels remain in solution between measurements may also reduce excessive swelling and improve the accessibility of collecting data.

As a goal of this lesson was to continue improving the students’ understanding of DoE, the amount of exposure was not standardized and varied from class to class between prequiz and postquiz, impacting the performance. While this is noteworthy to include and can negatively skew differences in pre- and postquiz results, the aim was to iteratively improve the overall experiment to facilitate successful student interaction with an experimental process traditionally unfamiliar within the classroom setting. Future work could be to increase and diversify the number of questions on the pre- and post- quiz to expose gaps of learning for the ability to tune the project for various academic levels. Additionally, future iterations of the lesson plan might benefit from integrating one-factor-at-a-time (OFAT) testing to serve as a comparison to the DoE framework. Each class could be split into two groups for lesson completion; one performing a DoE while the other conducts an OFAT study. Data related to experimental time and resource use would be logged by each group throughout the lesson, with a class discussion to follow. This would provide a clear comparison of the time and resource savings that accompany a DoE compared to OFAT designs, with students leading the discussion about what they observed during their respective experimental processes. We predict such a comparison would further strengthen student comprehension of the purpose and benefits of DoE in addition to serving as a transition into the accompanying lecture on DoE use in industry. A more robust lecture that connects student observations to industrial use of DoE is recommended, in addition to a lecture prior to experimentation that introduces DoE comparatively to OFAT testing, the latter of which students have existing knowledge of. Lecture topics should include the connection of DoE to systematic data collection and trend recognition, important factors of AI and ML tools that industry commonly utilizes to enhance workflows, identify areas of process improvement, and save time and costly resources. As a check-in activity prior to engaging in the lesson, students could identify potential resource constraints associated with hydrogel synthesis given information learned from the prelecture and lesson introduction. Finally, comparing students in different STEM disciplines, i.e. a chemistry course compared to students in a pre-engineering course could allow for analysis of how different STEM courses approach similar problems would pose an excellent discussion on how different coursework affects students’ perspective in critical thinking applications.

## Conclusions

Design of experiments is a valuable tool used across STEM fields to systematically and efficiently capture scientific data. High school students’ established understanding of the traditional single-variable, hypothesis-driven scientific method can be transitioned into a discussion of the importance of DoE, through which multiple scientific variables can be altered simultaneously. A pilot DoE lesson implemented with approximately 70 high school biology students allowed for experiential learning about polymer hydrogels and how synthesis factors can be adjusted in tandem to draw conclusions about resultant material properties. Students expressed excitement when observing differences between their hydrogel formulations and their ability to alter multiple synthesis factors at the same time. Comparison of the pre- and postassessments illustrated a 23.6% increase in conceptual understanding following the lesson, and the median correct response rate for this section shifted from 33.33% to 66.67%. While no statistically significant results were found pertaining to answer accuracy, the lesson qualitatively encouraged students to think more critically about polymer hydrogels and their relationship to the DoE methodology, as demonstrated by increased engagement with short-answer conceptual questions. This study provides a foundation for future lesson plans that expose STEM students to industry tools for data collection, such as DoE, and highlights the connection between classroom content and real-world applications. Importantly, integrating DoE concepts also introduces students to foundational ideas underlying AI and ML, supporting a key goal of modern curriculum design to prepare students for the future STEM workforce.

## Supplementary Material





## Notes

Supporting Information: , and a . The Supporting Information is available at https://pubs.acs.org/doi/10.1021/acs.jchemed.6c00856.

Alginate DoE laboratory guide, alginate DoE Extension lesson, and pre and post surveys (PDF)
Video of the hydrogel slide being conducted during the laboratory activity (MP4)
