# Supplementary material for: The Science of Design: Introducing Design of Experiments (DoE) through a Hydrogel-Based High School Chemistry Investigation
Source: J Chem Educ. 2026 Sep 9;103(10):5689–97. doi: 10.1021/acs.jchemed.6c00856 (PMC13674000; doi:10.1021/acs.jchemed.6c00856)
Supplement: Supplementary file 1 [file ed6c00856_si_001.pdf]

Supporting Information

The Science of Design: Introducing Design of Experiments (DoE) Through a Hydrogel-Based High School Chemistry Investigation

Penelope E. Jankoski,[1#] Jamie Sorrell,[2#] Harrison Livingston,[1] Dori Harcharik,[1] Claire Clemons,[1] Tristan D. Clemons.[1*]

1 School of Polymer Science and Engineering, University of Southern Mississippi, Hattiesburg, MS, 39406, USA.

2 Sumrall High School, Sumrall, MS, 39402, USA.

Table of Contents

Appendix	Title	Page
Appendix 1	Alginate DoE Hydrogel Laboratory	2
Appendix 2	Leveraging Passive Diffusion for Drug Delivery applications – Extension lesson following the DoE laboratory.	11
Appendix 3	Pre/Post Student Quiz: Full Factorial Experimental Design of Experiments	41

Appendix 1

Alginate DoE Hydrogel Laboratory

POLYMER SCIENCE AND ENGINEERING
AT SOUTHERN MISS

LEVERAGING DIFFUSION FOR DRUG DELIVERY WITH THE ASSISTANCE OF HYDROGELS

TOPIC	Hydrogels	DURATION	90min Class Block
GRADES	9-12th	DATE	Insert Text

SUMMARY

In this experiment, students will create hydrogels using sodium alginate (a polymer from seaweed) and calcium chloride. Hydrogels are soft, jelly-like materials that can hold large amounts of water and trap substances inside, like calcium ions.

Students will test how different concentrations of alginate and calcium affect:

- How much calcium is released over time
- How strong or stable the gel remains

To do this, students will soak the gels in a saltwater solution (like a simulated body fluid) and use test strips to measure the calcium released every 5 minutes. They'll also test the gel's physical strength by tilting it on a ramp and observing how it holds its shape or slides.

This activity models a real-world application:

Hydrogels are used in drug delivery systems to slowly release medicine in the body over time.

In this lab, calcium ions represent the "medicine" being released. By adjusting the gel's ingredients, scientists can control how fast or how slowly the drug is released — just like students will observe in their experiments.

LEARNING OBJECTIVES

Science Content Objectives:

1. Describe how ions like calcium move from a hydrogel into a surrounding solution through diffusion.
2. Explain how a hydrogel forms through crosslinking of polymers with calcium ions.
3. Understand the relationship between polymer structure and material strength.

Experimental Design Objectives:

1. Carry out a full factorial experiment, testing all combinations of two variables (calcium and alginate concentrations).
2. Record and analyze both quantitative data (Ca^{2+} in ppm, slide speed) and qualitative observations (gel shape, softness).
3. Compare how different variable combinations affect the response variable (calcium ion release).
4. Propose improvements or next steps based on their findings (e.g., choosing a new concentration to test).

Critical Thinking Objectives:

1. Use evidence to support claims about material performance.
2. Predict how changing a factor (e.g., increasing calcium concentration) might affect gel behavior.
3. Connect this model system to real-world uses of hydrogels, such as drug delivery or wound care.

MATERIALS NEEDED

Chemicals

- Sodium Alginate (food or lab grade)
- Calcium Chloride ($CaCl_2$)
- Salt (NaCl)
- Distilled or Deionized Water

Glassware/Plasticware

- Petri Dishes
- Graduated Cylinders
- Plastic or Metal Spatulas
- 60mL Syringe

Weighing and Mixing

- Digital Scale (0.01 g sensitivity)
- Weigh boats
- Stirring rods

Testing Gel Strength

- Tilt testing surface (e.g., clipboard or tray)
  - Set at 30–45° incline using books or blocks
- Ruler (marked in cm)
- Timer or stopwatch

NEXT GENERATATION SCIENCE STANDARDS

BHS-PS1-5
Apply scientific principles and evidence to provide an explanation about the effects of changing the temperature or concentration of the reacting particles on the rate at which a reaction occurs.

HS-PS1-2
Construct and revise an explanation for the outcome of a simple chemical reaction based on the outermost electron states of atoms, trends in the periodic table, and knowledge of chemical properties.

HS-PS2-6
Communicate scientific and technical information about why the molecular-level structure is important in the functioning of designed materials.

HS-ETS1-3
Evaluate a solution to a complex real-world problem based on prioritized criteria and trade-offs

HS-LS1-3
Plan and conduct an investigation to provide evidence that feedback mechanisms maintain homeostasis.

HS-ETS1-2
Design a solution to a complex real-world problem by breaking it down into smaller, more manageable problems.

CURRICULUM INTEGRATION

Chemistry

- **Ionic bonding and ion exchange (Ca^{2+} and Na^{+})**
- **Polymer structure and crosslinking**
- **Solution concentration (mM, % w/v, ppm)**

Biology

- **Cell membrane analogs (diffusion through networks)**
- **Biomedical applications (drug delivery, wound healing)**

Mathematics

- **Data analysis**
- **Graphing calcium release vs. time**
- **Calculating gel slide speed (distance/time)**

Engineering & Design Thinking

- **Biomedical engineering**
- **Full factorial design**

Health / Biomedical Connections

- **Drug delivery systems**

KEY VOCABULARY

Hydrogels
Ion Exchange
Ionic Crosslinking
DIffusion
Polymer
Sodium Aglinate
Concentration
Drug Delivery

CREATED BY: Jamie Sorrell

SCHOOL OF POLYMER SCIENCE AND ENGINEERING SOUTHERN MISS

Leveraging Diffusion for Drug Delivery with the Assistance of Hydrogels
Stock Solution Instructions

TEACHER PREP

1000mL Glucose/Salt Solution Directions for Body Fluid Simulation

Each group needs 400mL of Solution

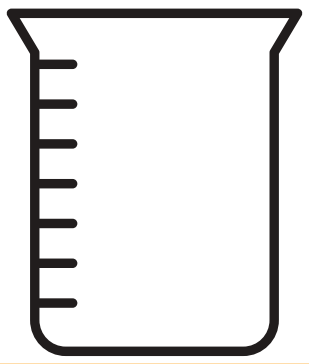

In a 1L Beaker Mix: 1000mL of Water, 190 g of Salt

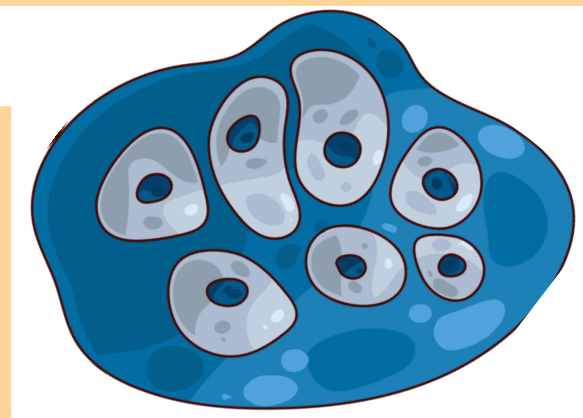

Sodium Alginate Stock Solution Directions: **This solution needs to be prepared AT LEAST 24 Hours in Advance.**

$$\frac{?\%}{100-1} * 1000 = ?\text{g of Sodium Alginate}$$

(?g of Sodium Alginate)/4 to make a 250mL Solution

Low	Medium	High
1 % w/v concentration	1.5 % w/v concentration	2 % w/v concentration
2.53g of Sodium Algniate	3.79g of Sodium Algniate	5.05g of Sodium Algniate

Procedure:

1- Add 125mL of Distilled Water to a 500mL Beaker
2- Weigh out the Sodium Alginate according to the above chart
3- Add the Alginate (try to add it to the center of the water, so solid does not stick to the sides) and stir with a glass rod.
4- The Alginate will form flaky clumps
5- Add more water until it reads 250mL on the beaker.
6- Allow the mixture to sit overnight to give a uniform solution that will turn into a viscous consistency.
After 24 hours if solid still remains, constant stirring and heat will help dissolve the solid into solution.**

500mL Calcium Chloride Stock Solution Directions:

$$?M * 110.98 = ?\text{g of Calcium Chloride}$$

(?g of Calcium Cholride)/4 to make a 250mL

High	Medium	Low
0.1 M Concentration	0.05 M Concentration	0.02 M Concentration
2.77g of Calcium Chloride	1.39g of Calcium Chloride	0.55g of Calcium Chloride

Procedure:

1- Add 125mL of Distilled Water to a 500mL Beaker.
2- Weigh out the Calcium Chloride according to the above chart.
3- Add the Calcium Chloride and stir with a glass rod until all is dissolved.
4- Add more water until it reads 250mL on the beaker.
After 24 hours if solid still remains, constant stirring and heat will help dissolve the solid into solution.

Lab Equipment per Group

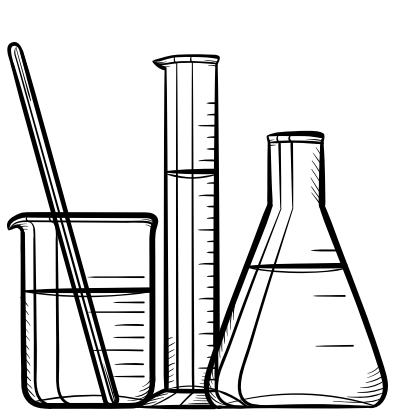

1-60mL Syringe
2-Disposible Pipettes
2- 100mL Graduated Cylinder
2- Petri Dishes
1-Vial of Food Coloring
1- Scoop
1-Balance

Hydrogel Strength Test Parts

1- Laminated Distance Sheet
1- Ruler
1- Text Book to create a slope
1- Timer (or Cell Phone)

Lab Introduction:

Sodium Alginate can be ionically crosslinked with Calcium Chloride to make a Hydrogel.
What Happens?

Na+ ions are displaced by exchanged with Ca+2 ions

The Ca+2 ions **Chelate** with the Alginate creating a physical **ionic crosslinked** network.

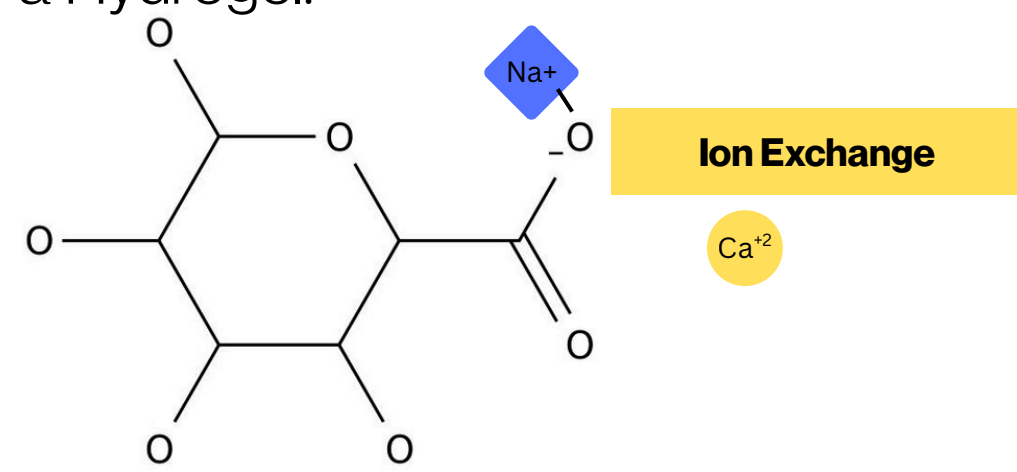

Water forms hydrogen bonds with the polymer backbone and **Water can get physically trapped within the space of the network.**

If there is excess/free Ca+2 (that were not crosslinked), they can also get trapped in the network.

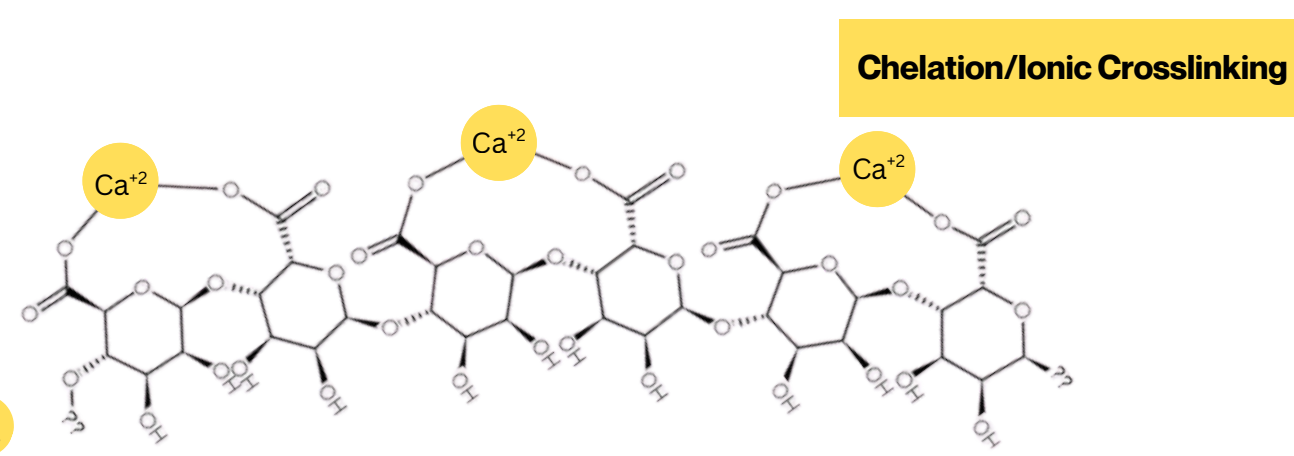

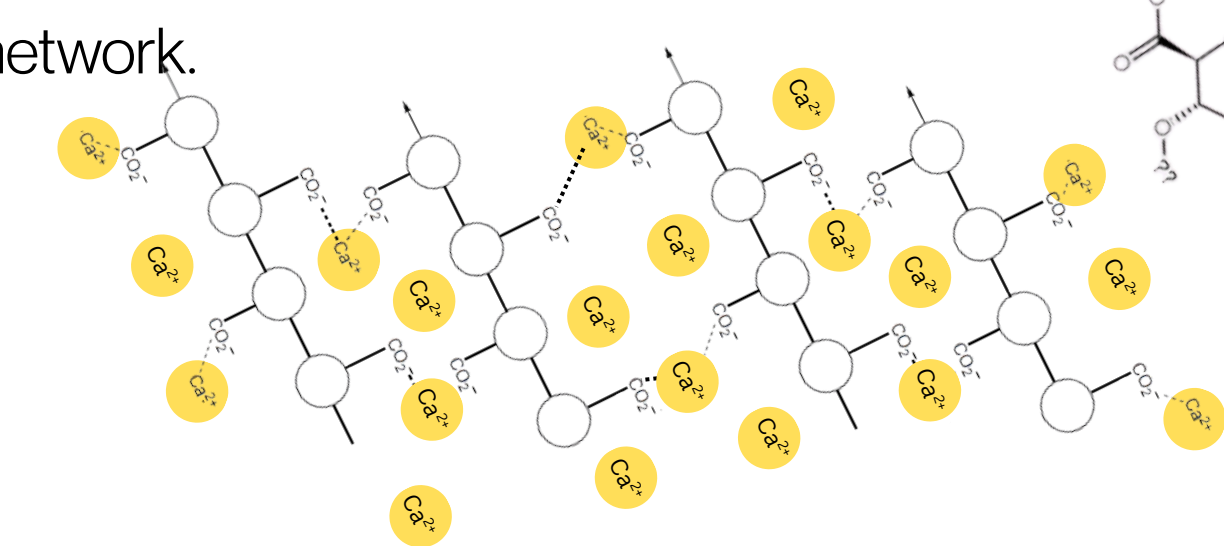

Lab Purpose:

Just like free Ca^{2+} ions can get trapped in hydrogels, biomedical engineers can encapsulate medications within hydrogels for drug delivery. To make sure the medicine is released at the right speed and is safe for the body, the hydrogel has to be carefully made with the right ingredients and structure.

How does IT Work?? Materials (medications and Ca+2) trapped in the hydrogel will be released from the network either due TO

1- **DIffusion**

2- **Ion Exchange**

3- the Hydrogel breaking down

4-pH

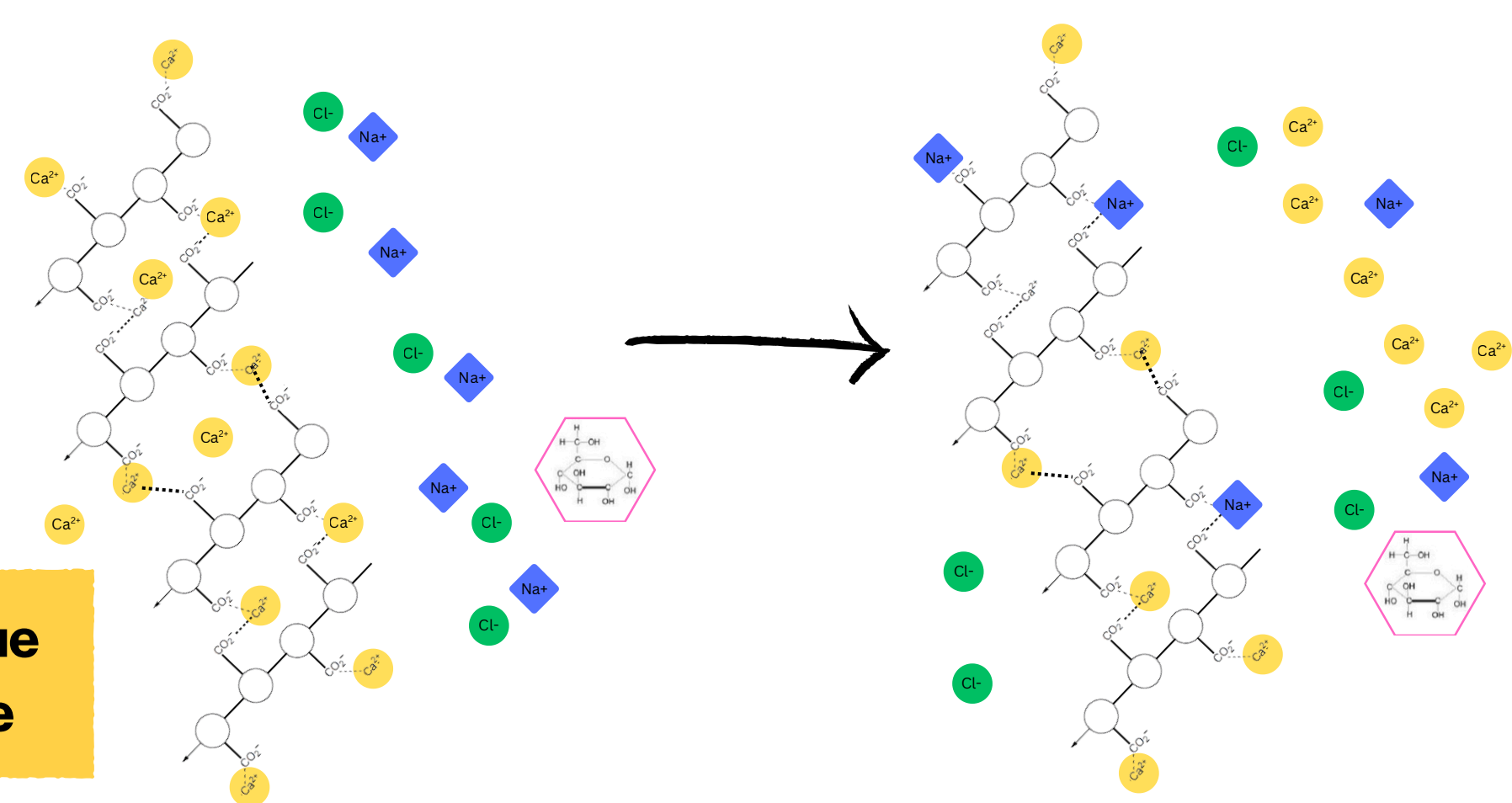

Ca+2 release into body fluid due to Diffusion and Ion Exchange

In this lab, you will act as a bioengineer.

You will be making hydrogels using **different concentrations of calcium ions (Ca^{2+}) and alginate.**

Your goal is to figure out which combination creates a hydrogel that releases calcium slowly over time Calcium release can be measure by the rate at which the hydrogel breaking apart overtime.

This is important because hydrogels like these can be used to slowly release medicines inside living organisms.

Leveraging Diffusion for Drug Delievery with the Assistance of Hydrogels
Full Factorial Design and Sample Set-up

We will be conducting a **Full Factorial Design Experiment** — a method scientists and engineers use to test all possible combinations of different factors that might affect the outcome of an experiment.

This type of design helps us:

- See which factor has the biggest effect on the results,
- Observe how factors work together or cancel each other out, and
- Create an optimal setup or design based on what we learn.

Set-up 1

Factor 1: Calcium Concentration **Factor 2: Alginate Concentration**

We will have 9 different sample

- **0.02M CaCL 1 wt% Alginate**
- **0.02M CaCL 1.5 wt% Alginate**
- **0.02M CaCL 2 wt% Alginate**
- **0.05M CaCL 1 wt% Alginate**
- **0.05M CaCL 1.5 wt% Alginate**
- **0.05M CaCL 2 wt% Alginate**
- **0.1 M CaCL 1 wt% Alginate**
- **0.1 M CaCL 1.5 wt% Alginate**
- **0.01 M CaCL 2 wt% Alginate**

Sample Set-up

Assigned Sample: ☐ M of **Calcium Chloride** ☐ % wt of **Alginate**

Sample Prep Procedures:

1. Using a 60mL syringe measure out 20mL of Sodium Alginate and add it to the BOTTOM of a Petri Dish
2. Using the Graduated Cylinder meaure out 30mL of Calcium Choride
3. Add 1 drop of food coloring to the Calcium Choride in the Graduated Cylinder use a scoop to stir unti the food coloring is dissolved in the solution
4. Add the Calcium Chloride to the Petri Dish of Alginate.
5. Carefully Mix with the scoop or spatuala
6. Let it stand for 5 mins.

Data Collecting Procedures:

1. Using a pipette carefully remove the excess liquid from the Petri Dish make sure to not remove any of the hydrogel.
2. Weigh your sample IN the petri dish using the electronic balance- Record as your starting weight.
3. Carefully remove the Gel using the Scoop and perform the Hydrogel Strength Test (Sliding Speed).
4. Document the Gel's Initial Speed (0 mins) and Qualitative Observations on the data sheet.
5. Carefully tansfer the Gel back to the Petri Dish using the Scoop.
6. Add 50mL of Salt Solution ("Body Fluid") to the gel in the Petri Dish using the Graduated Cylinder
7. Set timer for 5 mins.
8. After 5 mins, REPEAT Steps 1-7 every 5 mins until completing the Data Table

In the human body, fluids around cells are always moving. To model this in our experiment, we'll remove the Salt Solution ("Body Fluid") every 5 minutes and replace it with fresh solution. This helps us simulate how the body constantly replaces and filters fluids to keep things balanced and healthy.

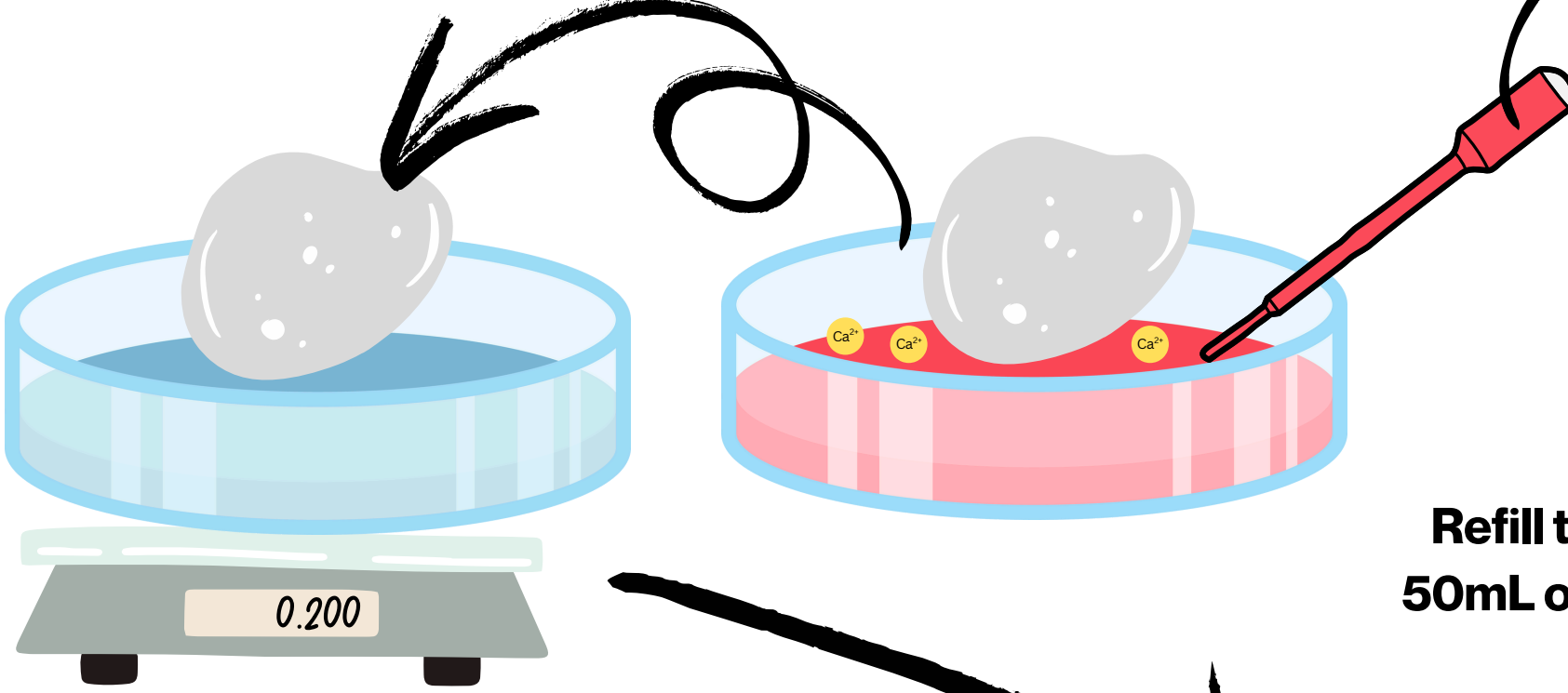

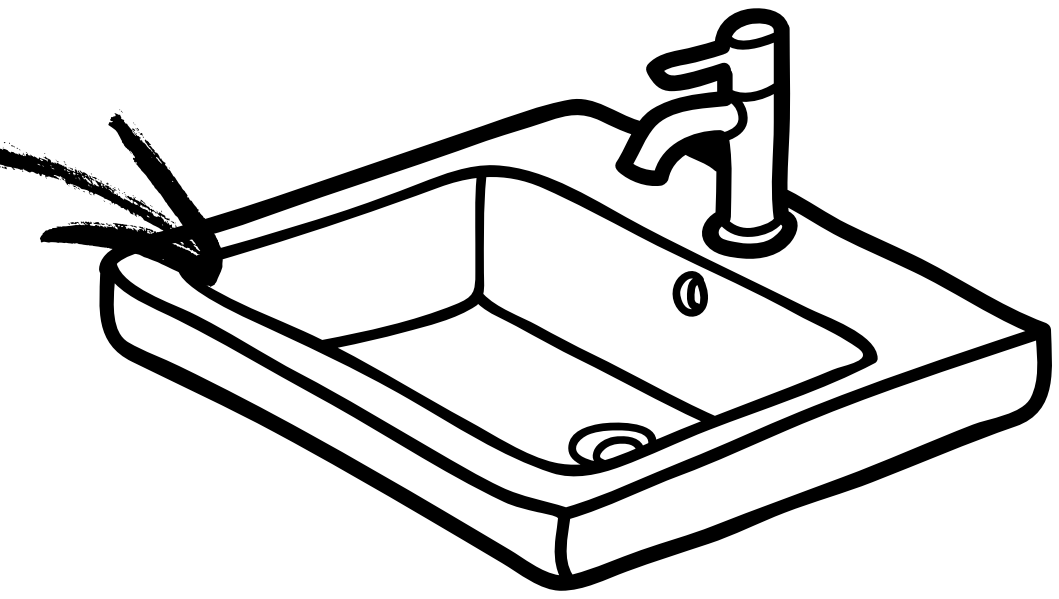

Refill the Petri Dish with 50mL of New Salt Solution

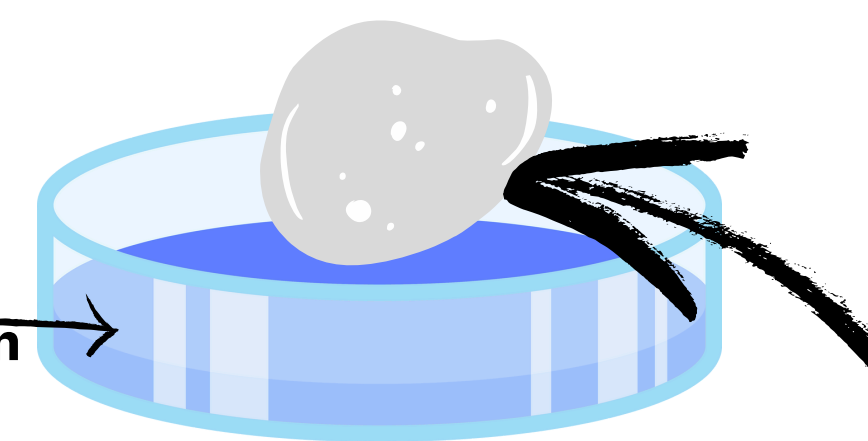

Observation #1 – Initial Behavior:
Note if the hydrogel starts sliding immediately or stays in place.

Observation #2 – Sliding Motion:
Start the timer if it begins to slide.
How long does it take for it move 20cm?

Observation #3 – Deformation:
Watch for changes in shape — does the hydrogel stretch, break apart, or stay intact?
Record whether it leaves any residue or sticks to the surface.

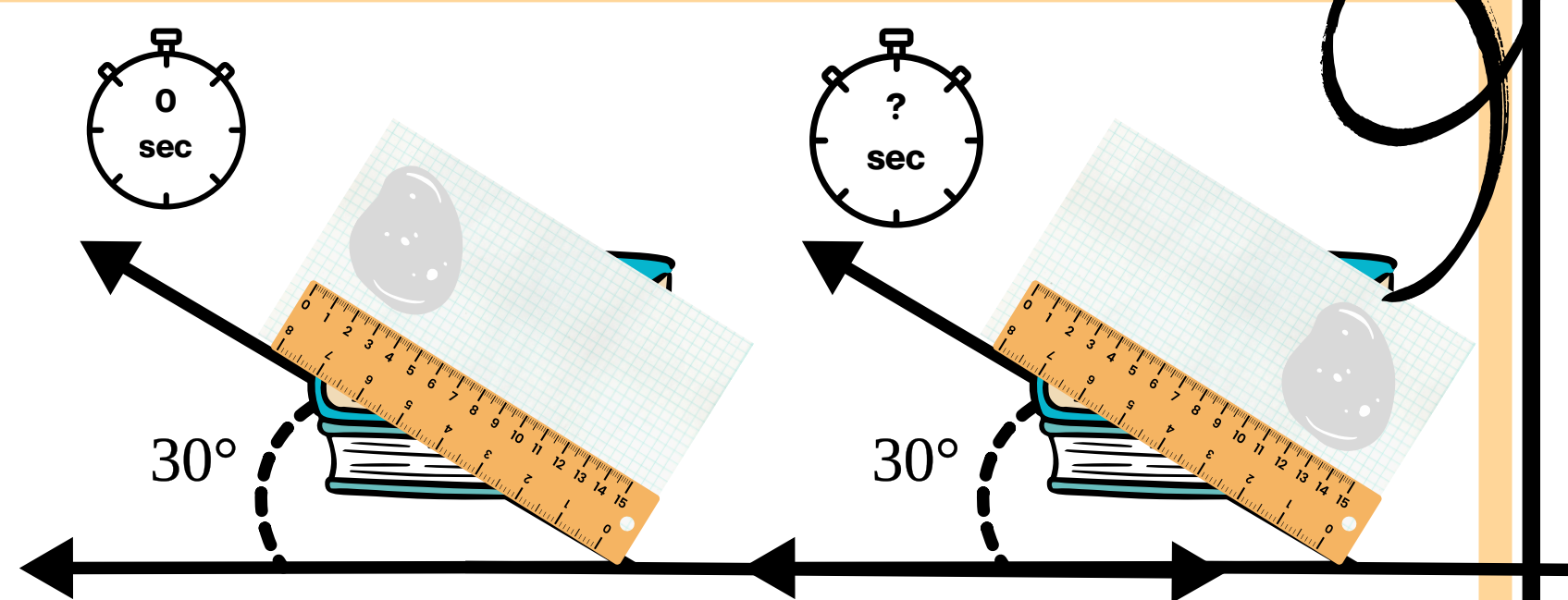

☐ M **CaCl** & ☐ % wt of **Alginate**

Time (min)	Mass (g)	Hydrogel Slide How long to reach 20cm?	Kept Shape Y or N	Slid Smoothly without Deforming Y or N	Deformed or Broke Apart
START 0					
5					
10					
15					
20					
25					
30					

Leveraging Diffusion for Drug Delivery with the Assistance of Hydrogels

Group Data Table

Hydrogel Slide Speed Overtime:

Time	0.02M CaCl 1%w/v of Alginate Gel Slide Speed cm/sec	0.02M CaCl 1.5%w/v of Alginate Gel Slide Speed cm/sec	0.02M CaCl 2%w/v of Alginate Gel Slide Speed cm/sec	0.05M CaCl 1%w/v of Alginate Gel Slide Speed cm/sec	0.05M CaCl 1.5%w/v of Alginate Gel Slide Speed cm/sec	0.05M CaCl 2%w/v of Alginate Gel Slide Speed cm/sec	0.1M CaCl 1%w/v of Alginate Gel Slide Speed cm/sec	0.1M CaCl 1.5%w/v of Alginate Gel Slide Speed cm/sec	0.1M CaCl 2%w/v of Alginate Gel Slide Speed cm/sec
0									
5									
10									
15									
20									
25									
30									

Change in Hydrogel Mass:

Time	0.02M CaCl 1%w/v of Alginate Mass (g)	0.02M CaCl 1.5%w/v of Alginate Mass (g)	0.02M CaCl 2%w/v of Alginate Mass (g)	0.05M CaCl 1%w/v of Alginate Mass (g)	0.05M CaCl 1.5%w/v of Alginate Mass (g)	0.05M CaCl 2%w/v of Alginate Mass (g)	0.1 M CaCl 1%w/v of Alginate Mass (g)	0.1 M CaCl 1.5%w/v of Alginate Mass (g)	0.1 M CaCl 2%w/v of Alginate Mass (g)
0									
5									
10									
15									
20									
25									
30									
Broke Apart Y or N									

Sample Analysis Questions

Name ____ Block _____________

Which sample released the most calcium? Which released the least? What patterns do you notice between calcium release and alginate concentration?

Did the hydrogel release all the calcium that was originally inside the gel, or did some calcium stay trapped? How can you tell based on your data?

How did changing the calcium concentration affect the stiffness or strength of the hydrogel? Did any samples break down or fall apart?

How did changing the alginate concentration affect how strong the hydrogel was? Which samples were too soft or too firm?

Which samples showed a slow, steady release of calcium without falling apart too quickly? Why do you think those were the best?

What is the best combination of calcium and alginate concentration for a drug-delivery hydrogel? Explain your choice using evidence from your observations.

Why is it important for a hydrogel to release calcium (or medicine) slowly instead of all at once? What could happen if the release was too fast?

In real medicine, a hydrogel must be strong enough to stay in the body but still allow drugs to escape over time. Which of your samples came closest to doing both?

How could you improve this experiment to better test calcium release or hydrogel strength in a real-body environment?

Resources:

Sodium Alginate: https://www.flinnsci.com/sodium-alginate-25-g/s0445/

Calcium Chloride: https://www.flinnsci.com/calcium-chloride-reagent-powder-100-g/c0196/

Reference:

Padron, F. J. (2022). Students experiment with hydrogel designs in the laboratory [Image]. In To Infinity and Beyond: The Amazing Hydrogels – Polymers Activity. TeachEngineering, Rice University. Retrieved June 12, 2025, from https://www.teachengineering.org/activities/view/rice-2643-amazing-hydrogels-polymers-activity teachengineering.org

Lab Safety:

- Safety Googles

Disposal- Polymer gel products may be disposed of in the trash. Excess Calcium Chloride, Salt/Glucose Solution, and Salt/Glucose Solution with Ca+2 may be disposed of down the drain with plenty of excess water.

Appendix 2

Leveraging Passive Diffusion for Drug Delivery applications – Extension lesson following the DoE laboratory.

LEVERAGING DIFFUSION FOR DRUG DELIVERY WITH THE ASSISTANCE OF HYDROGELS

TOPIC	Passive Transport: Diffusion	DURATION	90min Class Block
GRADES	9-12th - Biology	DATE	2025

SUMMARY

This lesson introduces students to the concept of diffusion as it occurs in both chemical solutions and biological systems, highlighting the role of concentration and electrochemical gradients. Students start with a visible demonstration of diffusion using food coloring in water, then explore increasingly complex models of diffusion, including cell membranes, ion movement, and hydrogel systems.
Using interactive simulations, graphics, and a real-world full factorial design experiment involving calcium alginate hydrogels, students investigate how diffusion is influenced by both chemical concentration and electrochemical forces. The lesson culminates in a discussion of how these principles are applied in drug delivery technologies, where diffusion and cell gradients control how medication enters cells.

LEARNING OBJECTIVES

By the end of this lesson, students will be able to:

1. Describe Passive Transport including: diffusion and faclitated diffusion as particle movement from high to low concentration.
2. Explain how chemical gradients and electrochemical gradients affect diffusion across membranes.
3. Interpret a graphic model of electrochemical gradients and relate it to ion movement.
4. Use a simulation to visualize all associated concepts with Passive Transport.
5. Analyze how calcium diffuses from an alginate hydrogel in salt solution when applying a Full Factorial Design Experiment.
6. Apply their understanding to explain how hydrogels can deliver drugs in a systamic way to cells influenced by diffusion and electrochemical gradients.

MATERIALS NEEDED

Engage: Food coloring and beaker of water
Explore: Electrochemical Gradient Graphic
Explain: Chromebooks to access the Cell Membrane Phet Simulation and Simulation Direction Cards, Special Note- Print a classroom set of cards on cardstock (1 set for every student or 1 set for students to work in pairs.
Elaborate: Lab Supplies Associated with the Full Factorial Design Experiment (Detail List is provided Lab Set Up Instructions Attached)
Evaluate: Scenario Cards

Attached Student Handouts to help students navigate through the lesson.

MISSISSIPPI COLLEGE AND CAREER READINESS STANDARDS

Biology I Standards:
Conceptual Understanding: The structure of the cell membrane allows it to be a selectively permeable barrier and maintain homeostasis. Substances that enter or exit the cell must do so via the cell membrane. This transport across the membrane may occur through a variety of mechanisms, including simple diffusion, facilitated diffusion, osmosis, and active transport.

BIO.1D Students will describe the structure of the cell membrane and analyze how the structure is related to its primary function of regulating transport in and out of cells to maintain homeostasis.

BIO.1D.1 Plan and conduct investigations to prove that the cell membrane is a semi-permeable, allowing it to maintain homeostasis with its environment through active and passive transport processes.

BIO.1D.2 Develop and use models to explain how the cell deals with imbalances of solute concentration across the cell membrane (i.e., hypertonic, hypotonic, and isotonic conditions, sodium/potassium pump

CURRICULUM INTEGRATION

- **Biology**: Cell membrane transport, passive and facilitated diffusion, electrochemical gradient.
- **Chemistry**: Molecular motion, solution concentration, diffusion in materials, ionic interactions.
- **Applied Science**: Drug delivery systems using hydrogels.

KEY VOCABULARY

Cell Membrane	Passive Transport
Phospholipid Bilayer	Diffusion
Phosphate Head	Concentration/Chemical Gradient
Hydrophilic	Equilibrium
Lipid/Fatty Acid Tails	Facilitated Diffusion
Hydrophobic	Ions
Transport/Gate Protein	Electrochemical Gradient
Concentration	Electrical Gradient

CREATED BY: Jamie Sorrell

SCHOOL OF POLYMER SCIENCE AND ENGINEERING SOUTHERN MISS

ENGAGE

The teacher will set up the **Food Coloring Diffusion Demo**. See set-up below:

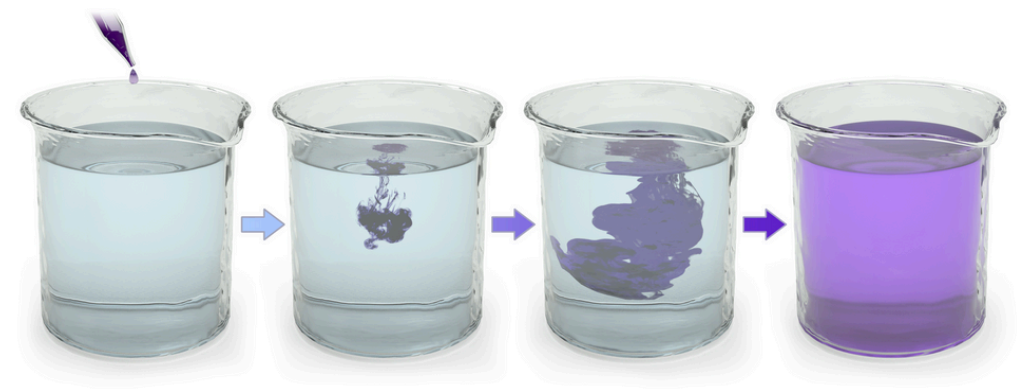

Diffusion

Expii. "Diffusion (Passive Transport) — Definition & Examples." Expii, https://www.expii.com/t/diffusion-passive-transport-definition-examples-10098. Accessed 5 June 2025.

Purpose: The teacher introduces the concept of **Passive Transport** in which materials naturally move from High to Low Concentration with the chemical concentration gradient.

Independently: Students will document their observations on their **Guided Notes** and their answers to the following questions:

What happens when I dropped the food coloring into the still water?

Did the food coloring spread out in the water without stirring?

Did any visual factors affect how fast food coloring diffuses in water?

Was any heat, electicity or energy necessary for food coloring to spread in water?

Concept: **Passive Transport:** Materials naturally move from High to Low Concentration without the use of energy. Materials move with the concentration gradient without energy.

EXPLORE

The teacher will introduce the BEFORE and AFTER Graphics below:

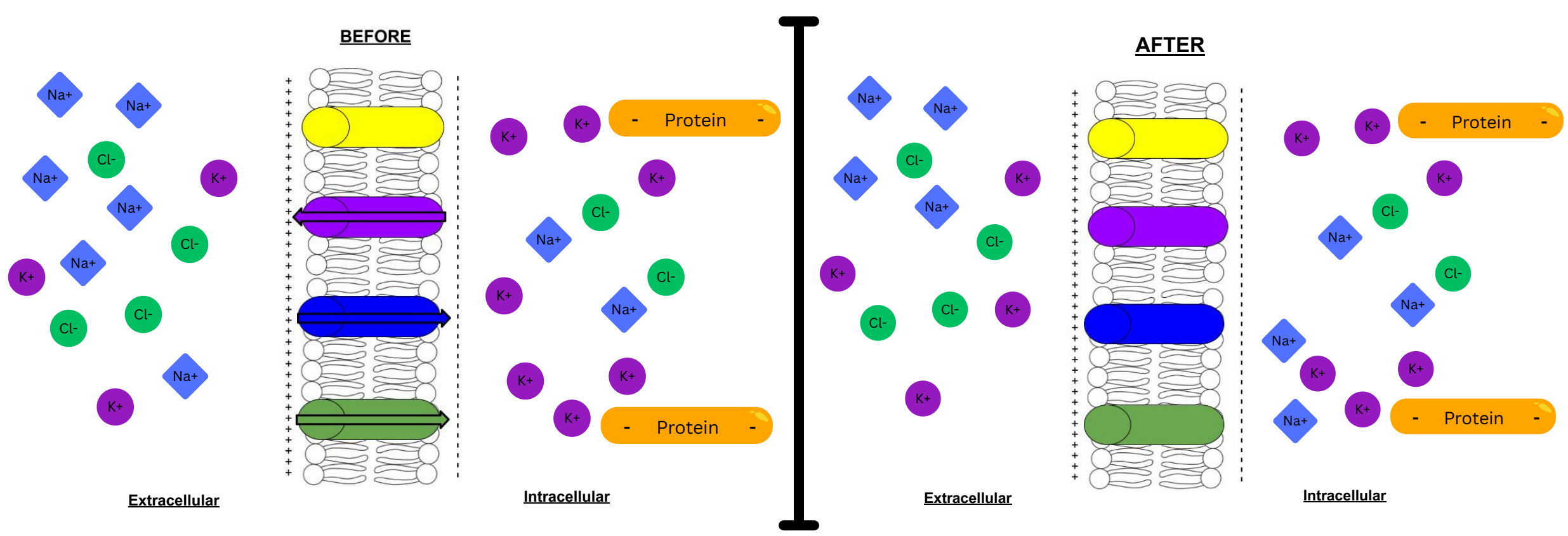

The students will document their observations on their **Guided Notes** after comparing the BEFORE and AFTER Graphics.

Expected observations:

-There are different protein channels

- Na+ moved from high to low, and ends with 4 Na+ on either side, equal
- K+ move from high to low, but it is not equal on both sides
- Cl- did not move through the transport protein
- Proteins remained on the inside
- Proteins and Cl- have a negative charge and the Inside of the Cell has a negative charge
- K+ and Na+ have a positive charge and the outside of the Cell has a positive charge

Allow time for students to compare and share their observations/fiindings

EXPLAIN

Students will use **Simulation Direction Cards** that include guided questions to progress through a simulation of cell transport through a cell membrane. They will document their findings on their Guided Notes

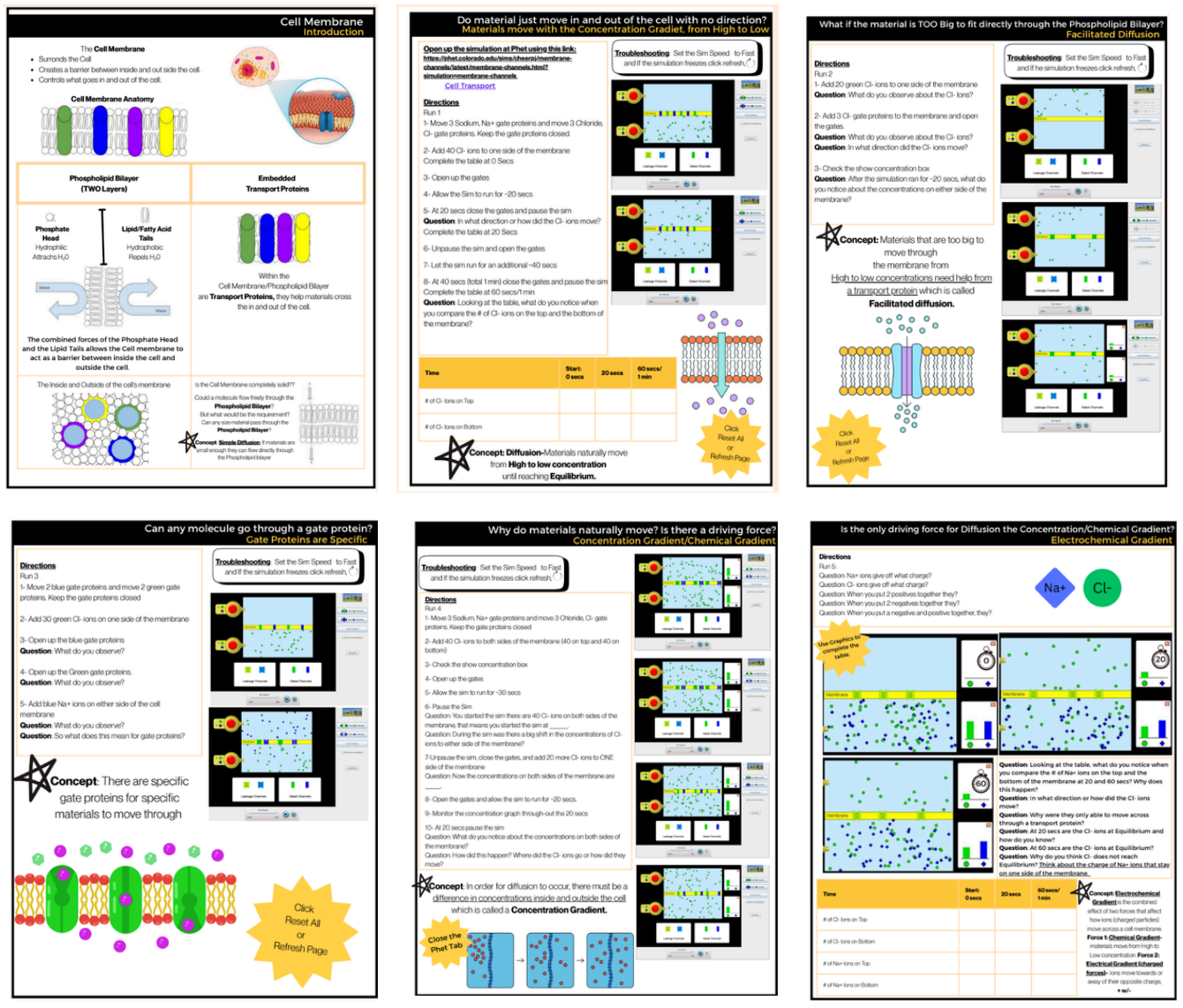

Key Vocabulary and Concepts

-Selectively Permeable Membrane

-Selective Transport Proteins

-Passive Transport

-Diffusion

-Facilitated Diffusion

-Chemical/Concentration Gradient

-Electrochemical Gradient

-Equilibrium

-Diffusion Rate

Simulation Link: https://phet.colorado.edu/sims/cheerpj/membrane-channels/latest/membrane-channels.html?simulation=membrane-channels

ELABORATE

To deepen students understanding of diffusion, electrochemical gradients, and the role of polymers in biomedical applications, students will design and carry out a full factorial experiment to investigate how calcium ions are released from alginate hydrogels. The teacher will first explain how sodium alginate undergoes ionic crosslinking with calcium chloride to form a gel, and how free (loose) and bound calcium ions are released from the hydrogel matrix by following the concentration/chemical gradient.

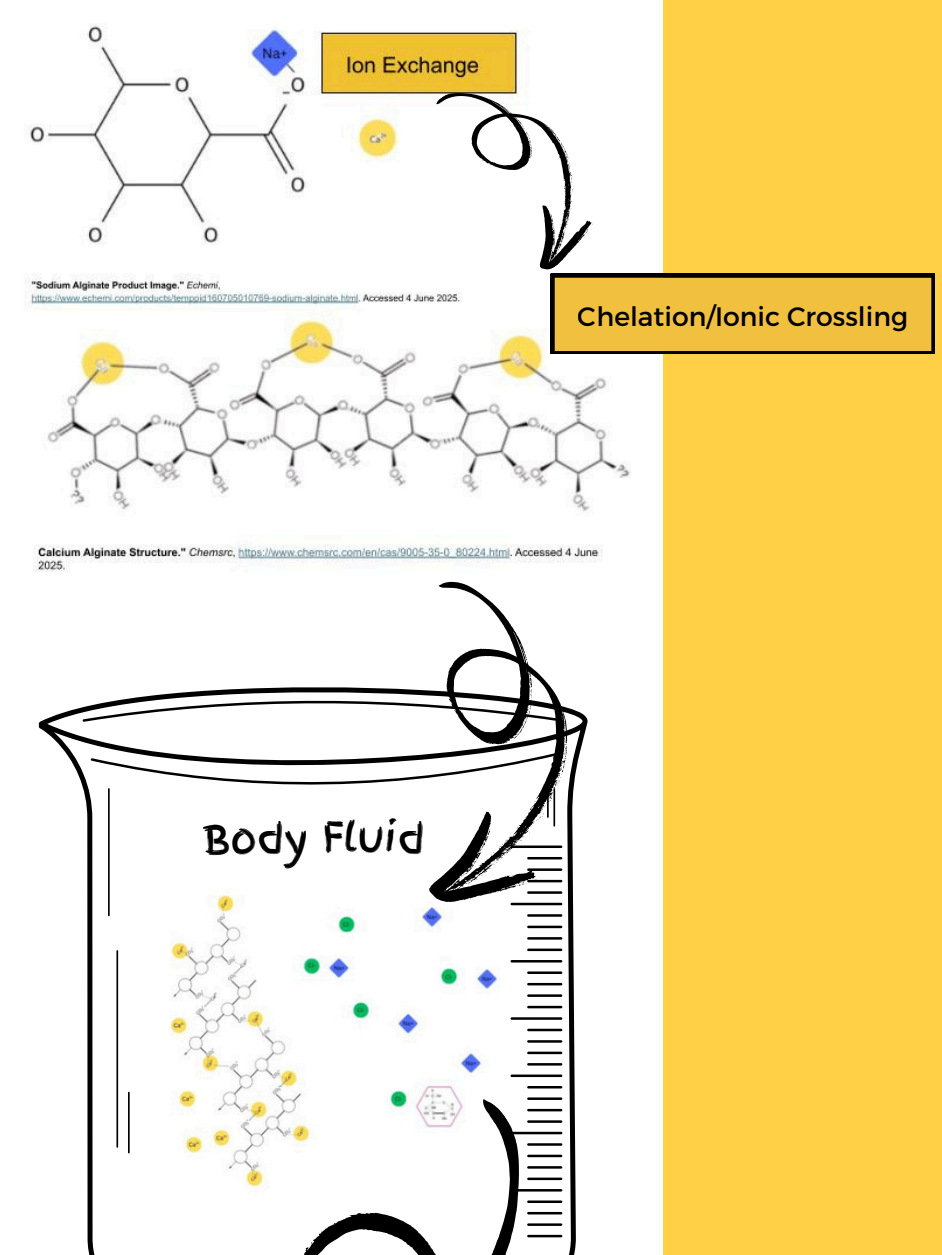

Students will explore how changing two variables—sodium alginate concentration and calcium chloride concentration in a external solution of salt/glucose solution simulating body fluids affects:

- The rate of calcium ion diffusion out of the hydrogel,
- The stiffness and structural integrity of the hydrogel over time.

Using a 3^2 full factorial design, students will set up nine experimental combinations and track calcium release by examining changes in the hydrogel's mass and **Total Dissolved Solids** in surrounding hydrogel. Students will also assess hydrogel stiffness by timing how fast the hydrogel slides down an inclined plane every 5 minutes.

This hands-on investigation allows students to apply scientific concepts in a real-world context, modeling how controlled drug delivery systems are tested in biomedical engineering. Through this activity, students will discover how multiple variables interact and how experimental design is used in industry to optimize product performance and reduce testing time.

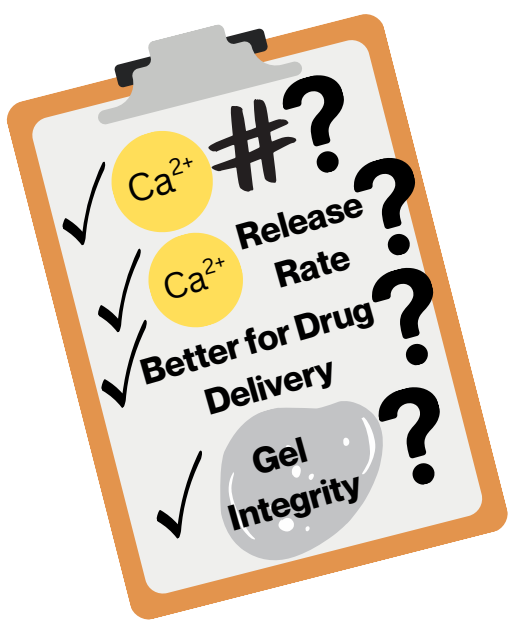

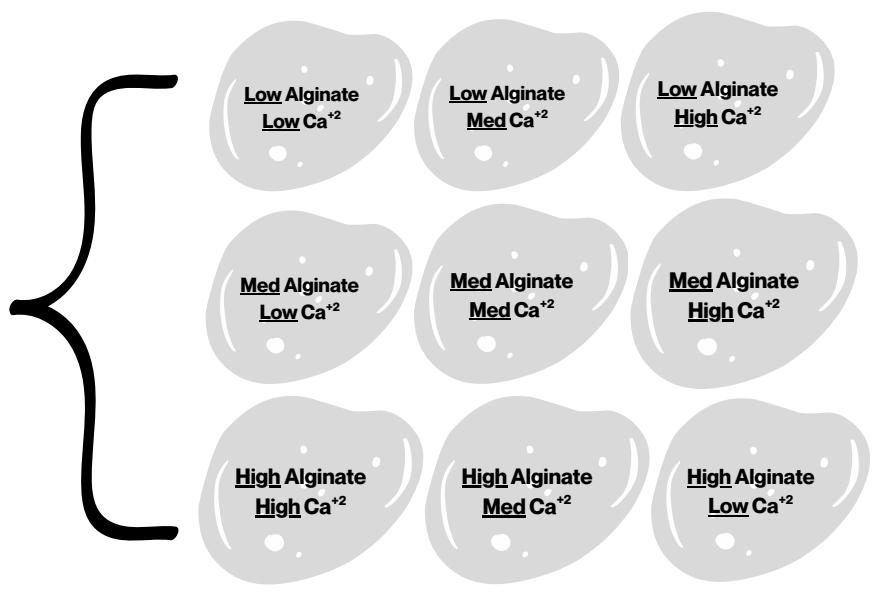

SCHOOL OF POLYMER SCIENCE AND ENGINEERING SOUTHERN MISS

EVALUATE

Each student will be given a different **scenario card**. There are 8 possible scenario cards. Example below. Students will apply the idea of diffusion, facilitated diffusion, the electrochemical gradient, and ion exchange to determine the movement of each of the following ions Ca+2, Cl-, Na+, and K+.

Students can write-out, illustrate, and/or verbally express their conclusion.

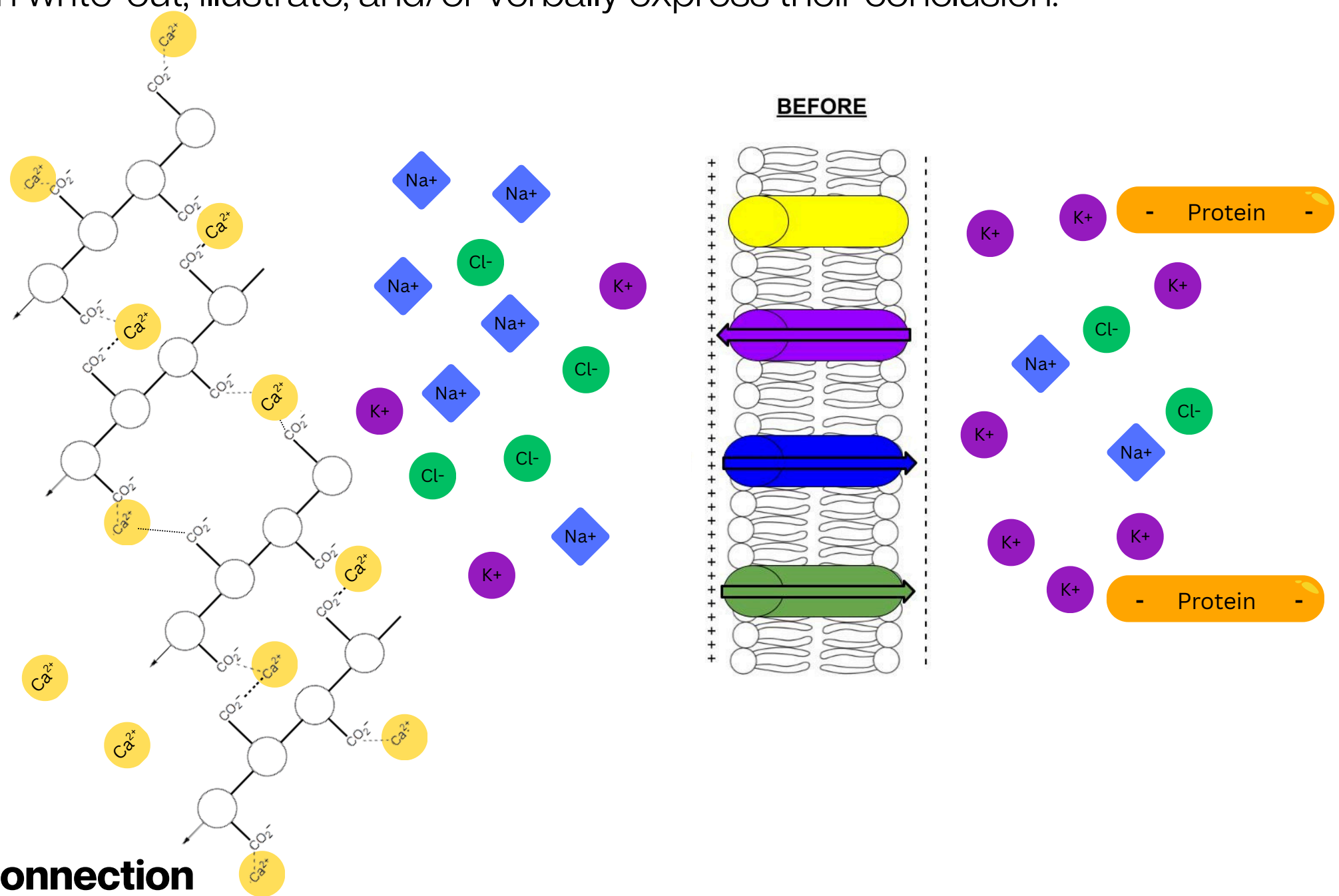

Make the Connection

What would happen if this system was put into a living organism?

EXTENSION & REMEDIATION

The students will answer concept specific questions via an exit ticket, the next day's bell-ringer, or homework quiz.

This assessment will help the teacher evaluate student knowledge and understanding of related concepts

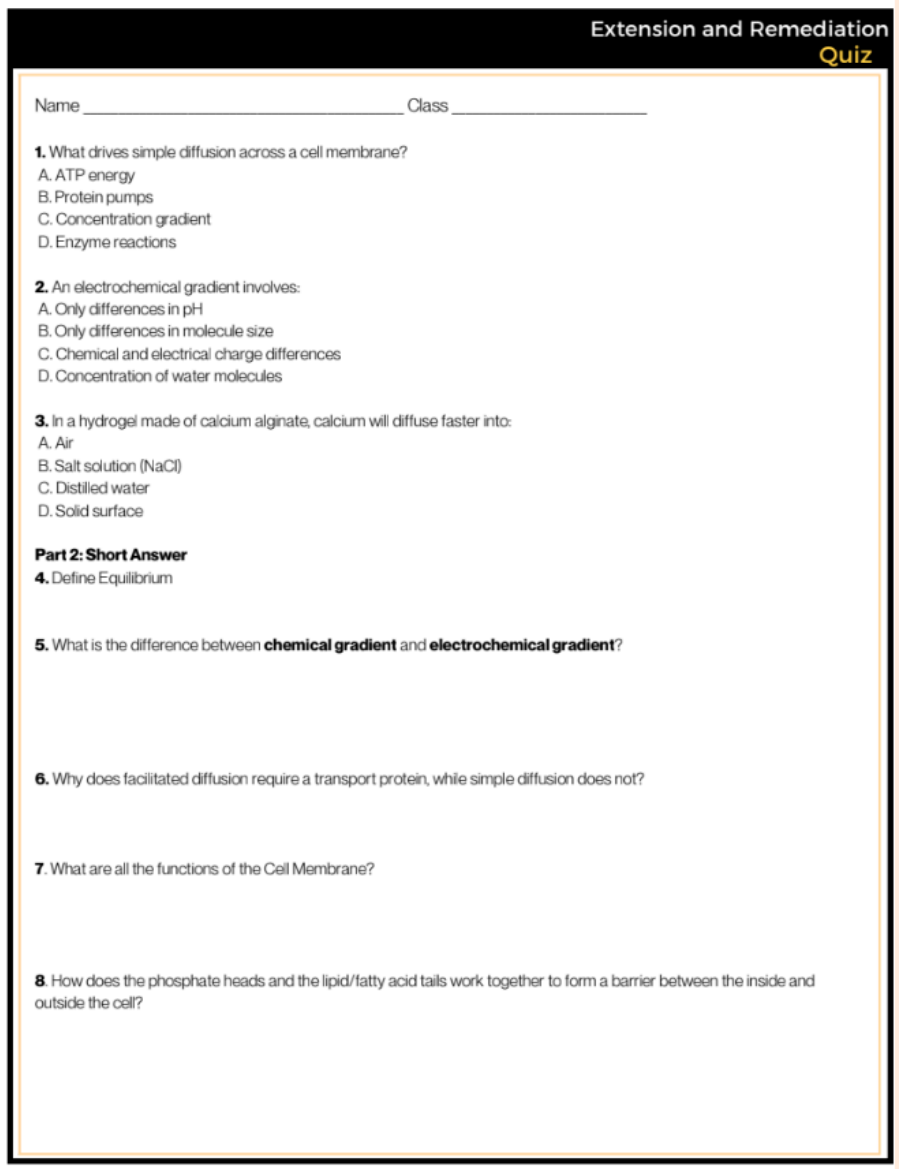

Extension and Remediation
Quiz

Name ______________________ Class ________________

1. What drives simple diffusion across a cell membrane?
A. ATP energy
B. Protein pumps
C. Concentration gradient
D. Enzyme reactions

2. An electrochemical gradient involves:
A. Only differences in pH
B. Only differences in molecule size
C. Chemical and electrical charge differences
D. Concentration of water molecules

3. In a hydrogel made of calcium alginate, calcium will diffuse faster into:
A. Air
B. Salt solution (NaCl)
C. Distilled water
D. Solid surface

Part 2: Short Answer

4. Define Equilibrium

5. What is the difference between **chemical gradient** and **electrochemical gradient**?

6. Why does facilitated diffusion require a transport protein, while simple diffusion does not?

7. What are all the functions of the Cell Membrane?

8. How does the phosphate heads and the lipid/fatty acid tails work together to form a barrier between the inside and outside the cell?

The **Cell Membrane**

- Surrounds the Cell
- Creates a barrier between inside and outside the cell.
- Controls what goes in and out of the cell.

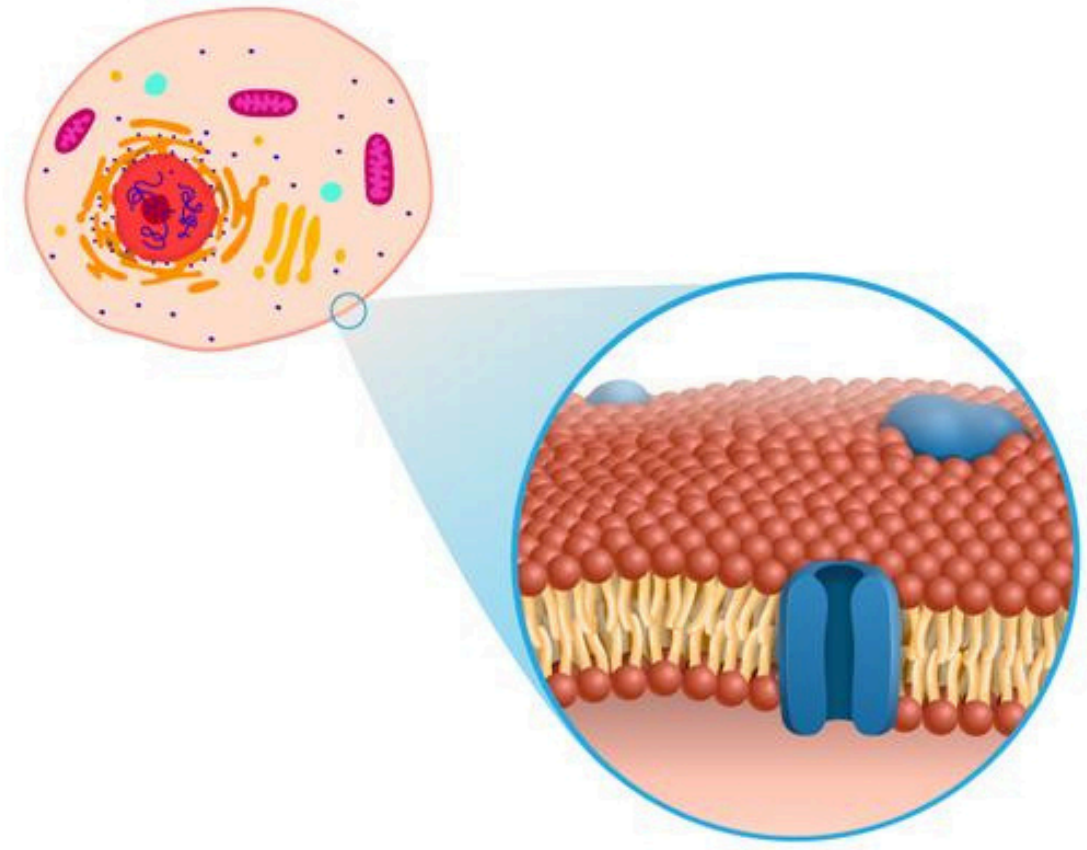

Cell Membrane Anatomy

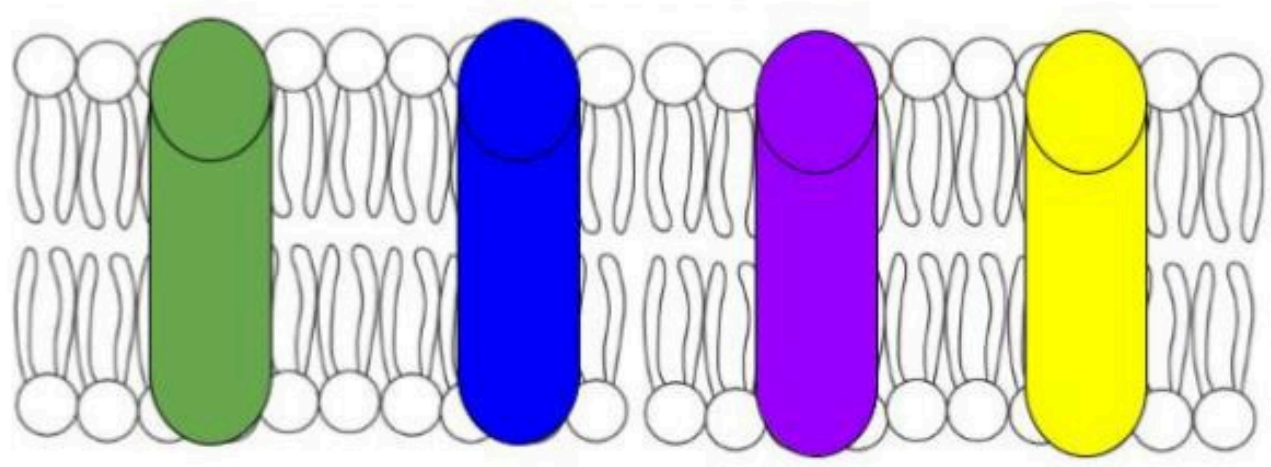

**Phospholipid Bilayer (TWO Layers)**	**Embedded Transport Proteins**
**Phosphate Head** Hydrophilic Attrachs H_20 — **Lipid/Fatty Acid Tails** Hydrophobic Repels H_20	Within the Cell Membrane/Phospholipid Bilayer are **Transport Proteins,** they help materials cross in and out of the cell.

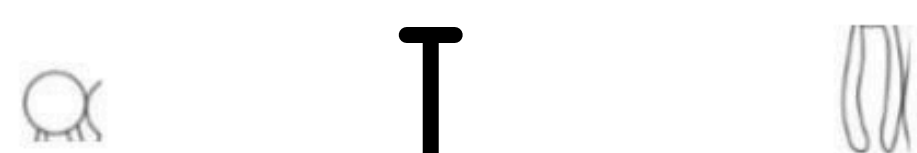

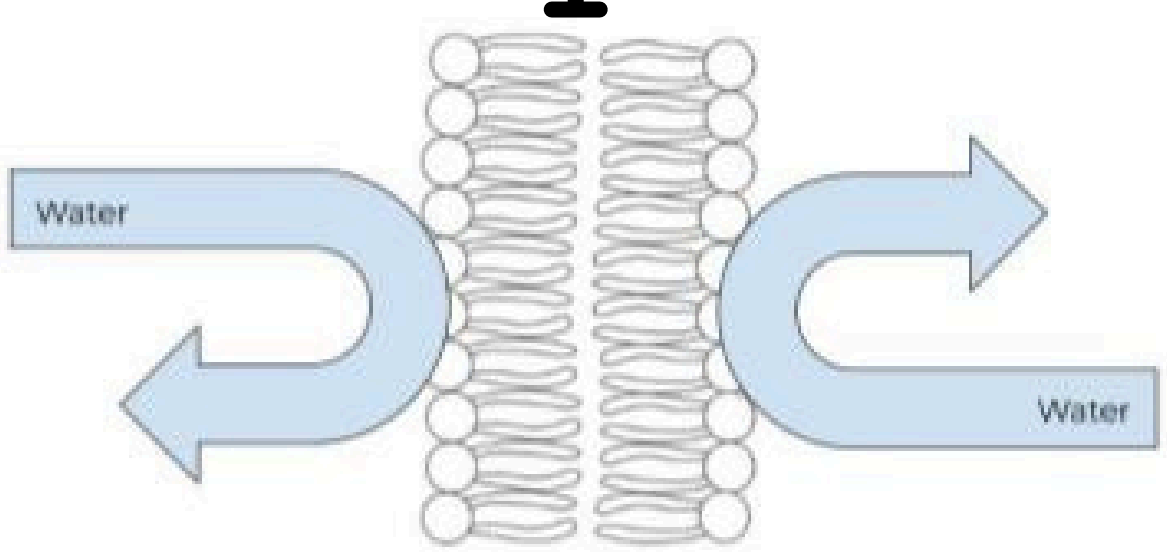

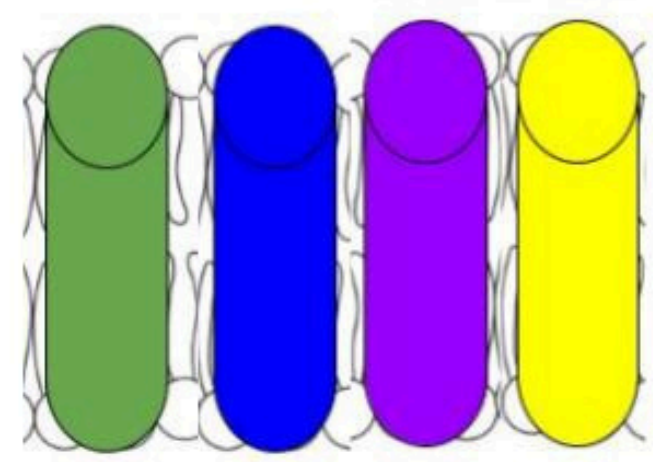

The combined forces of the Phosphate Head and the Lipid Tails allows the Cell membrane to act as a barrier between inside the cell and outside the cell.

The Inside and Outside of the cell's membrane

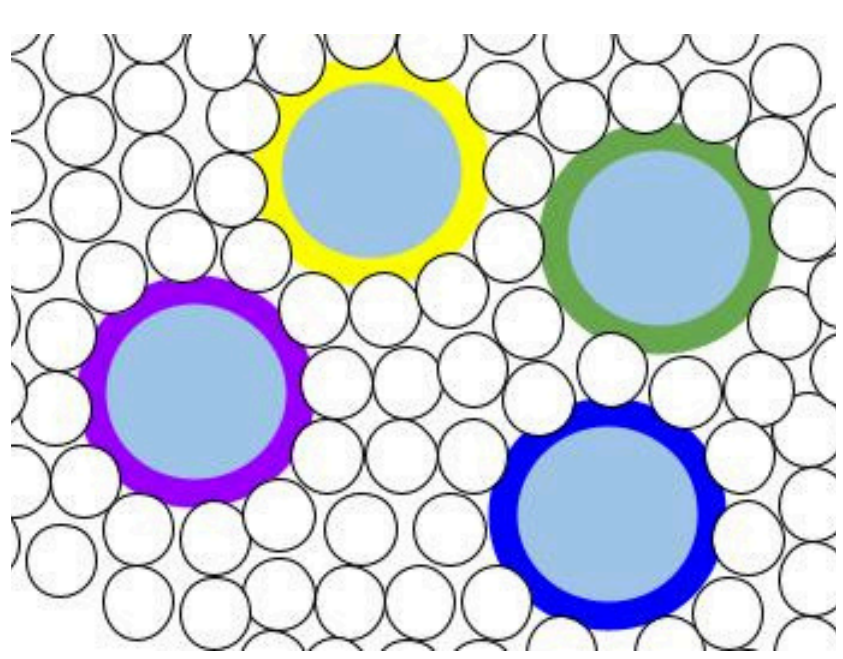

Is the Cell Membrane completely solid??

Could a molecule flow freely through the **Phospholipid Bilayer**?

But what would be the requirement?

Can any size material pass through the **Phospholipid Bilayer**?

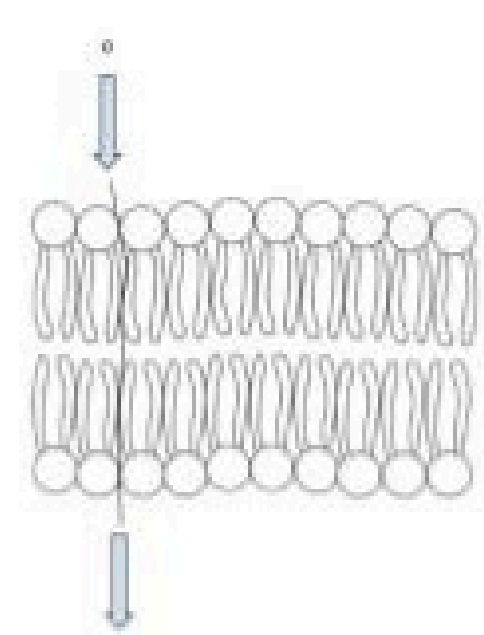

Concept: **Simple Diffusion**: Small materials move from High to Low concentration through the Phospholipid Bilayer, directly through the cell membare

Do material just move in and out of the cell with no direction?
Materials move with the Concentration Gradient, from High to Low

Open up the simulation at Phet using this link:
https://phet.colorado.edu/sims/cheerpj/membrane-channels/latest/membrane-channels.html?simulation=membrane-channels

Cell Transport

Troubleshooting: Set the Sim Speed to Fast and if the simulation freezes click refresh,

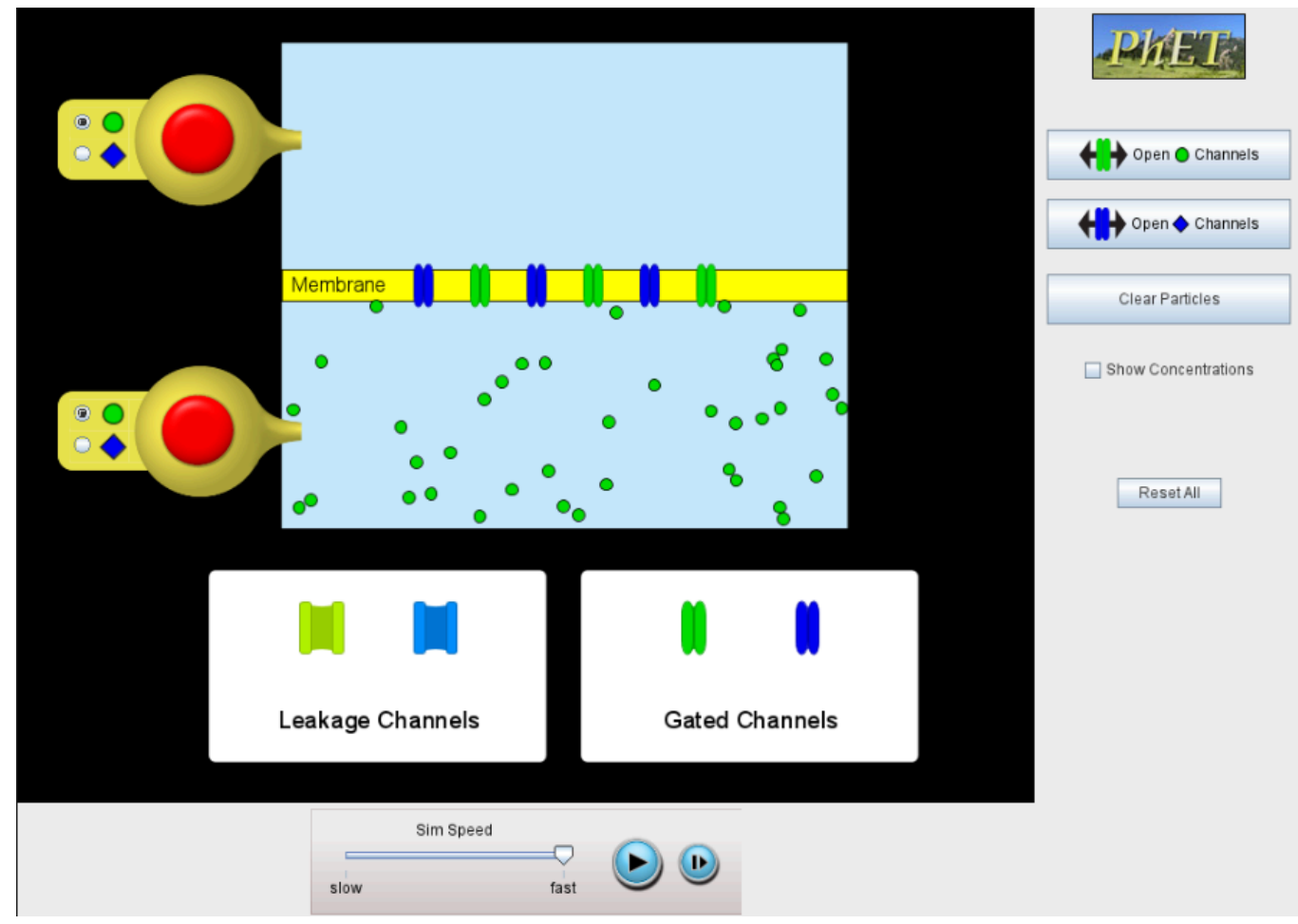

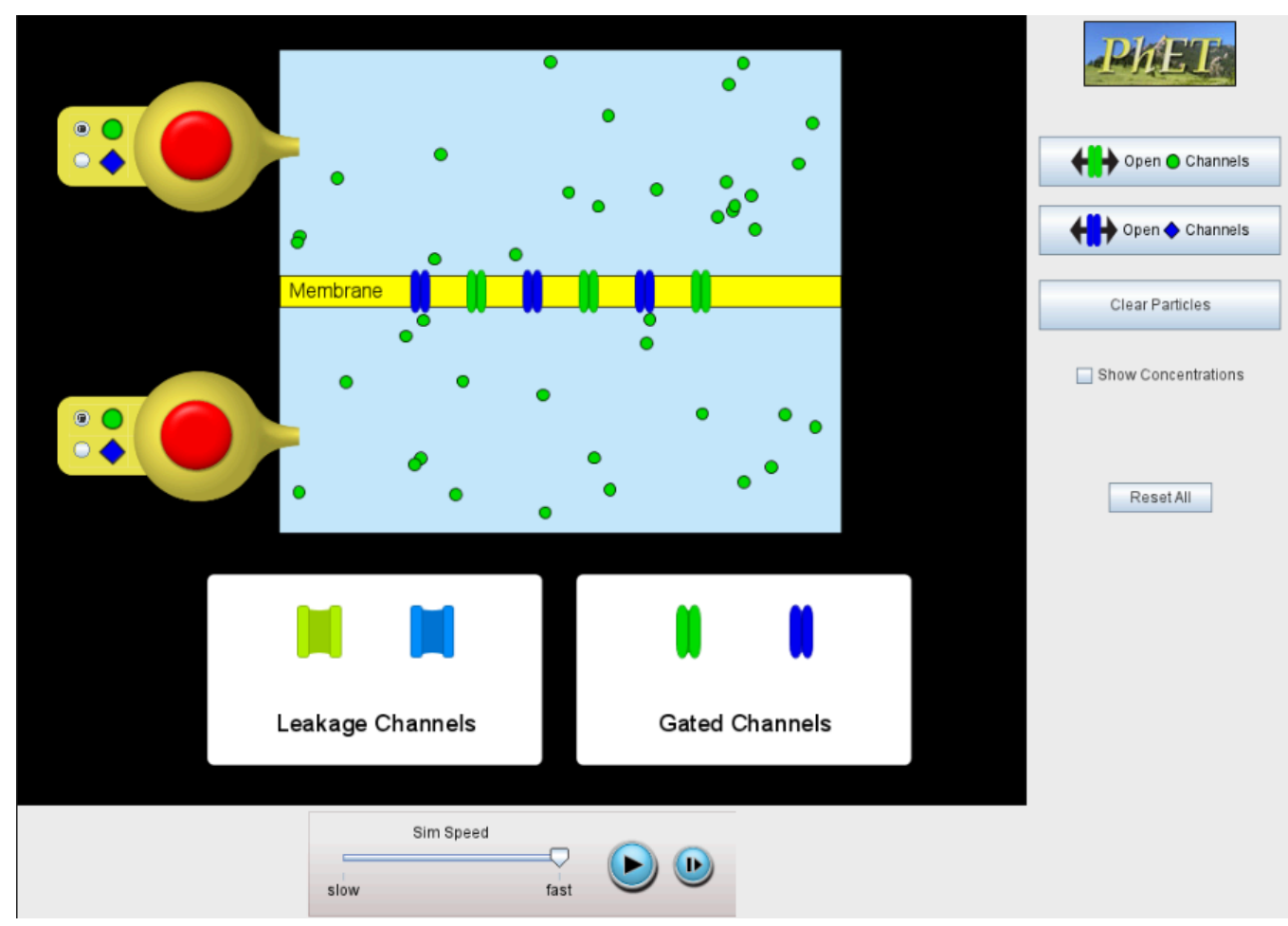

Directions

Run 1

1- Move 3 BLUE gate proteins and move 3 GREEN gate proteins. Keep the gate proteins closed.

2- Add 40 green Cl- ions to one side of the membrane
Complete the table at 0 Secs

3- Open up the gates

4- Allow the Sim to run for ~20 secs

5- At 20 secs close the gates and pause the sim
Question: In what direction or how did the Cl- ions move?
Complete the table at 20 Secs

6- Unpause the sim and open the gates

7- Let the sim run for an additional ~40 secs

8- At 40 secs (total 1 min) close the gates and pause the sim
Complete the table at 60 secs/1 min
Question: Looking at the table, what do you notice when you compare the # of Cl- ions on one side to the other side of the membrane?

**Time**	**Start: 0 secs**	**20 secs**	**60 secs/ 1 min**
# of Cl- Ions on Top			
# of Cl- Ions on Bottom			

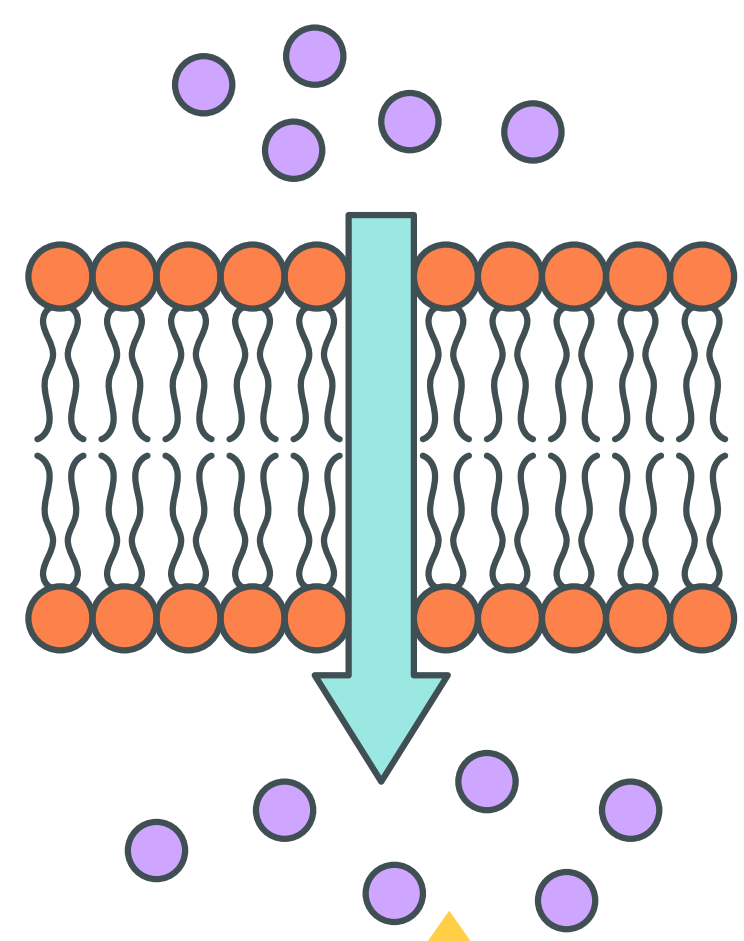

Click Reset All or Refresh Page

Concept: Diffusion-Materials naturally move from **high to low concentration** until reaching **equilibrium.**

What if the material is TOO Big to fit directly through the Phospholipid Bilayer?
Facilitated Diffusion

Directions
Run 2

1- Add 20 green Cl- ions to one side of the membrane
Question: What do you observe about the Cl- Ions?

2- Add 3 Cl- gate proteins to the membrane and open the gates.
Question: What do you observe about the Cl- ions?
Question: In what direction did the Cl- ions move?

3- Check the show concentration box
Question: After the simulation ran for ~20 secs, what do you notice about the concentrations on either side of the membrane?

Troubleshooting: Set the Sim Speed to Fast and if the simulation freezes click refresh,

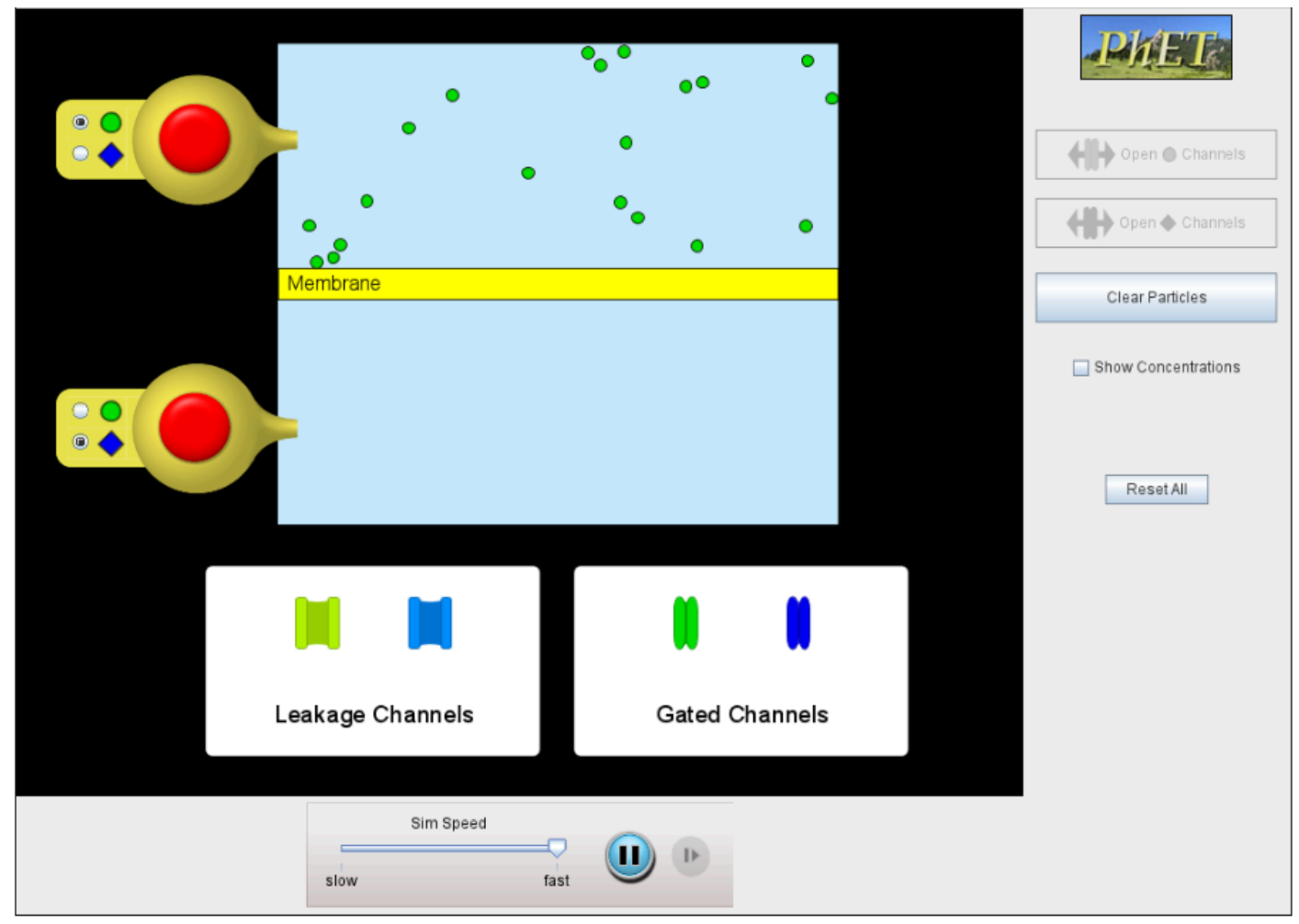

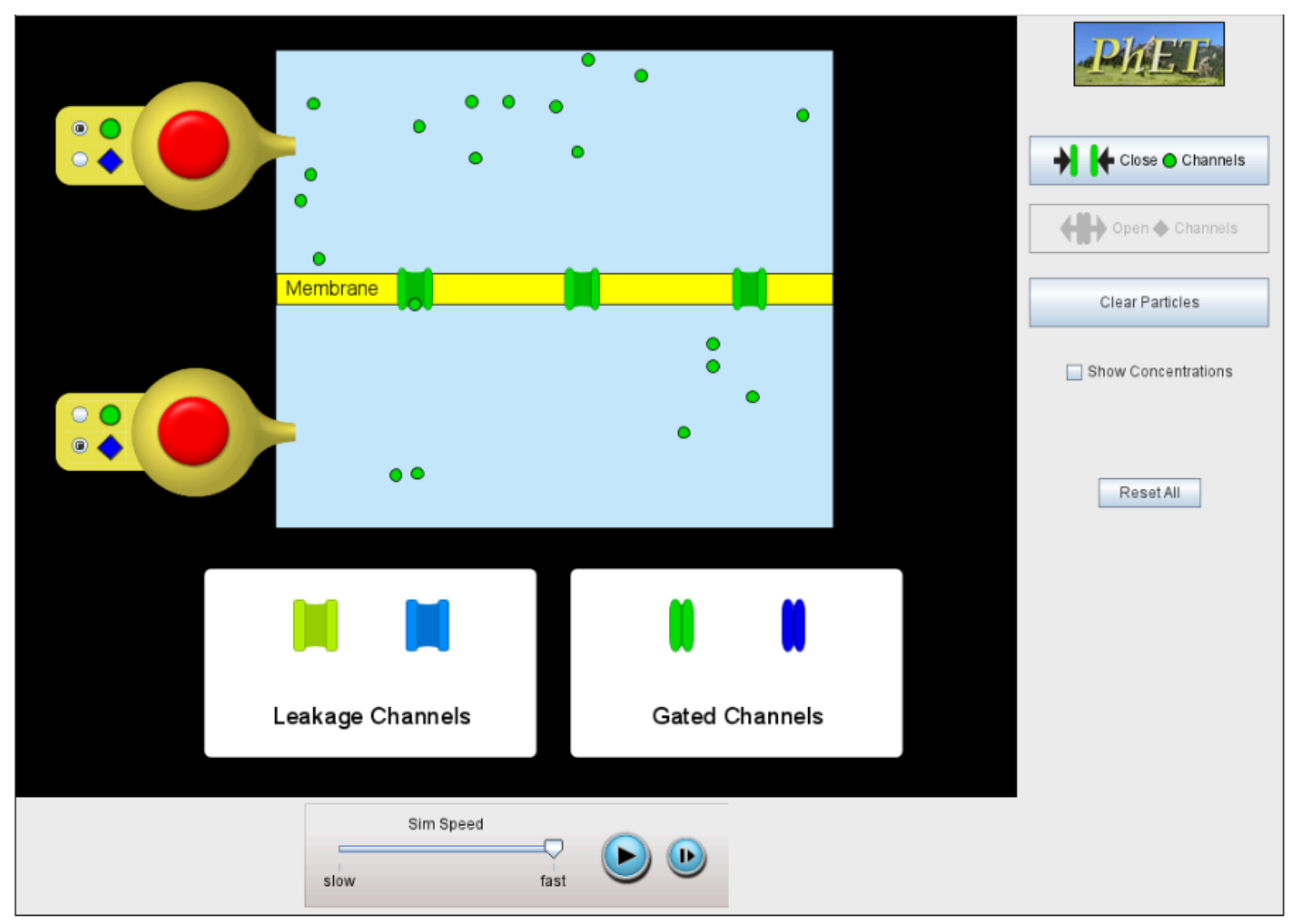

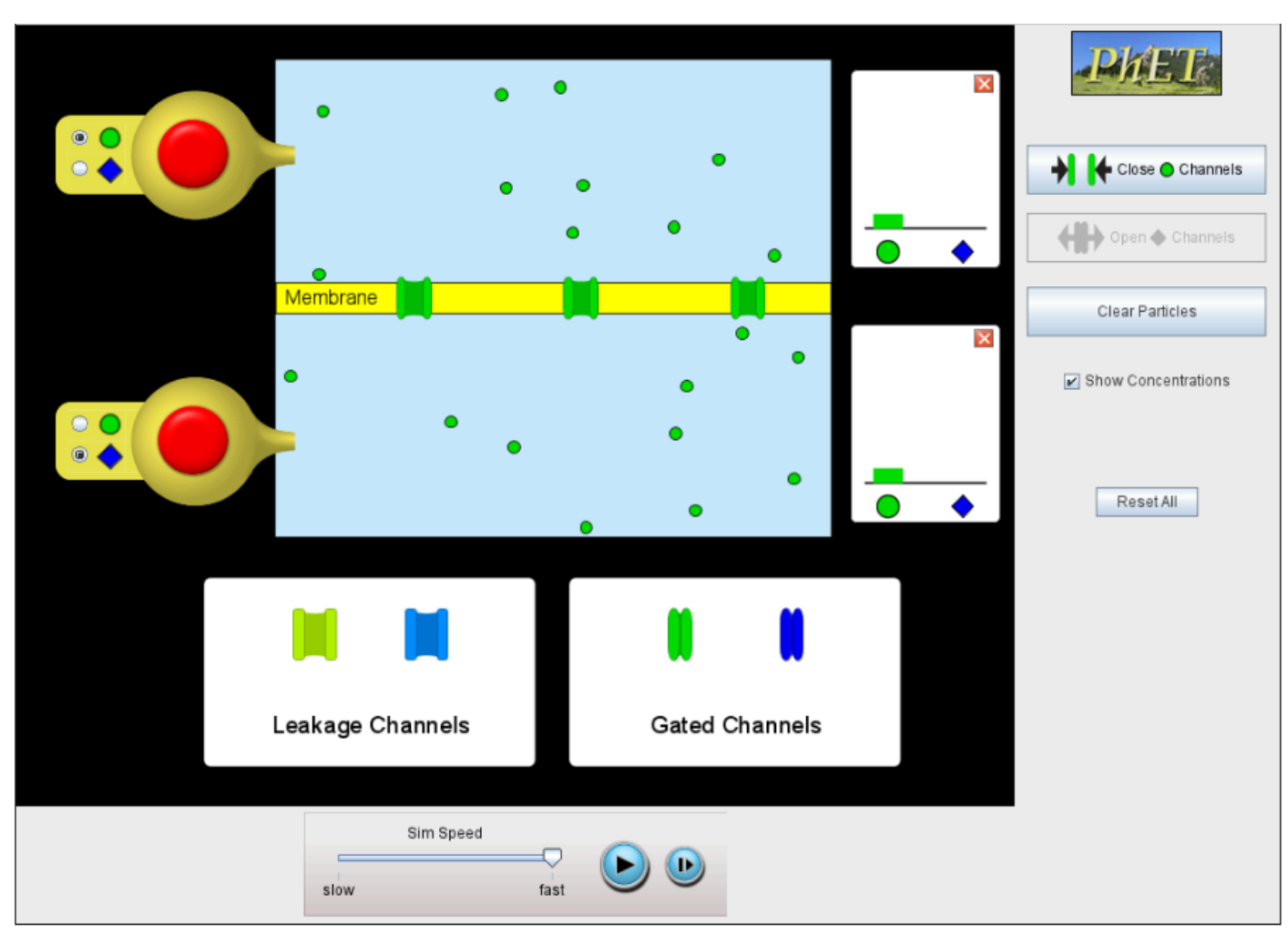

Concept:

Facilitated Diffusion- <u>Large</u> materials (that are too big to pass through the membrane) use a <u>transport protein</u> to move from <u>High to Low</u> concentration.

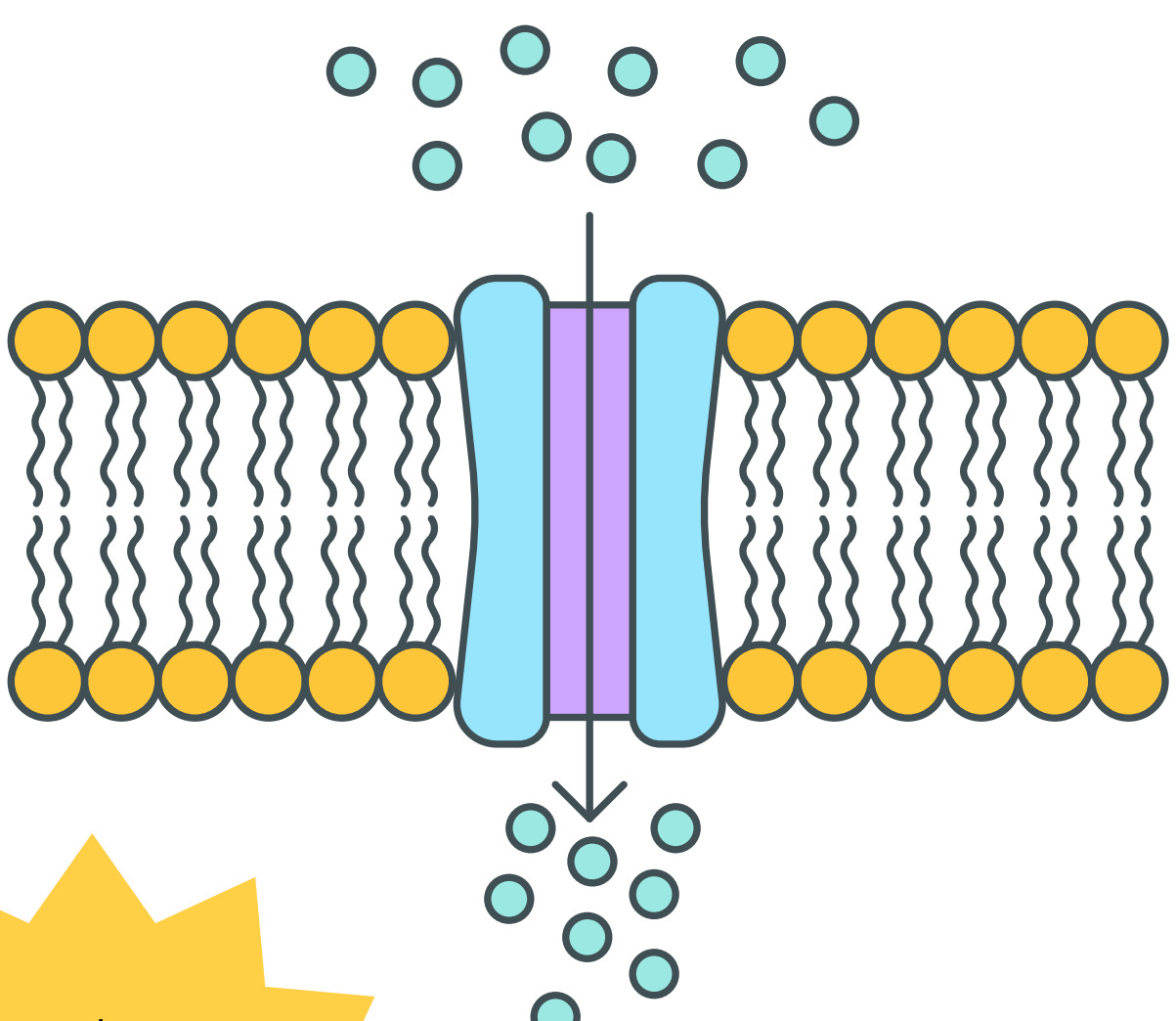

Click Reset All or Refresh Page

Can any molecule go through a gate protein?
Transport/Gate Proteins are Specific

Directions

Run 3

1- Move 2 blue gate proteins and move 2 green gate proteins. Keep the gate proteins closed

2- Add 30 green Cl- ions on one side of the membrane

3- Open up the blue gate proteins
Question: What do you observe?

4- Open up the Green gate proteins.
Question: What do you observe?

5- Add blue Na+ ions on one side of the cell membrane
Question: What do you observe?
Question: So what can you conclude about gate proteins?

Troubleshooting: Set the Sim Speed to Fast and if the simulation freezes click refresh,

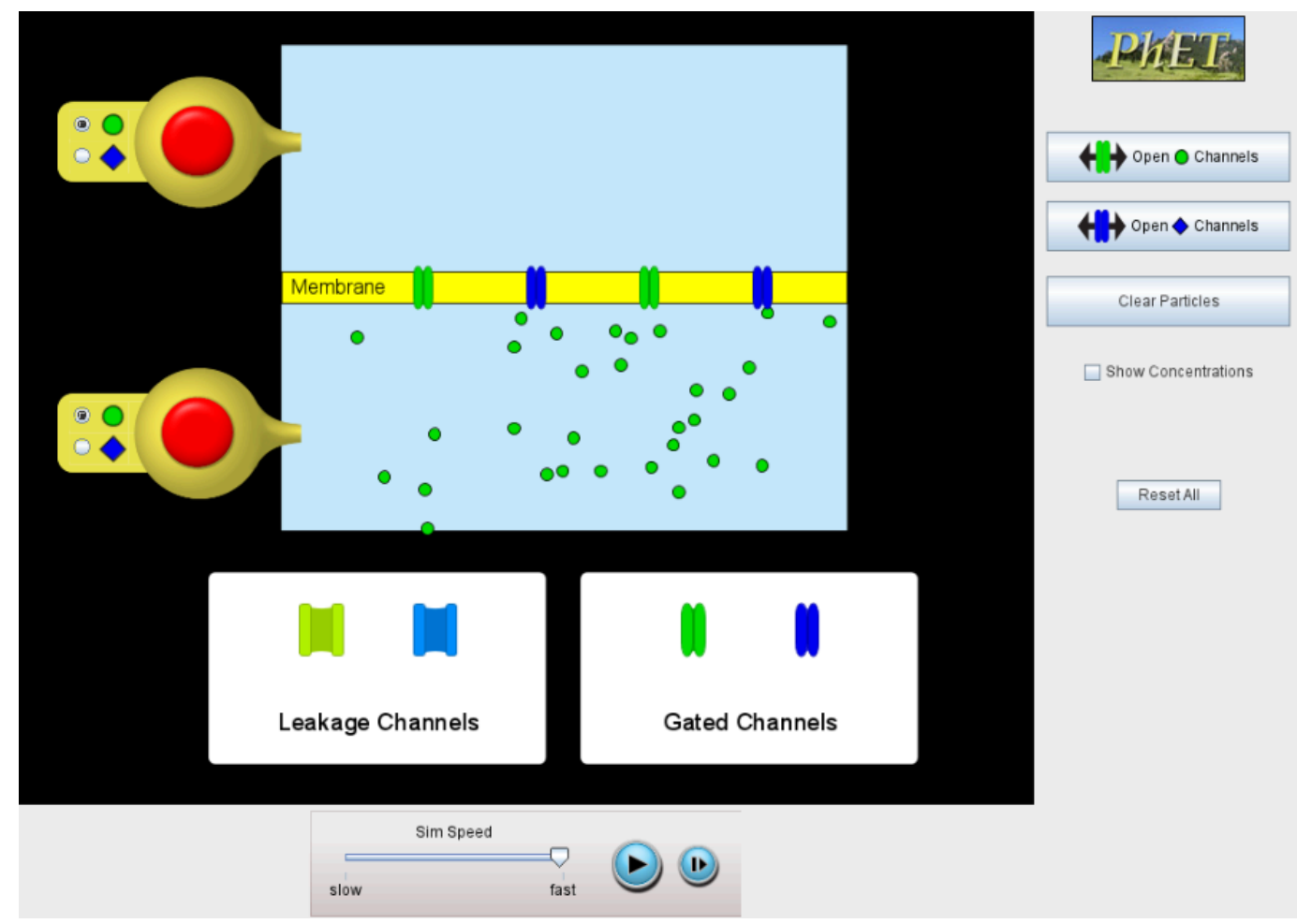

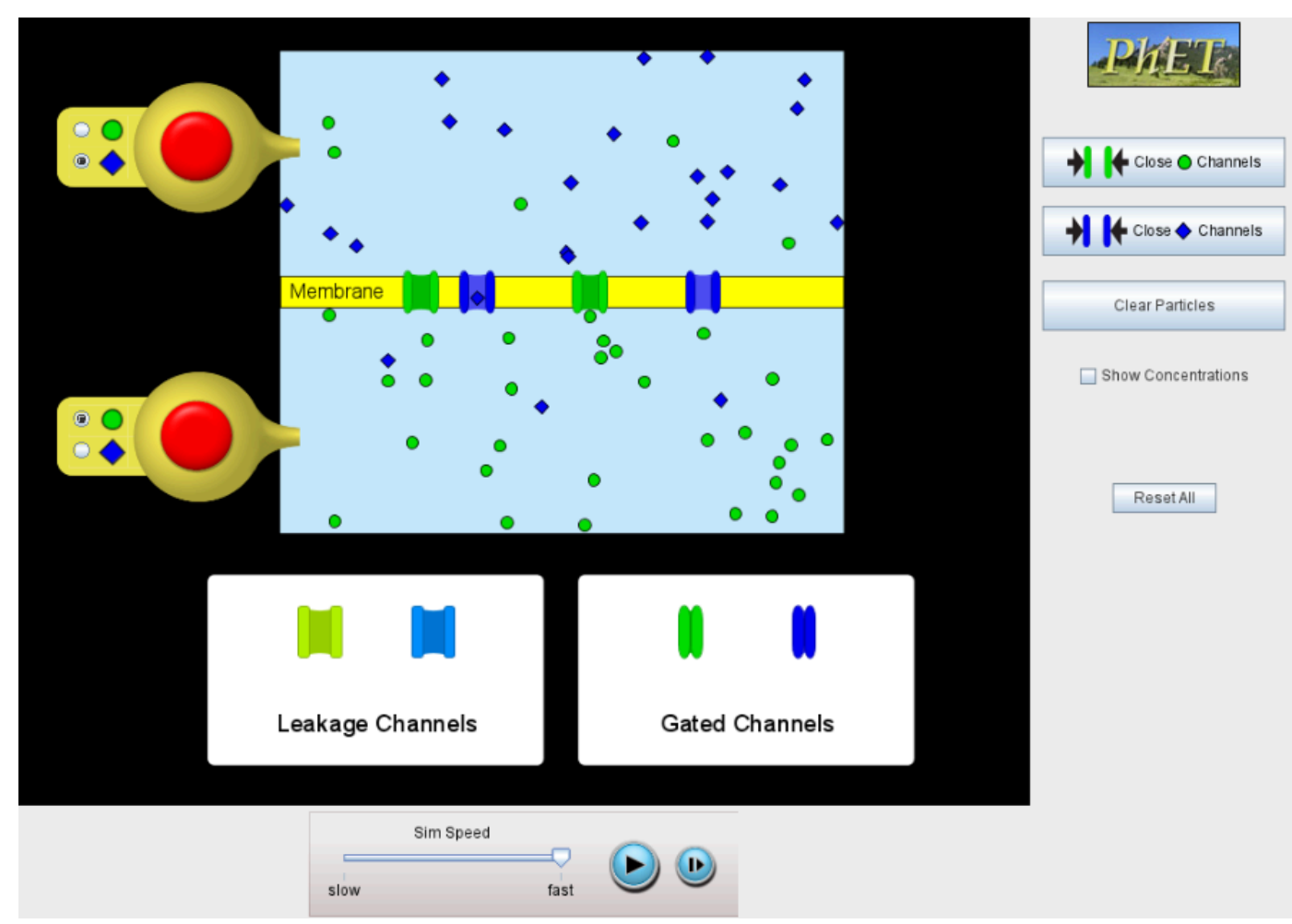

Concept: There are specific transport/gate proteins for specific materials to move in and out of the cell.

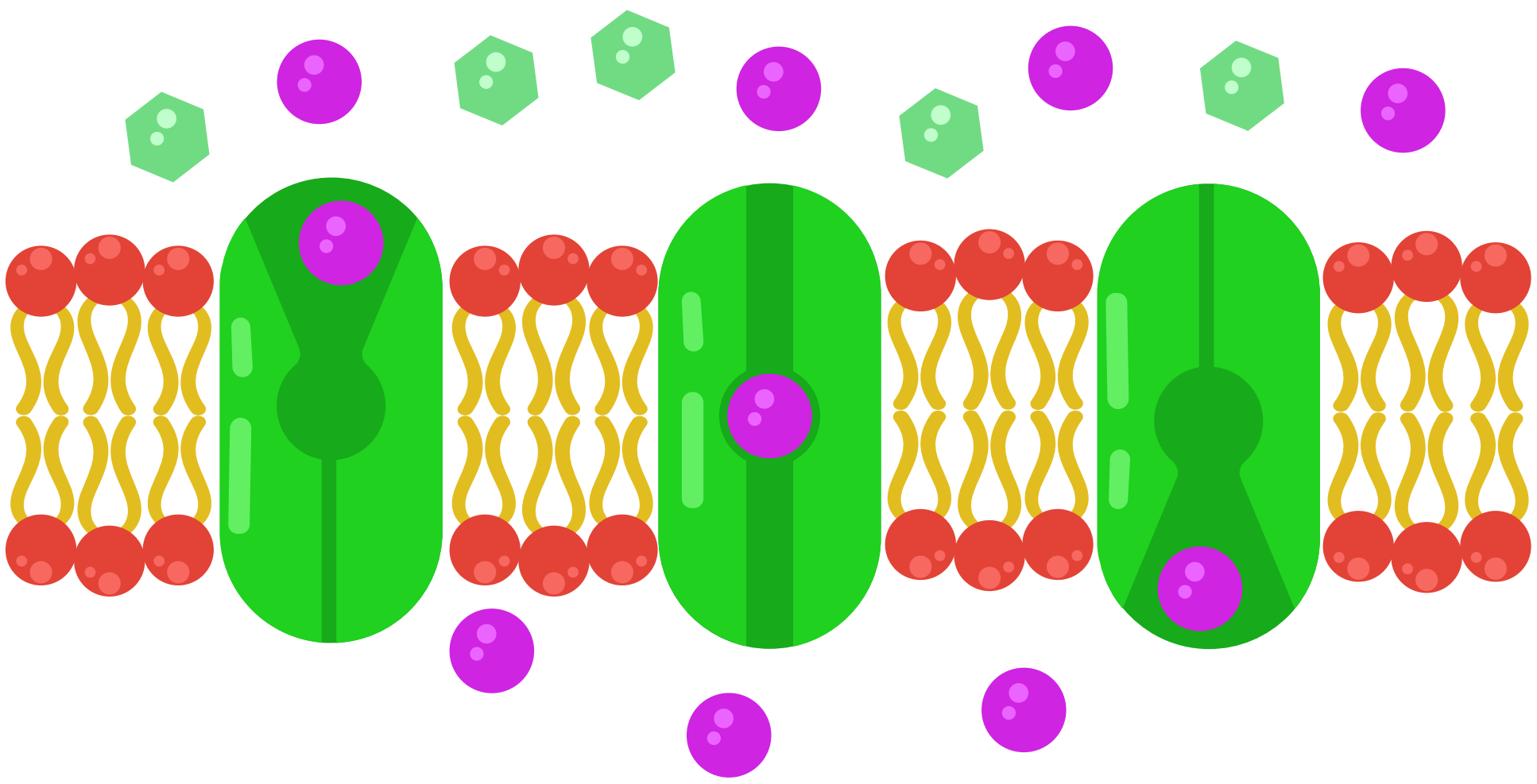

Click Reset All or Refresh Page

Why do materials naturally move? Is there a driving force?
Concentration /Chemical Gradient

Troubleshooting: Set the Sim Speed to Fast and If the simulation freezes click refresh,

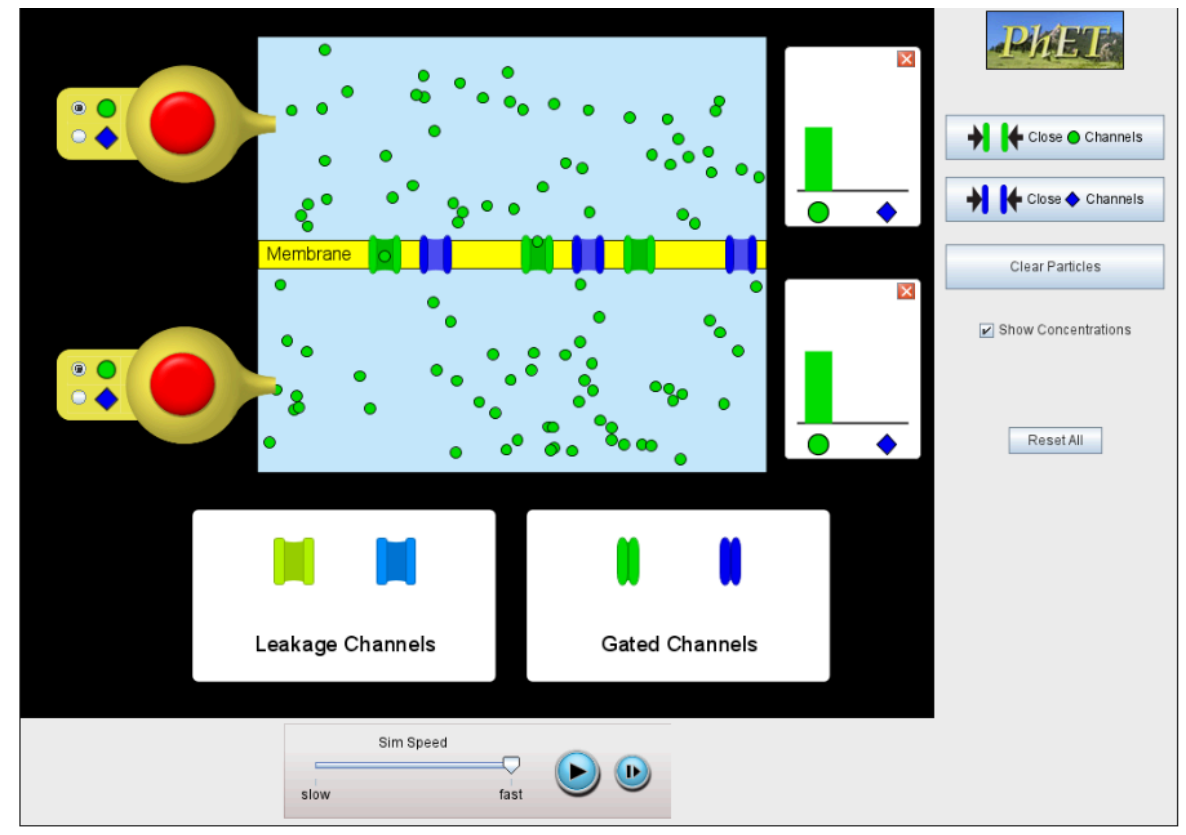

Directions

Run 4

1- Move 3 Sodium, Na+ gate proteins and move 3 Chloride, Cl- gate proteins. Keep the gate proteins closed

2- Add 40 Cl- ions to both sides of the membrane (40 on top and 40 on bottom)

3- Check the show concentration box

4- Open up the gates

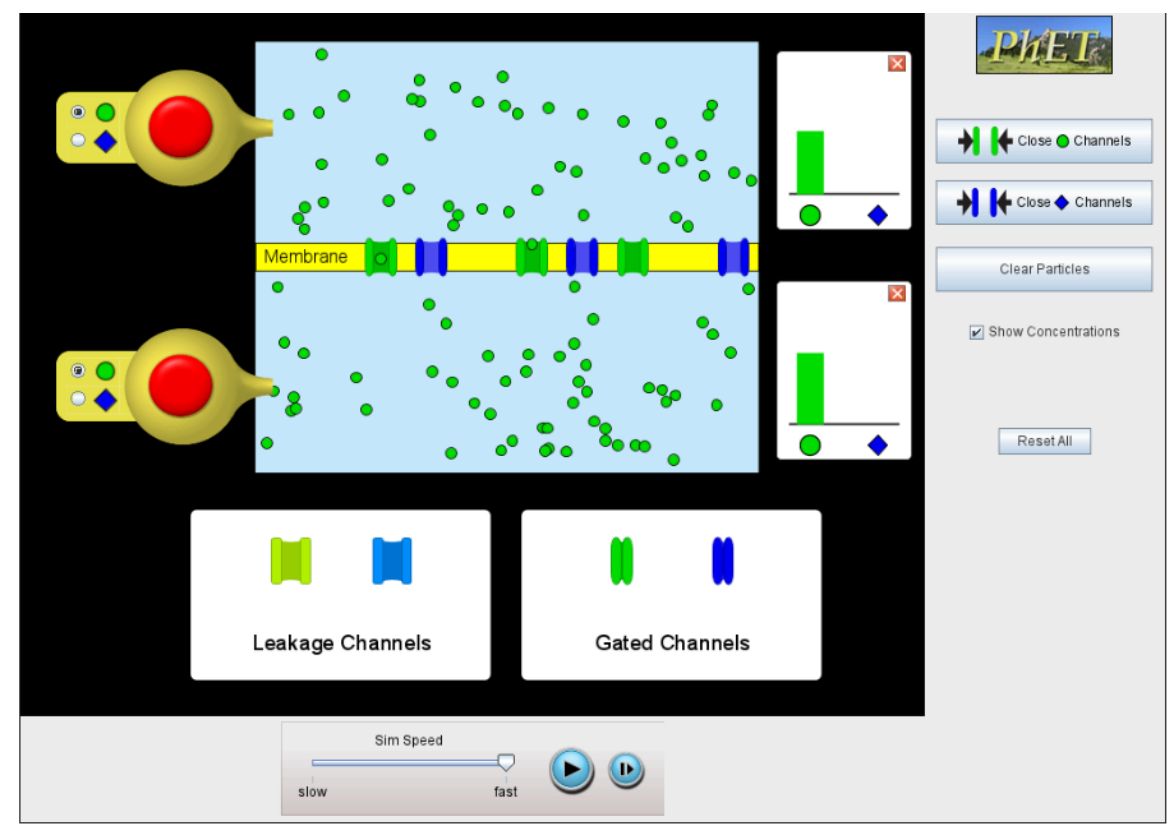

5- Allow the sim to run for ~30 secs

6- Pause the Sim

Question: When you started the sim there are 40 Cl- ions on both sides of the membrane, that means you started the sim at ______.

Question: During the sim was there a a big shift in concentrations of Cl- ions to either side of the membrane? Y or N

7-Unpause the sim, close the gates, and add 20 more Cl- ions to ONE side of the membrane

Question: Now the concentrations on both sides of the membrane are _____.

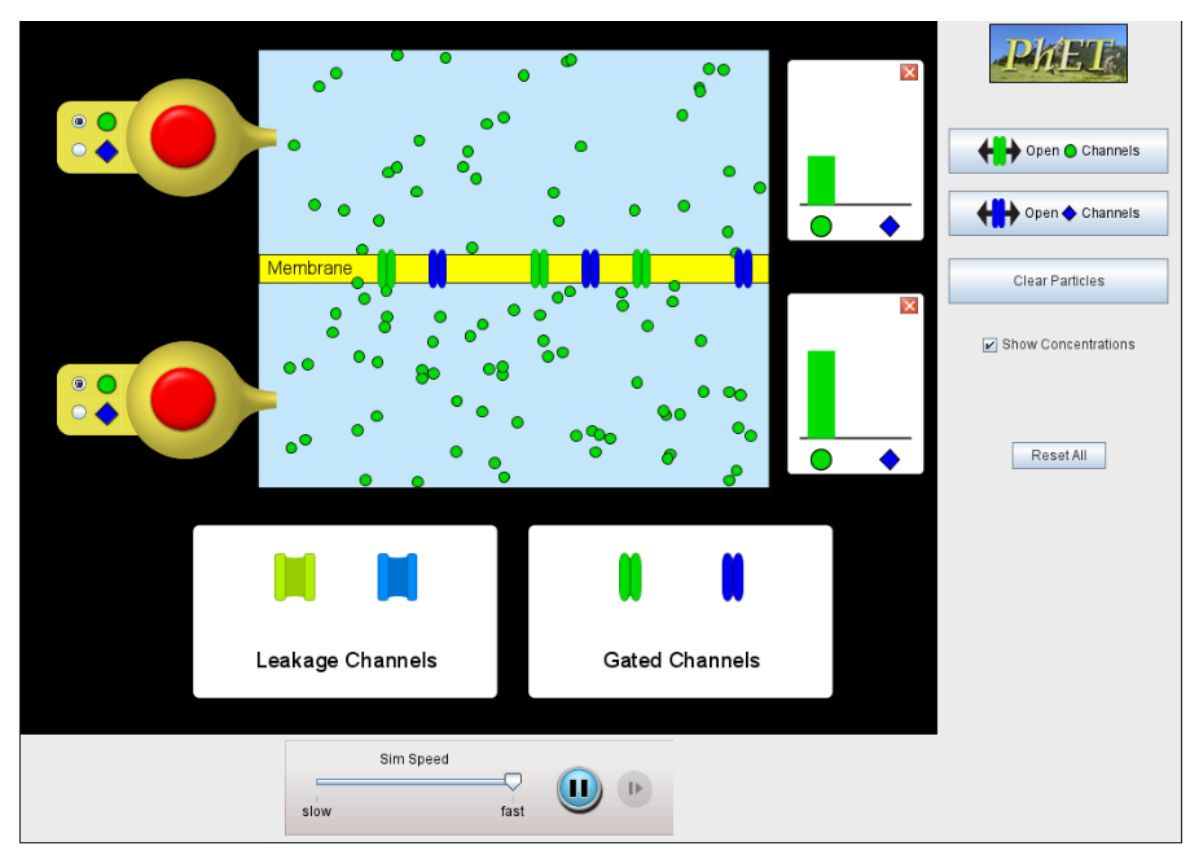

8- Open the gates and allow the sim to run for ~20 secs.

9- Monitor the concentration graph through-out the 20 secs

10- At 20 secs pause the sim

Question: What do you notice about the concentrations on both sides of the membrane?

Question: How did this happen? Where did the Cl- ions go or how did they move?

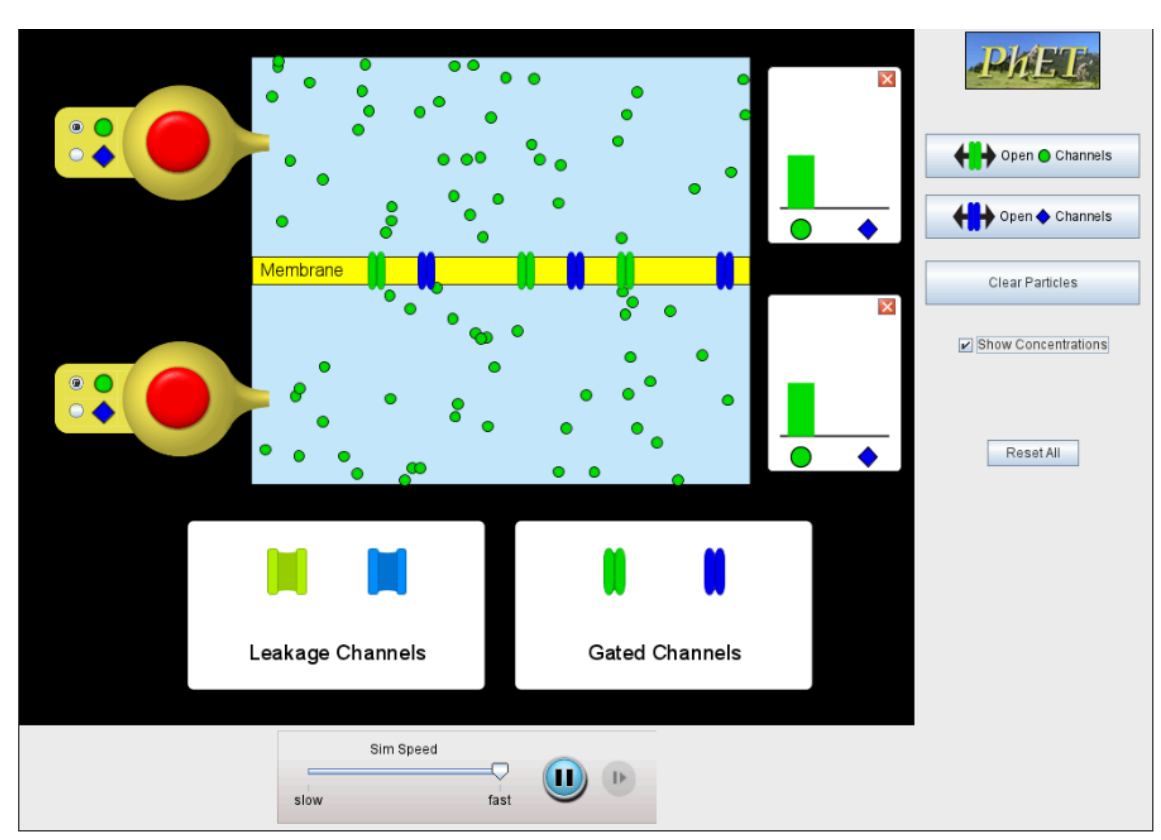

Concept: In order for diffusion to occur, there must be a difference in concentrations inside and outside the cell which is called a **Concentration Gradient.**

Close the Phet Tab

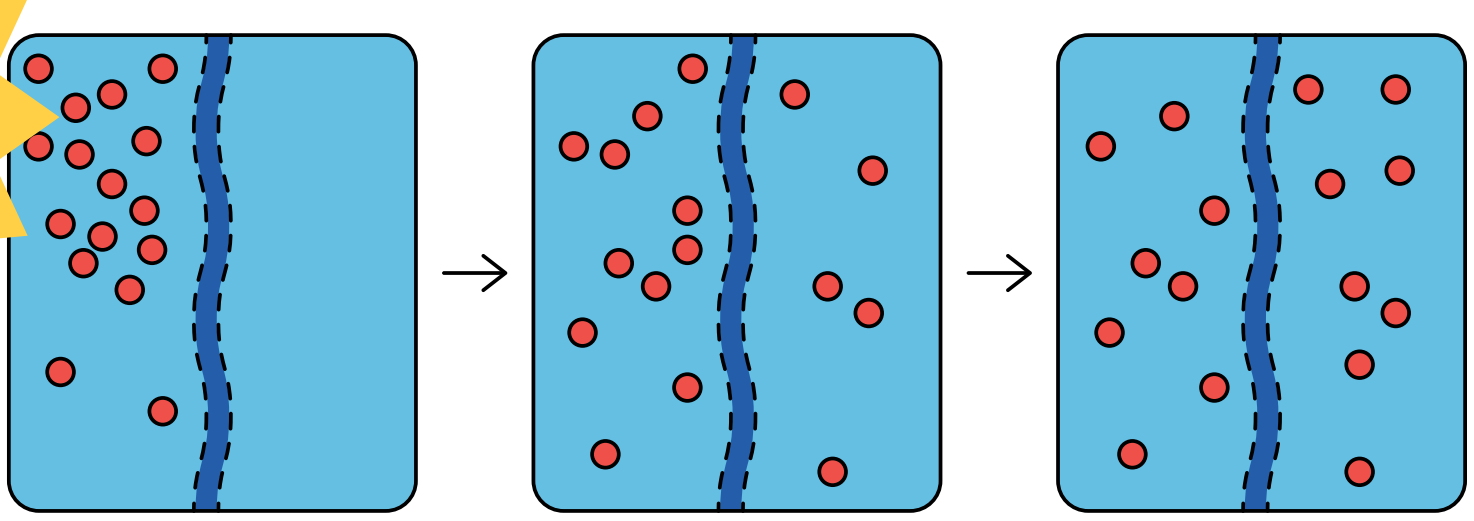

Is the only driving force for Diffusion the Concentration/Chemical Gradient?
Electrochemical Gradient

Directions

Run 5:

Question: Na+ ions give off what charge?

Question: Cl- ions give off what charge?

Question: When you put 2 positives together they?

Question: When you put 2 negatives together they?

Question: When you put a negative and positive together, they?

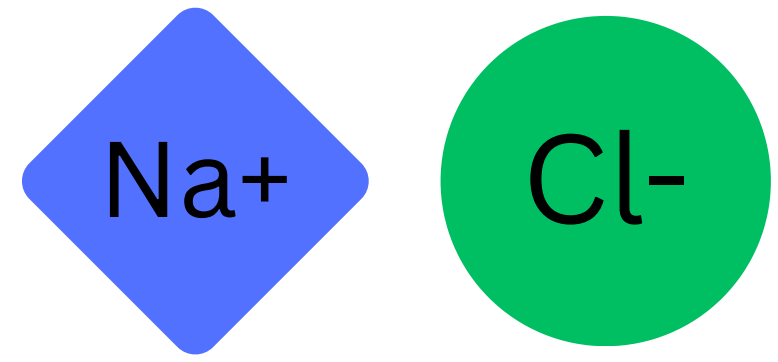

Use Graphics to complete the table.

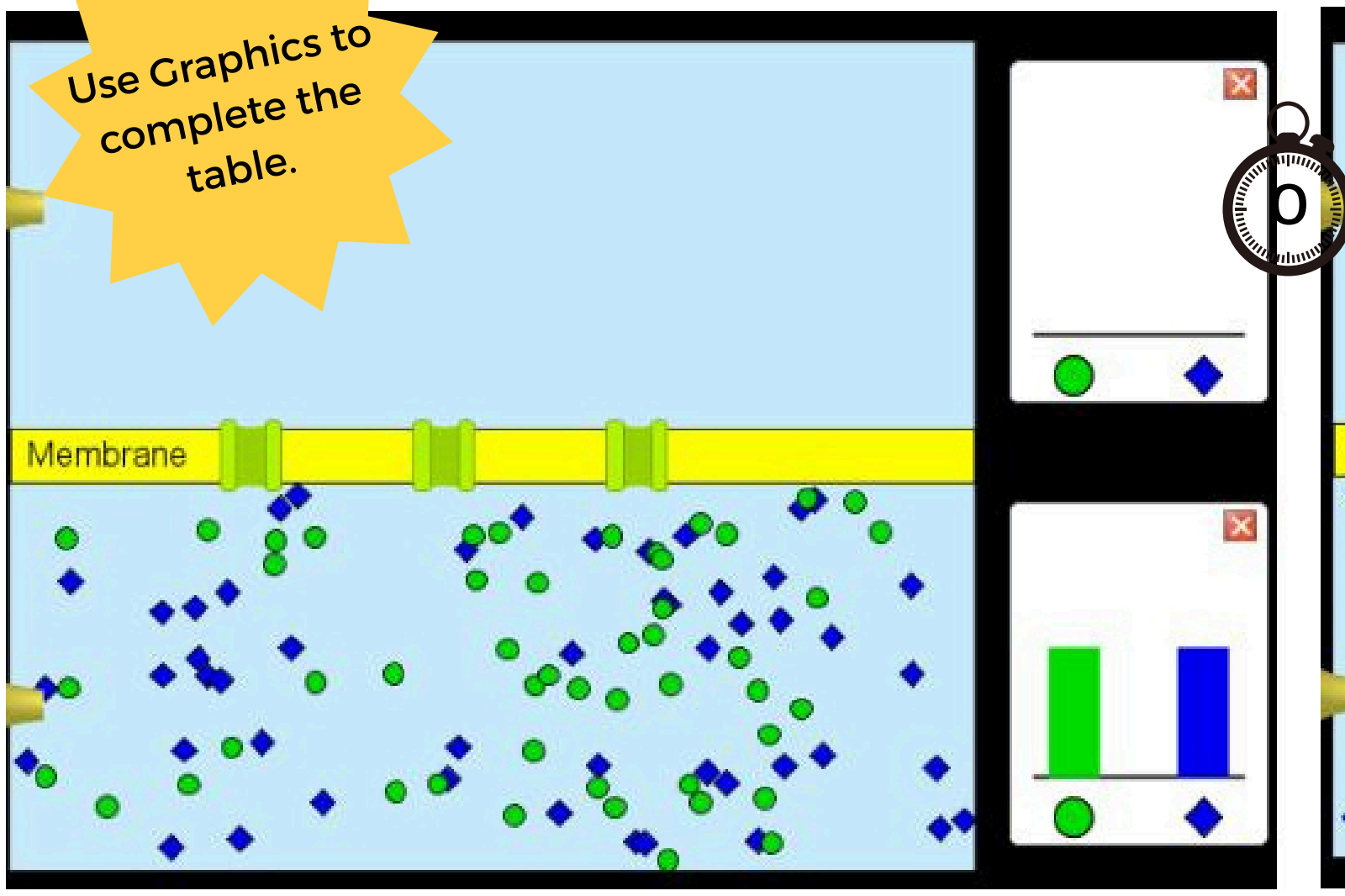

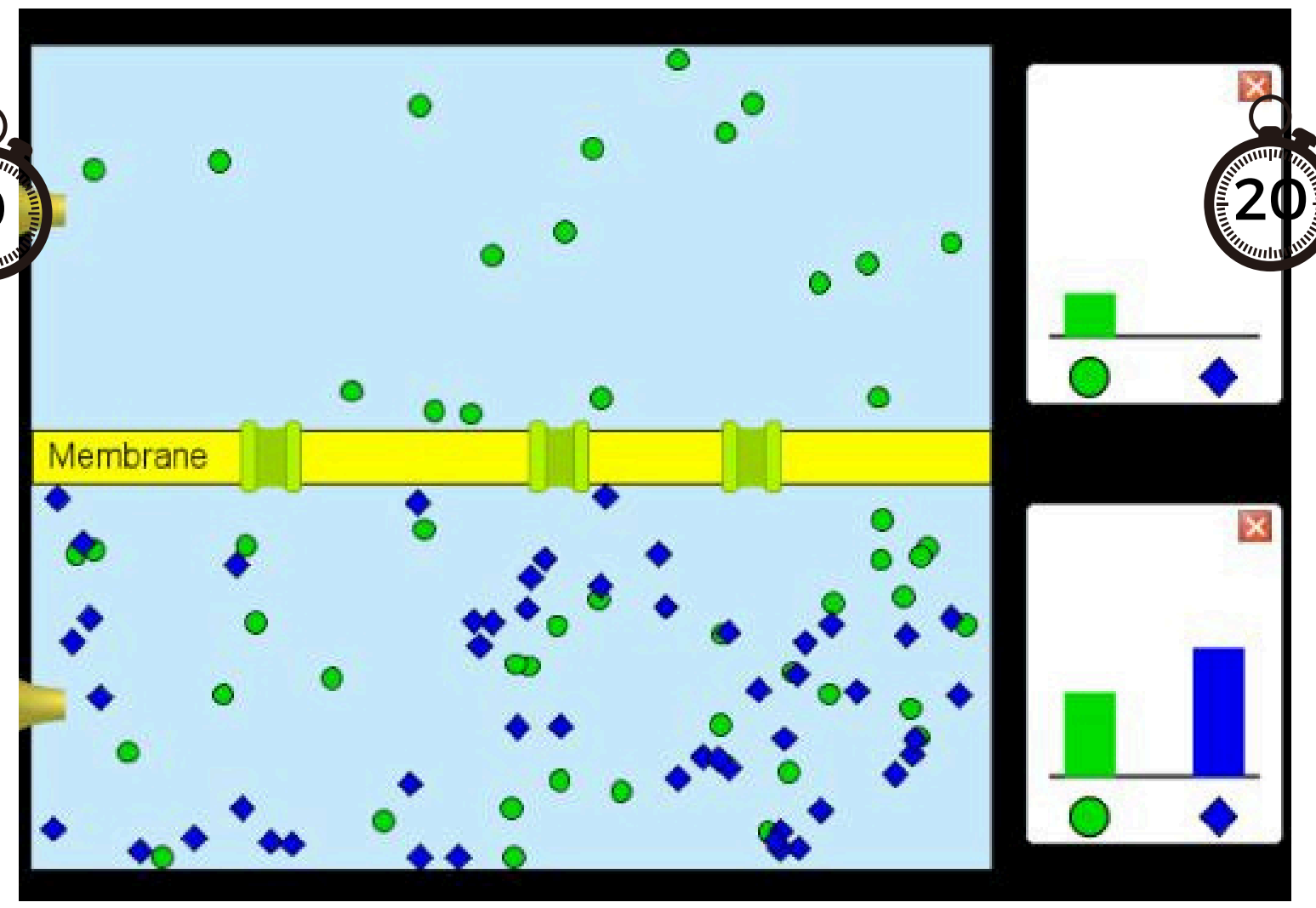

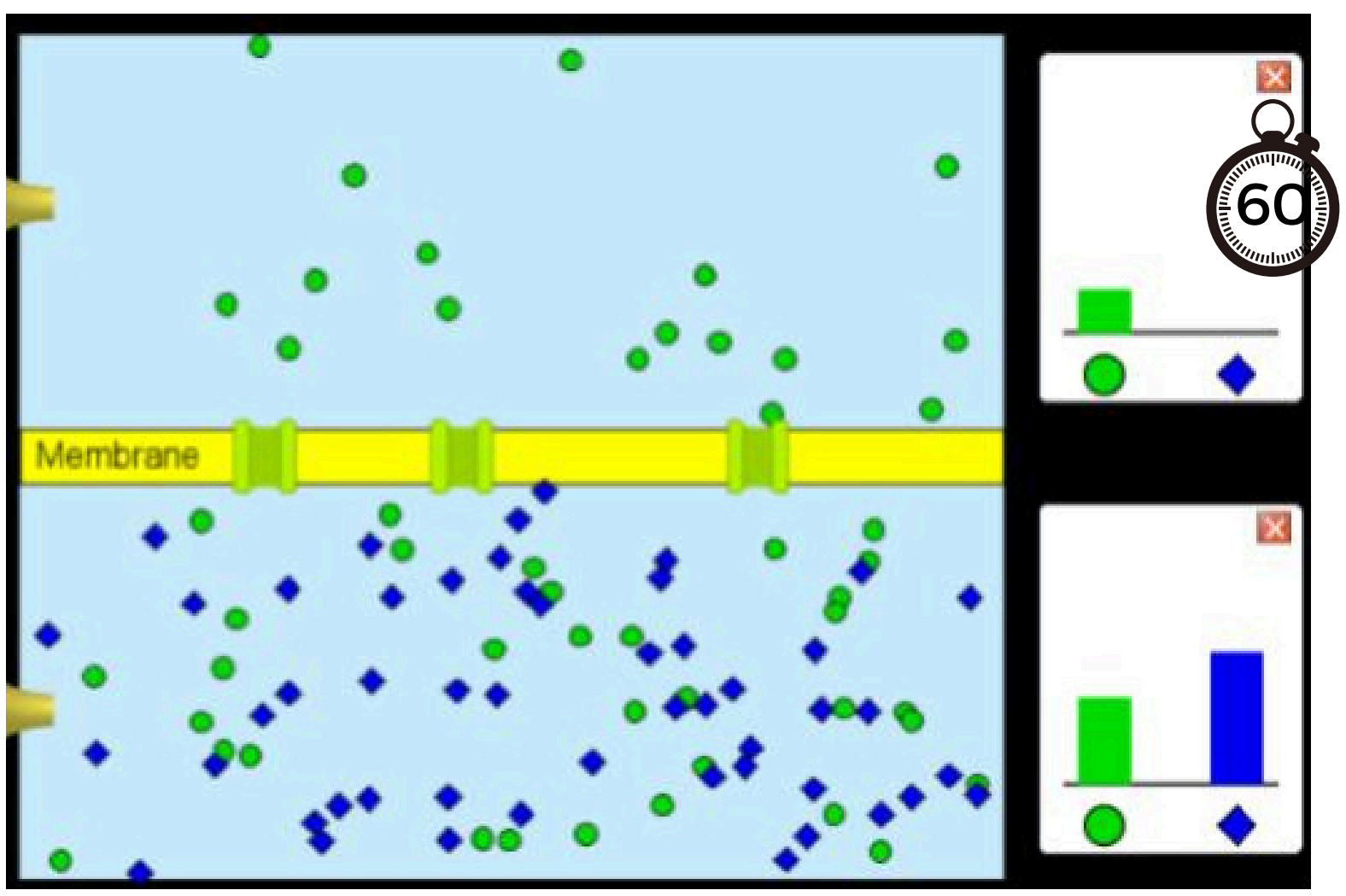

Question: Looking at the table, what do you notice when you compare the # of Na+ ions on the top and the bottom of the membrane at 20 and 60 secs? Why does this happen?

Question: In what direction or how did the Cl- ions move?

Question: Why were they only able to move across through a transport protein?

Question: At 20 secs are the Cl- ions at Equilibrium and how do you know?

Question: At 60 secs are the Cl- ions at Equilibrium?

Question: Why do you think Cl- ions do not reach Equilibrium? Think about the charge of Na+ ions that stayed on one side of the membrane.

Time	Start: 0 secs	20 secs	60 secs/ 1 min
# of Cl- Ions on Top			
# of Cl- Ions on Bottom			
# of Na+ Ions on Top			
# of Na+ Ions on Bottom			

Concept: Electrochemical Gradient is the combined effect of two forces that affect how ions (charged particles) move across a cell membrane. **Force 1: Chemical Gradient-** materials move from High to Low concentration. **Force 2: Electrical Gradient (charged forces)-** Ions move towards or pulled towards their opposite charge, **+ w/-**

Food Coloring Demo: Illustrate the Beaker at Start and End of Demo

What happens when I dropped the food coloring into the still water?

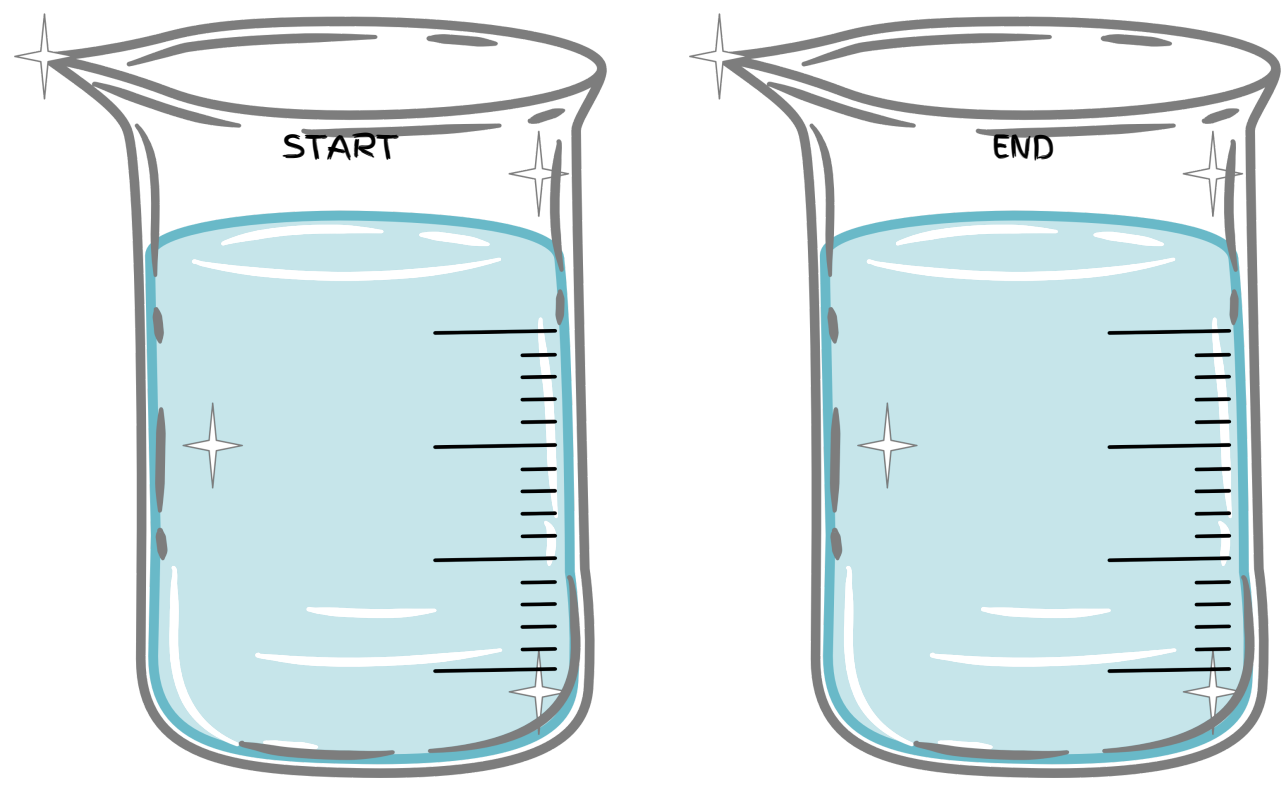

Did the food coloring spread out in the water without stirring?

Did any visual factors affect how fast the food coloring diffuses in water?

Was any heat, electricity or energy necessary for the food coloring to spread in water?

Concept:

Explore the Electrochemical Gradient

Directions: Write down as many obervations that you can from the graphics below

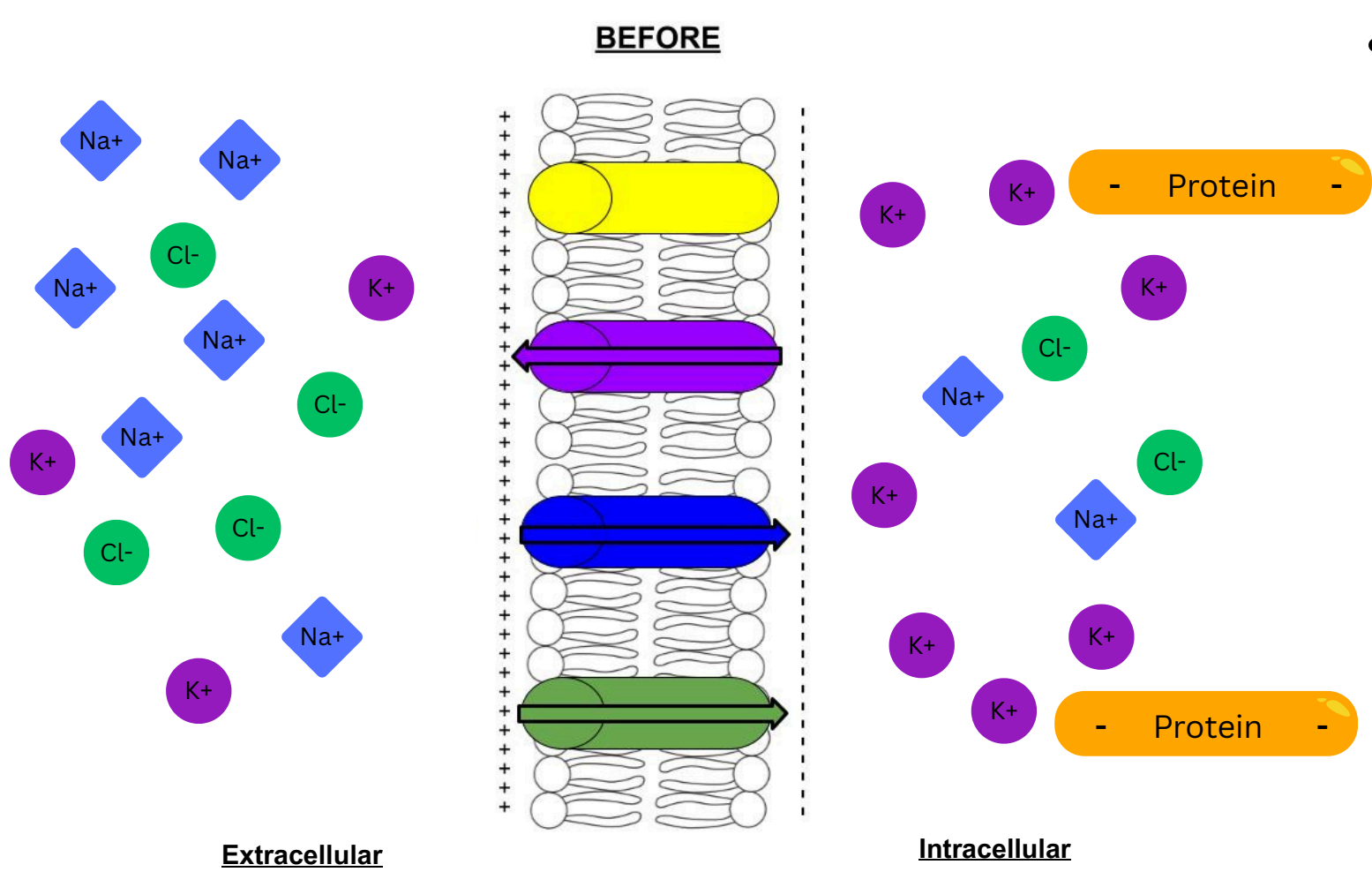

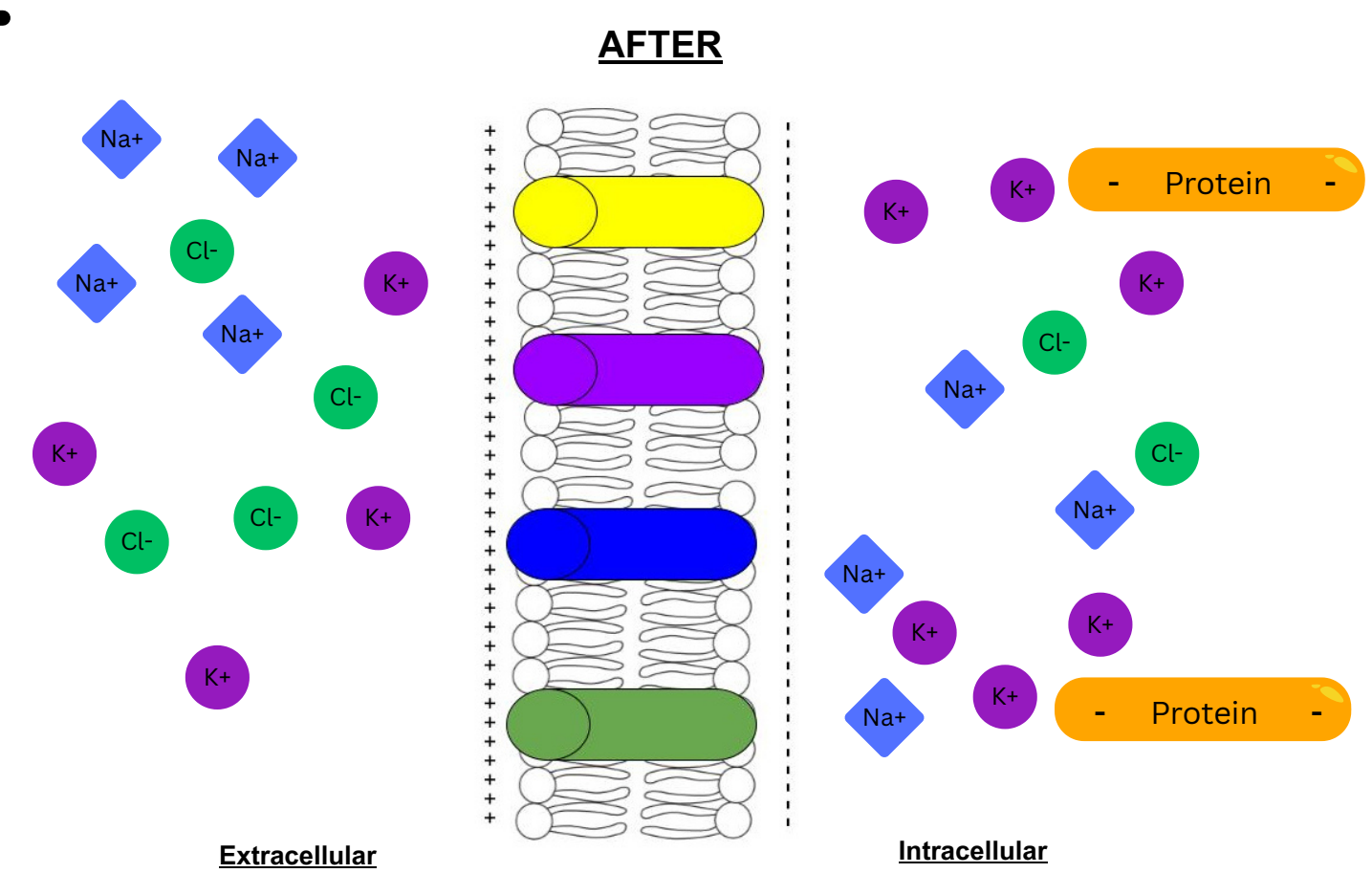

Cell Membrane and Cell Transport
Answer Sheet- Guided Notes

Food Coloring Demo: Illustrate the Beaker at Start and End of Demo

What happens when I dropped the food coloring into the still water?
The food coloring will spread from one spot out to the sides of the beaker.

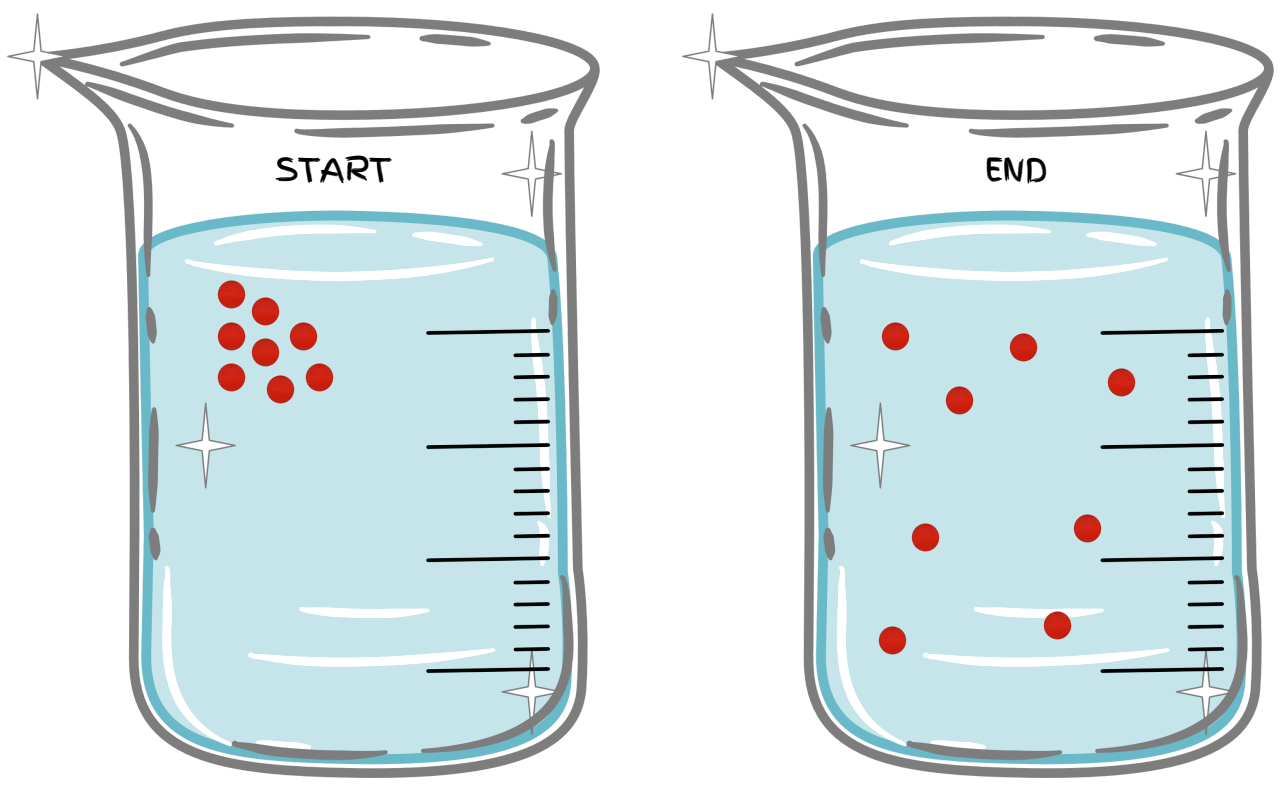

Did the food coloring spread out in the water without stirring? Yes

Did any visual factors affect how fast the food coloring diffuses in water?
No

Was any heat, electricity or energy necessary for the food coloring to spread in water? No

Concept: **Passive Transport:** Materials naturally move from High to low concentration **without the use of energy.** Materials move with the concentration gradient without needing energy.

Explore the Electrochemical Gradient

Directions: Write down as many obervations that you can from the graphics below

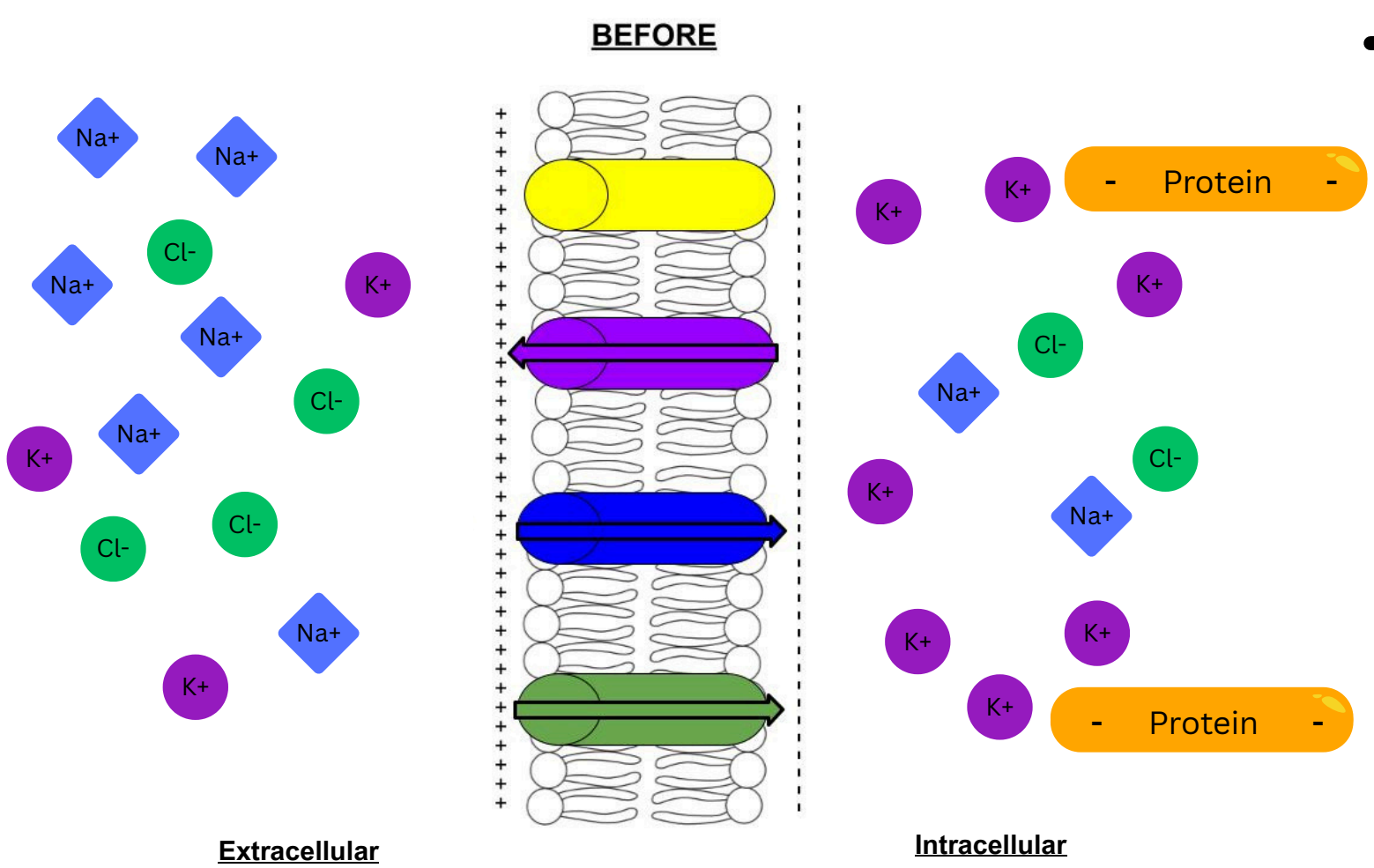

There are different transport proteins.
5 Na+ on the Extracelluar Side
and 2 Na+ on the Intracellular Side

4 Cl- on the Extracellular Side and 2 Cl- on the Intracellular Side

3 K+ on the Extracellular side and 7 K+ on the Intracellular Side

2 Proteins on the Intracellular Side

Extracellular Region is marked + and Intracellular -

K+ and Na+ give off a postive charge
Cl- and the Proteins give off a negative charge

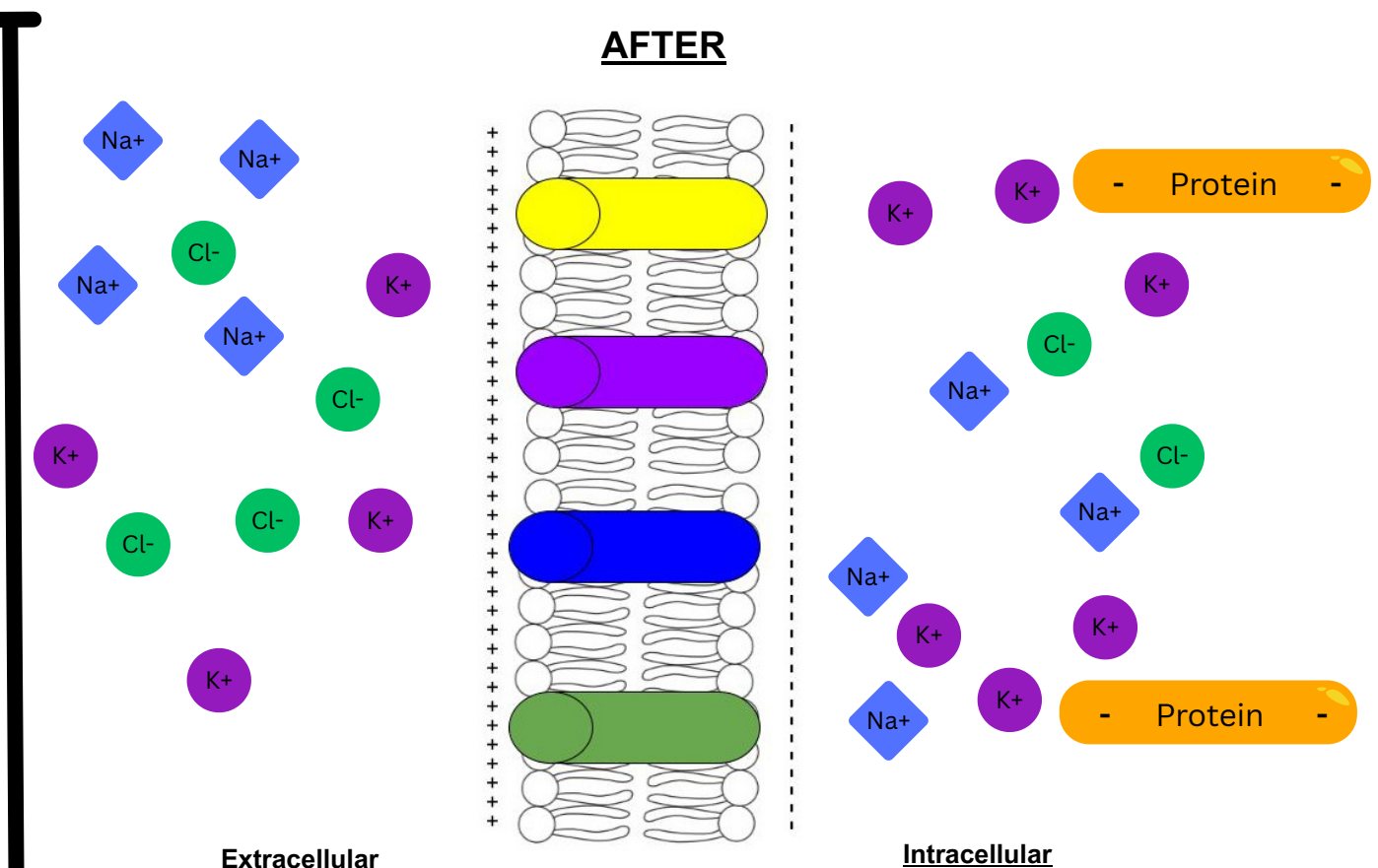

Na+ moved from High to Low and ends with 4 Na+ on either side, equal

K+ moved from High to Low, but they are not equal on both sides. There are more K+ on the negative Intracellular Side

Cl- did not change. There are more Cl- on the + Extracellular Side

The proteins remain where they started as well. They did not move.

Use the SImulation Direction Cards to complete the following guided notes

Cell Membrane Introduction:

What are the functions of the cell membrane?

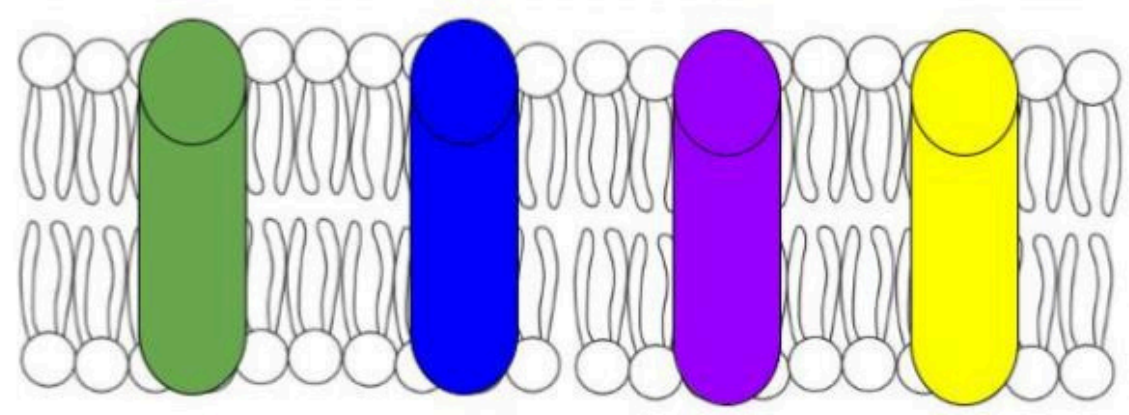

Label all the parts of the cell membrane diagram

What is the Phospholipid Bilayer Composed of and why is it called a Bilayer?

What is the difference between Hydrophilic and Hydrophobic?

What is the function of Transport proteins?

Is the Cell Membrane completely solid?

Could a molecule flow freely through the **Phospholipid Bilayer?**

But what would be the requirment of the molecule?

Concept

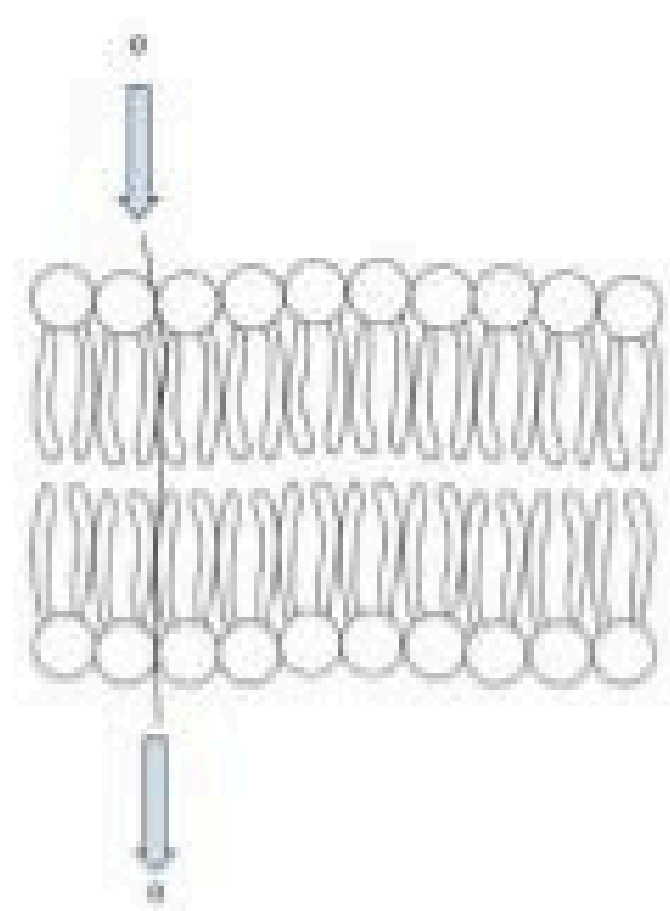

Use the SImulation Direction Cards to complete the following guided notes

Cell Membrane Introduction:

What are the functions of the cell membrane?

-Surrounds the cell

-Creates a barrier between the inside and outside of the cell

-Controls what goes in and out of the cell

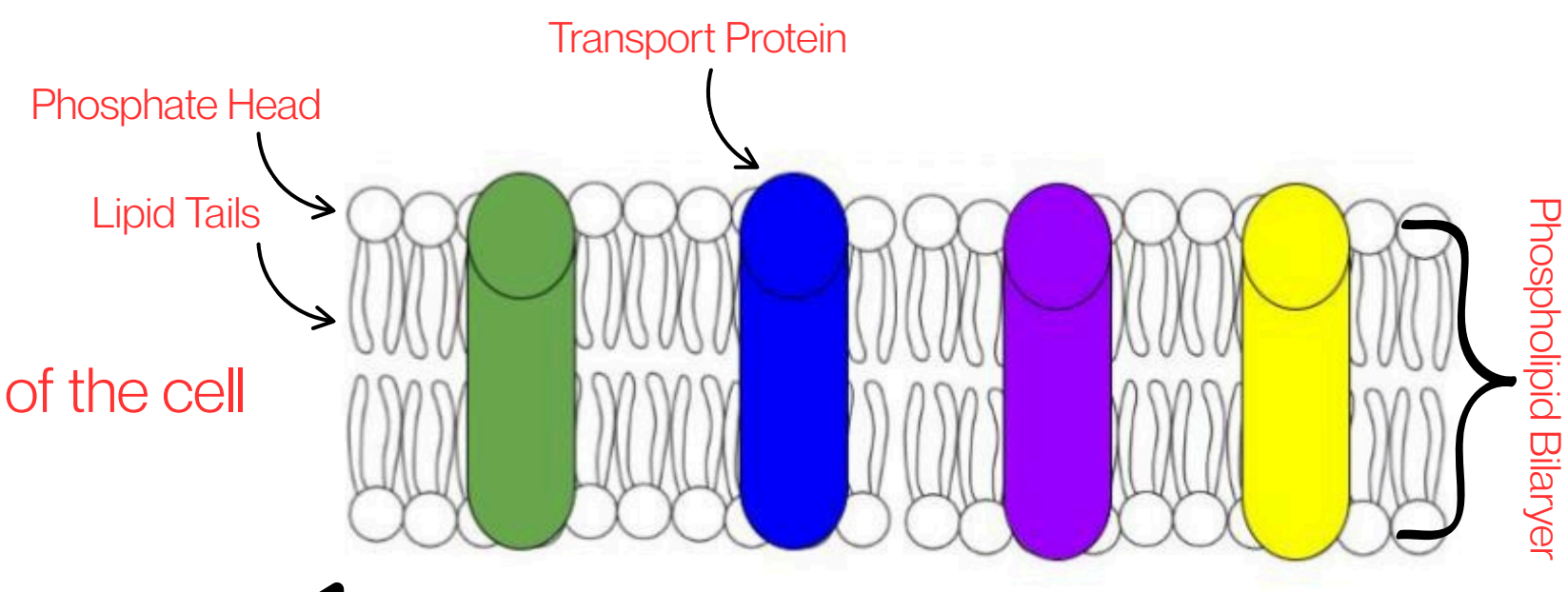

Label all the parts of the cell membrane diagram

What is the Phospholipid Bilayer Composed of and why is it called a Bilayer?

It is composed of TWO layers of Phosphate Heads connect to Lipid Tails.

What is the difference between Hydrophilic and Hydrophobic?

Hydrophilic- attracts water and Hydrophoic- repels water

What is the function of Transport proteins?

The help materials cross in and out of the cell.

Is the Cell Membrane completely solid?

No there are tiny gaps/spaces between the phosphate heads.

Could a molecule flow freely through the **Phospholipid Bilayer?**

Yes

But what would be the requirment of the molecule?

They would have to be small.

Concept: **Simple Diffusion-** Small materials move from High to Low concentration through the Phospholipid Bilayer, directly through the cell membrane

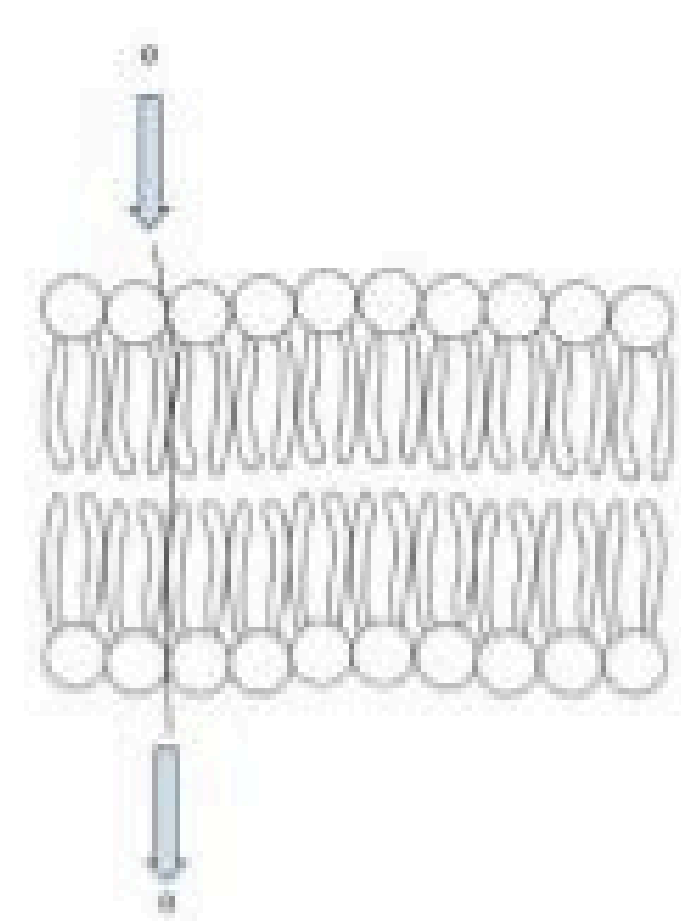

Do materials just move in and out of the cell with no direction?

Question: In what direction or how did the Cl- ions move?

Looking at the table, what do you notice when you compare the # of Cl-ions on one side to the other side of the Membrane?

**Time**	**Start: 0 secs**	**20 secs**	**60 secs/ 1 min**
# of Cl- Ions on Top			
# of Cl- Ions on Bottom			

Concept:

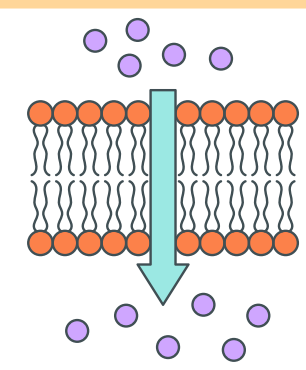

What if the material is TOO big to fit directly through the Phospholipid Bilayer?

Question: What do you observe about the Cl- Ions?

Question: What do you observe about the Cl- ions when the gate proteins are added?

Question: In what direction did the Cl- Ions move? **Think about what you learned from the previous run.**

Concept:

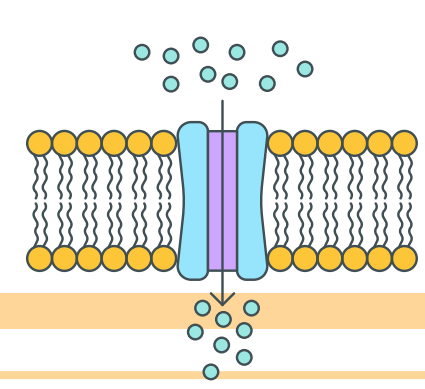

Do materials just move in and out of the cell with no direction?

Question: In what direction or how did the Cl- ions move?

From a high amount to a low amount. From a large amount to where there were none. From High To low concentration.

Looking at the table, what do you notice when you compare the # of Cl-ions on one side to the other side of the Membrane?

At 0 secs, all the Cl- ions were on one side. At 20 secs, the Cl- ions moved across and they were close to the same amount on either side. At 60 secs, the Cl- ions were still close to the same amount on either side of the membrane.

These answers can vary depending on which side they added the Cl- ions. As well as the numbers at 20 & 60 Secs; however, the numbers should be close to equal.

Time	Start: 0 secs	20 secs	60 secs/ 1 min
# of Cl- Ions on Top	40	18	20
# of Cl- Ions on Bottom	0	22	20

Concept: Diffusion- Materials naturally move from Hight to Low Concentration until reaching **Equilibrium**.

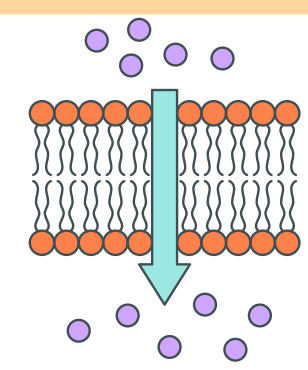

What if the material is TOO big to fit directly through the Phospholipid Bilayer?

Question: What do you observe about the Cl- Ions?

The Cl- ions did not go directly through the membrane.

Question: What do you observe about the Cl- ions when the gate proteins are added?

The Cl- ions were able to pass through to the other side through the gate/transport protein.

Question: In what direction did the Cl- Ions move? **Think about what you learned from the previous run.**

The Cl- ions moved from High to low concentration.

Concept: Facilitated Diffusion- Large materials (that are too big to pass through the membrane) use a transport protein to move from High to Low concentration.

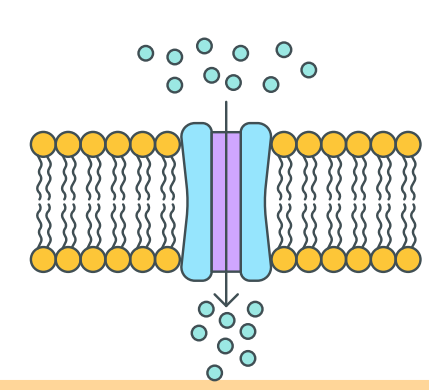

Can any molecules go through a gate protein?

Question: After opening the blue gate proteins, What do you observe?

Question: After opening the green gate proteins, What do you observe?

Question: After adding the blue Na+ ions, what do you observe?

Question: So what can you conclude about gate proteins?

Concept:

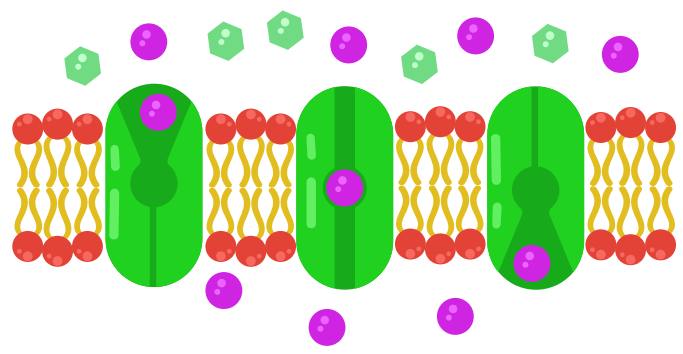

Why do materials move? Is there a driving force?

Question: When you started the sim there are 40 Cl- ions on both sides of the membrane, that means you started the sim at

Question: During the sim was there a big shift of Cl- ions to either side of the membrane? Y or N

Question: After adding 20 more Cl- ions, now the concentrations on both sides of the membrane are

Question: After adding 20 more Cl- ions and you allow the sim to run, What do you notice about the concentrations on both sides of the membrane?

Question: How did this happen? Where did the Cl- ions go or how did they move?

Concept:

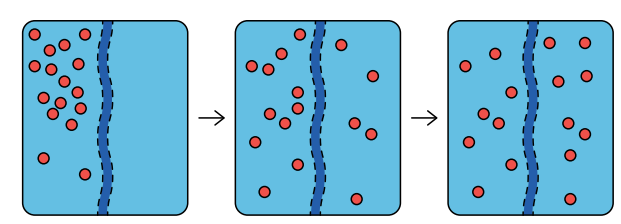

Can any molecules go through a gate protein?

Question: After opening the blue gate proteins, What do you observe?
The Cl- ions did not go through the blue gate protein.

Question: After opening the green gate proteins, What do you observe?
The Cl- ions were able to go through the green gate proteins.

Question: After adding the blue Na+ ions, what do you observe?
The Na+ ions were able to go through the blue gate proteins.

Question: So what can you conclude about gate proteins?
Only certain materials can go through specific transport/gate proteins.

Concept: There are specific gate/transport proteins for specific materials to move in and out of the cell.

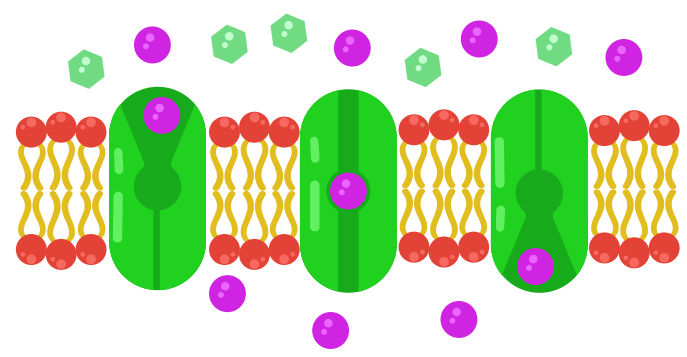

Why do materials move? Is there a driving force?

Question: When you started the sim there are 40 Cl- ions on both sides of the membrane, that means you started the sim at Equilibruim.

Question: During the sim was there a a big shift of Cl- ions to either side of the membrane? Y or N (circled: N)

Question: After adding 20 more Cl- ions, now the concentrations on both sides of the membrane are Different

Question: After adding 20 more Cl- ions and you allow the sim to run, What do you notice about the concentrations on both sides of the membrane?
There was a big shift of Cl- ions to the opposite side (the side the 20 Cl- ions were NOT added).

Question: How did this happen? Where did the Cl- ions go or how did they move?
There was a higher concentration on one side of the membrane, so the Cl- ions were able to move from High to Low concentration.

Concept: In order for diffusion to occure, there must be a difference in concentration inside and outside the cell, so the materials can move from High to low concentrations which is called the **Concentration Gradient.**

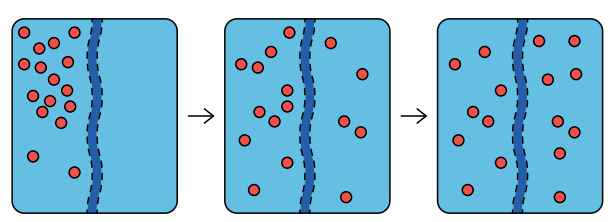

Is the only driving force for Diffusion the Concentration/Chemical Gradient?

Question Na+ ions give off what charge?

Question: Cl- ions give off what charge?

Question: When you put 2 postives together they?

Question: When you put 2 negatives together they?

Question When you put a negative and positive together they?

Time	Start: 0 secs	20 secs	60 secs/ 1 min
# of Cl- Ions on Top			
# of Cl- Ions on Bottom			
# of Na+ Ions on Top			
# of Na+ Ions on Bottom			

Use your data table to answer the following questions:
Question: What do you notice when you compare the # of Na+ ions on the top and the bottom of the membrane at 0, 20, and 60 sec(s)?

Why?

Question: In what direction did the Cl- ions move?

Question: At 20 secs are the Cl- ions at Equilibrium? How do you know?

Question: At 60 secs are the Cl- ions at Equilibrium? Y or N

Question: Why do you think the Cl- ions do not reach Equilibrium? **Think about the charge of the Na+ and that stayed on ONE side of the membrane.**

Concept:

Is the only driving force for Diffusion the Concentration/Chemical Gradient?

Question Na+ ions give off what charge? Positive

Question: Cl- ions give off what charge? Negative

Question: When you put 2 positives together they? They repel or move away from each other.

Question: When you put 2 negatives together they? They repel or move away from each other.

Question When you put a negative and positive together they? They are attracted to each other.

Use your data table to answer the following questions:
Question: What do you notice when you compare the # of Na+ ions on the top and the bottom of the membrane at 0, 20, and 60 sec(s)?
They stayed on one side of the membrane
Why?
Only Cl- gate proteins were present, and gate proteins are specific.

Question: In what direction did the Cl- ions move?
From High to Low Concentration

Time	Start: 0 secs	20 secs	60 secs/ 1 min
# of Cl- Ions on Top	0	17	17
# of Cl- Ions on Bottom	50	33	33
# of Na+ Ions on Top	0	0	0
# of Na+ Ions on Bottom	50	50	50

Question: At 20 secs are the Cl- ions at Equilibrium? How do you know? No, the concentrations are different on either side of the membrane.

Question: At 60 secs are the Cl- ions at Equilibrium? Y or N

Question: Why do you think the Cl- ions do not reach Equilibrium? **Think about the charge of the Na+ and that stayed on ONE side of the membrane.**
The Cl- ions want to move from High to low concentration, but the Na+ positive ions are pulling the negative Cl- ions towards them.

Concept: Electrochemical Gradient is the combined effect of two forces that affect how ions (charged particles) move across a cell membrane.
Force 1: Chemical Gradient (Concentration Gradient) Materials moving from High to Low Concentration.
Force 2 Electrical Gradient (Charged Forces) ions move towards or pulled towards their opposite charge, + w/-

1 What would happen if this system was put into a living organism?

How would the Ions Move?? Be prepared to write-out, illustrate, and verbally express your conclusion.

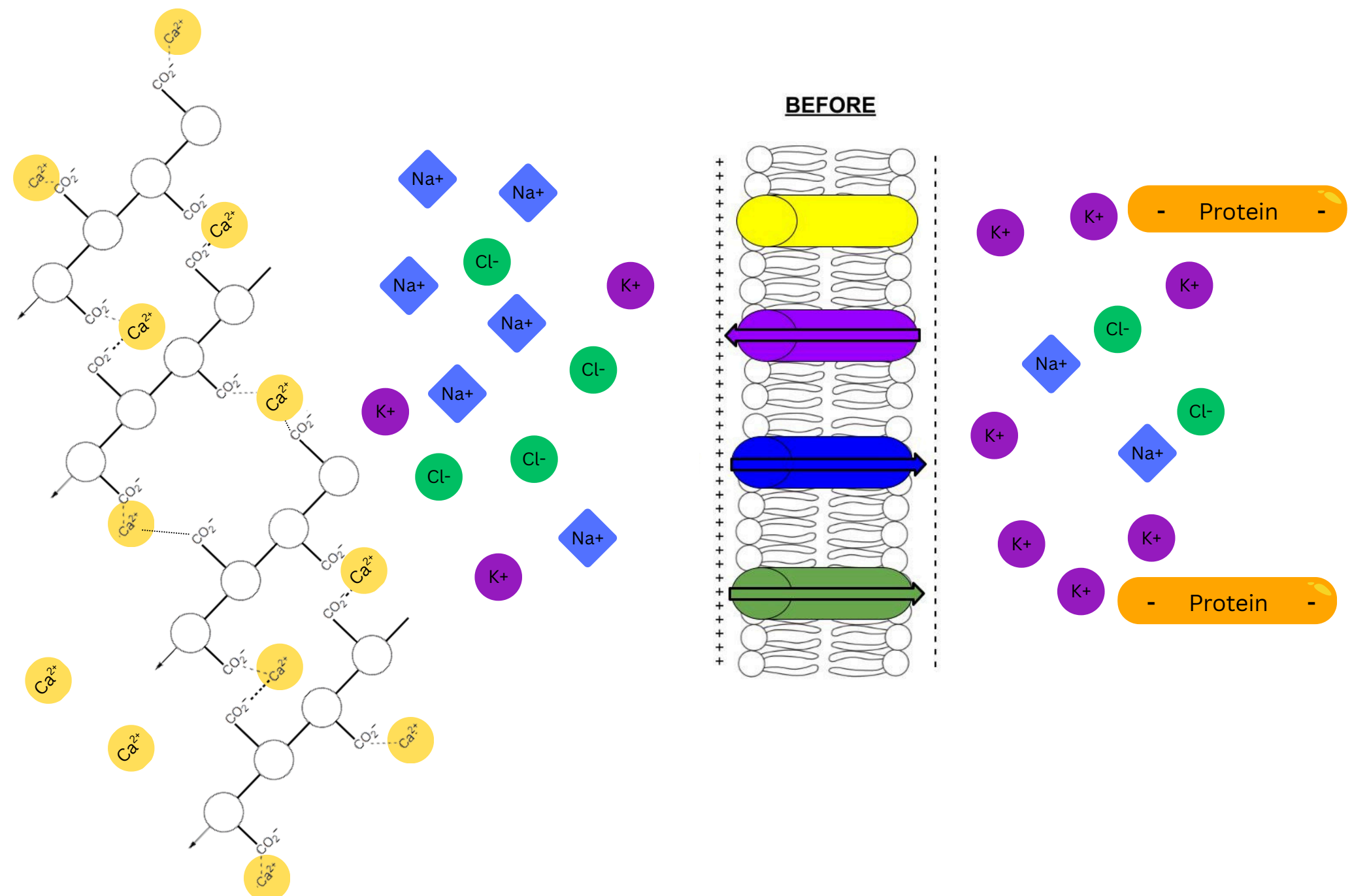

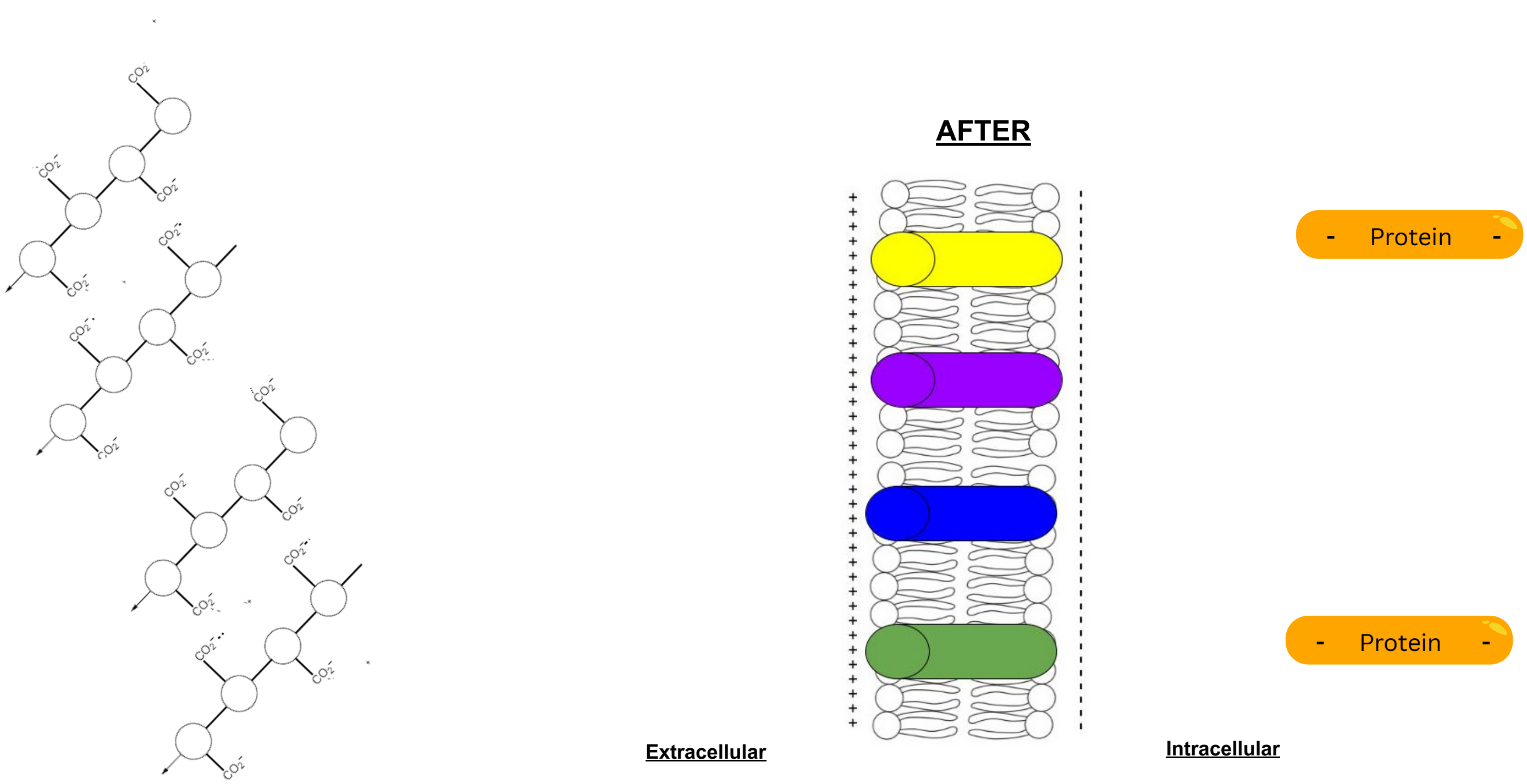

What would happen to the drug delivery hydrogel?

What would happen if this system was put into a living organism?

How would the Ions Move?? Be prepared to write-out, illustrate, and verbally express your conclusion.

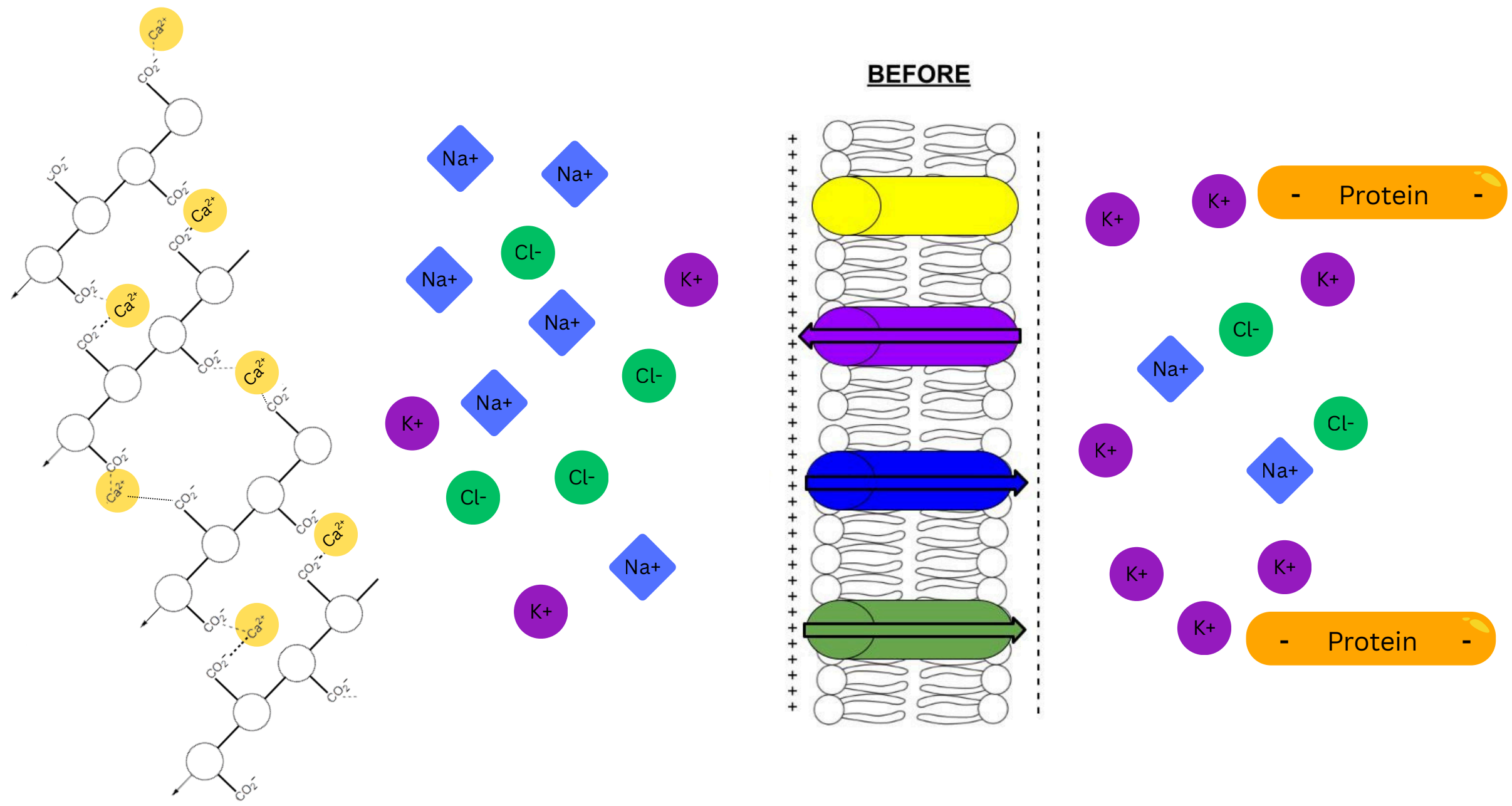

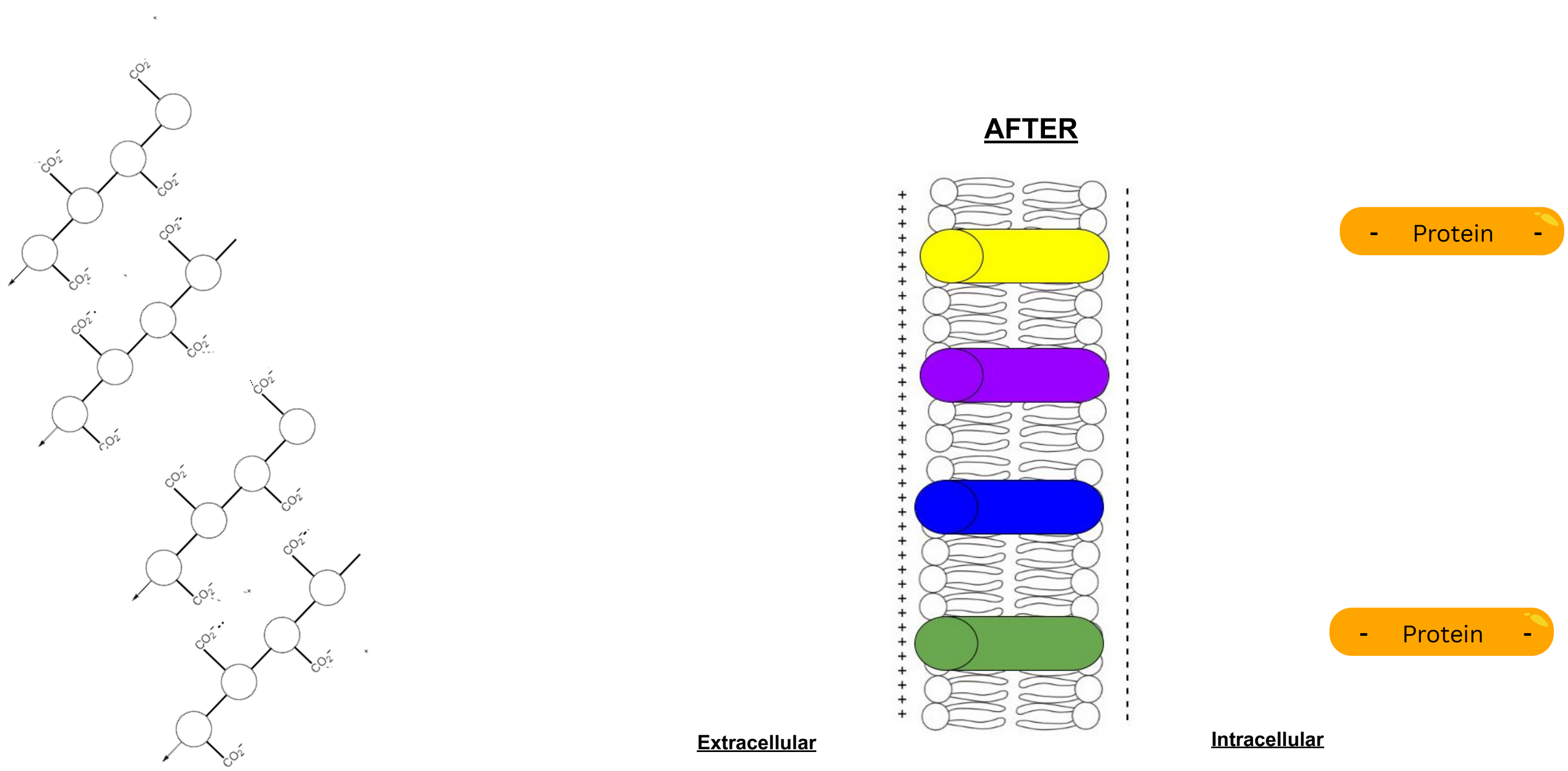

What would happen to the drug delivery hydrogel?

Make the Connection

What would happen if this system was put into a living organism?

How would the Ions Move?? Be prepared to write-out, illustrate, and verbally express your conclusion.

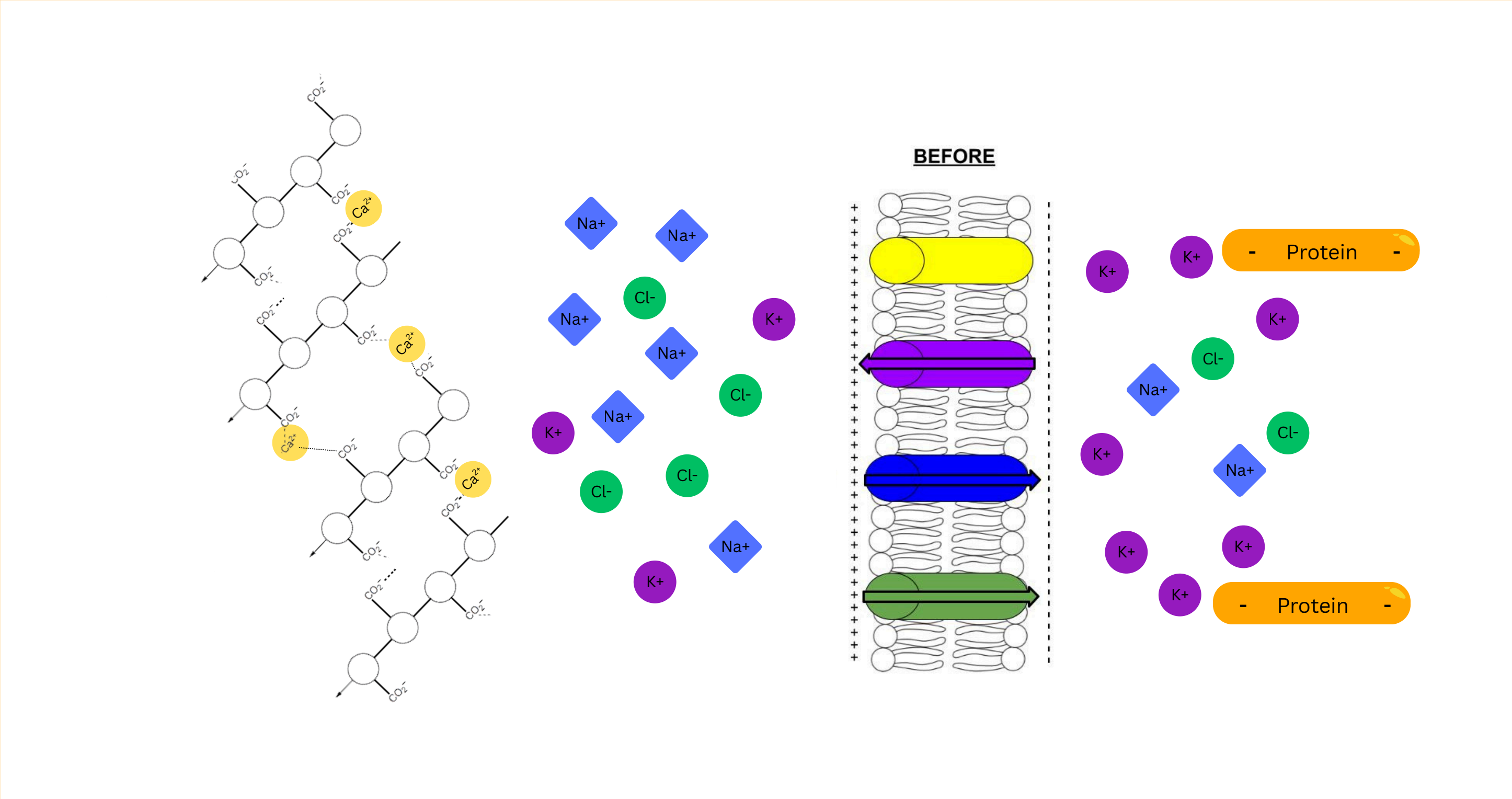

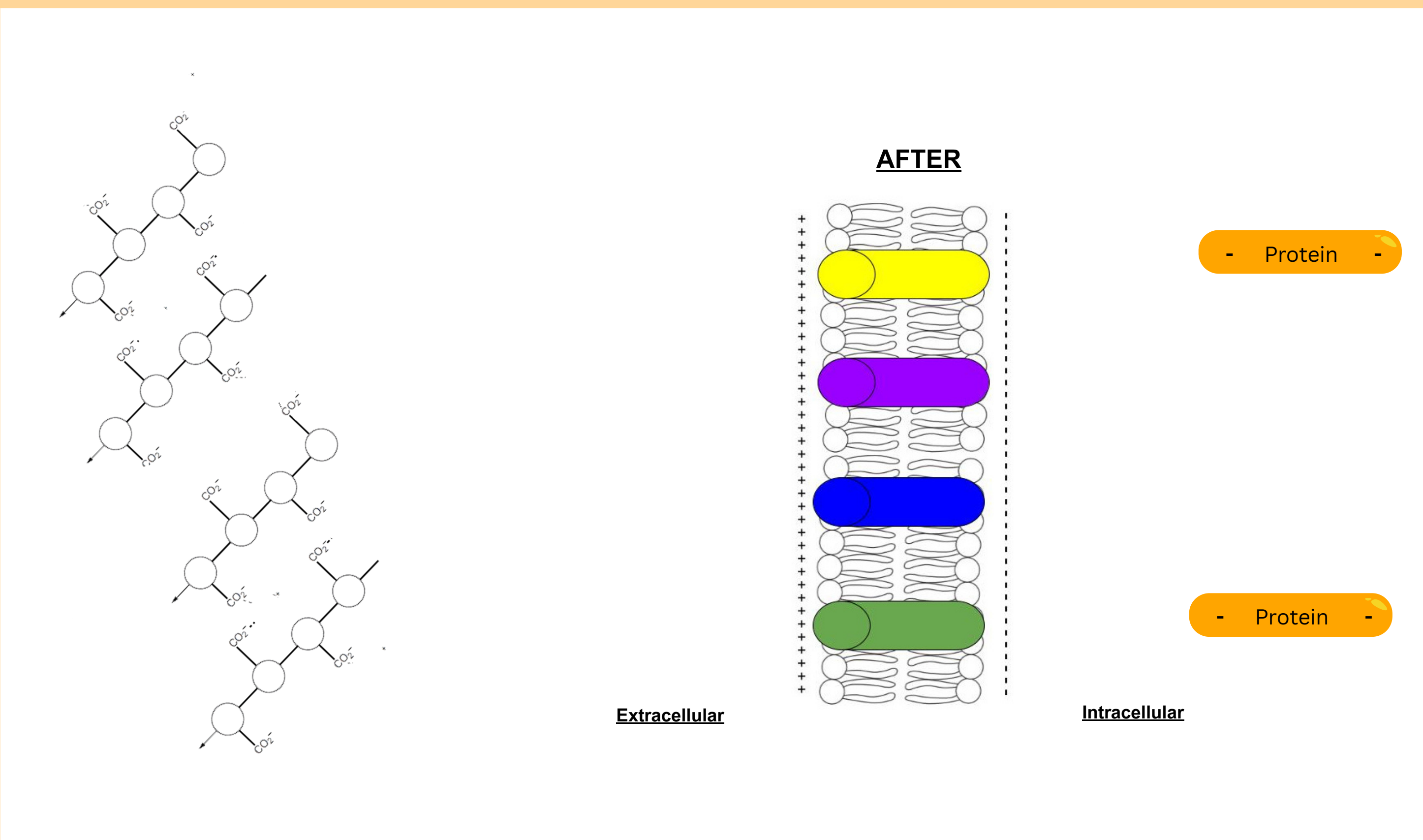

What would happen to the drug delivery hydrogel?

Make the Connection

What would happen if this system was put into a living organism?

How would the Ions Move?? Be prepared to write-out, illustrate, and verbally express your conclusion.

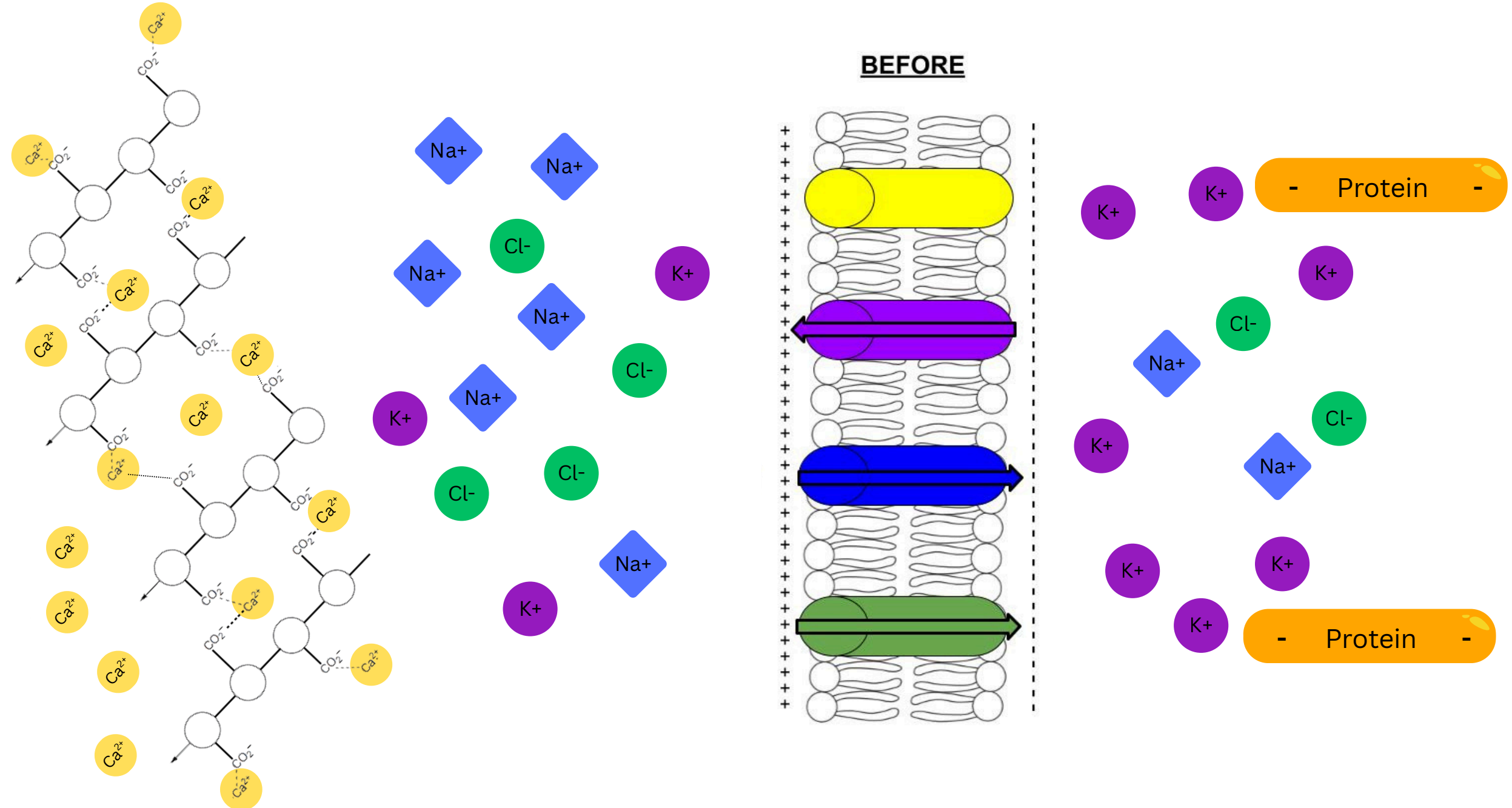

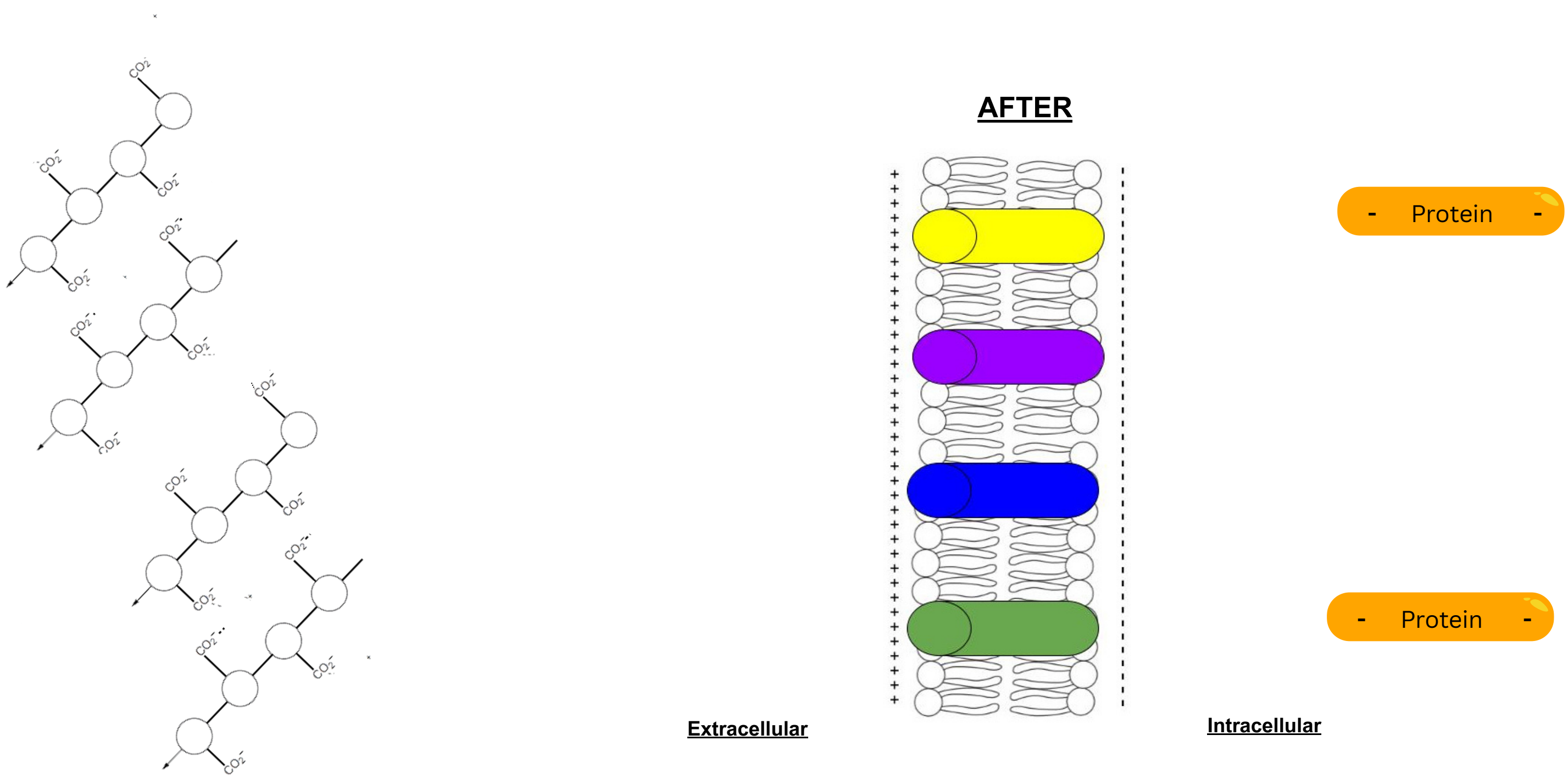

What would happen to the drug delivery hydrogel?

5

Make the Connection
What would happen if this system was put into a living organism?

How would the Ions Move?? Be prepared to write-out, illustrate, and verbally express your conclusion.

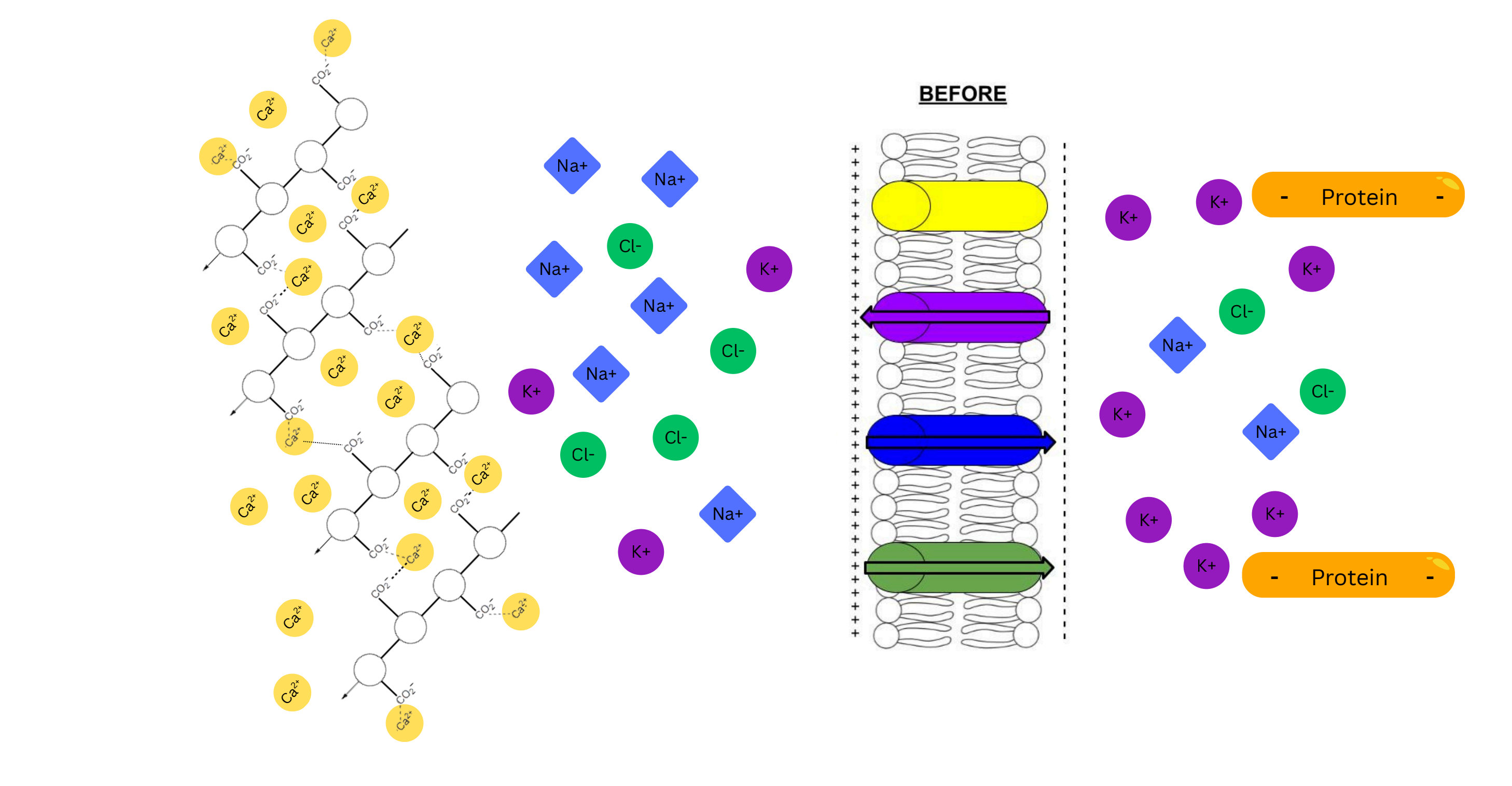

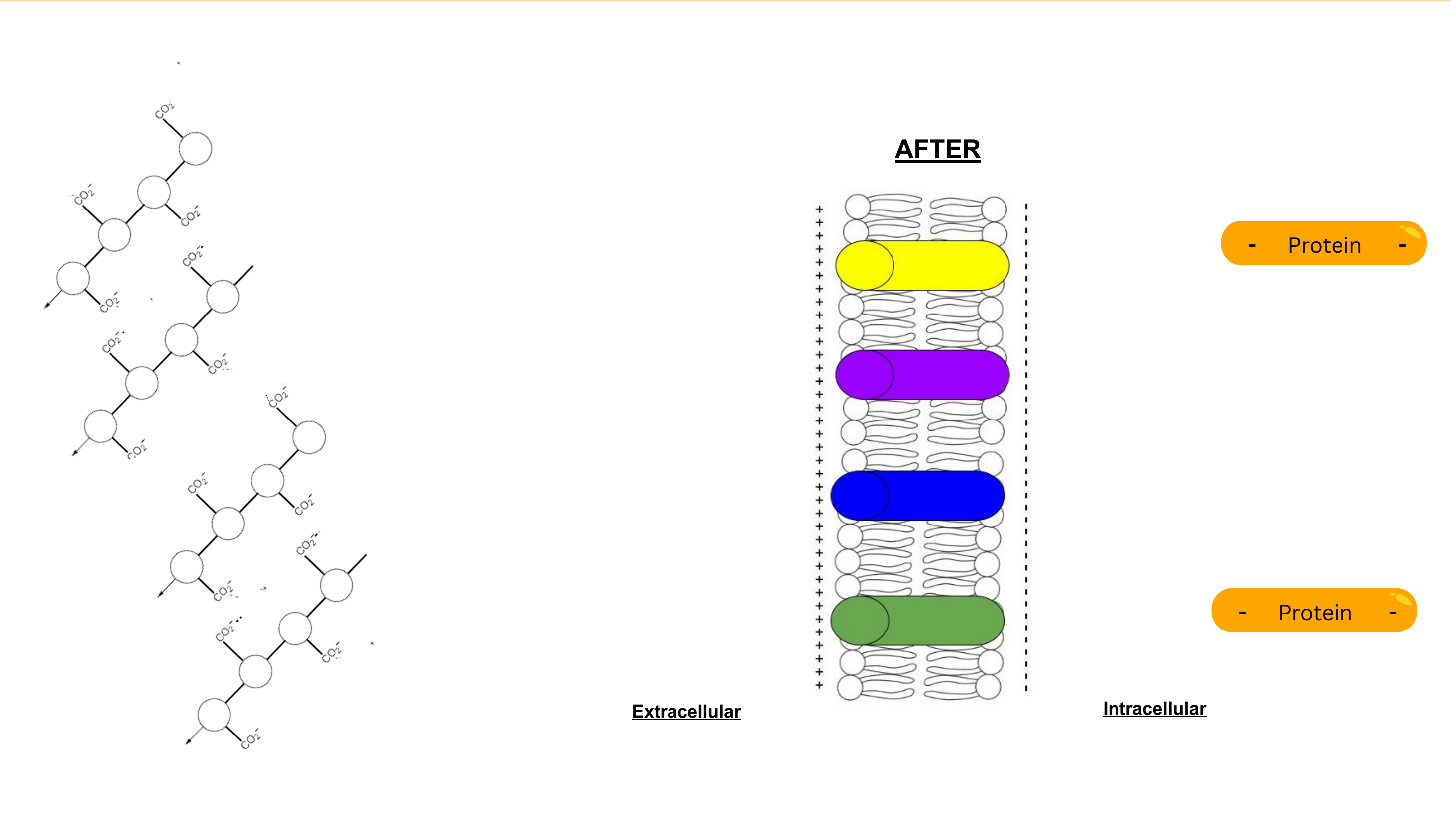

What would happen to the drug delivery hydrogel?

Make the Connection

What would happen if this system was put into a living organism?

How would the Ions Move?? Be prepared to write-out, illustrate, and verbally express your conclusion.

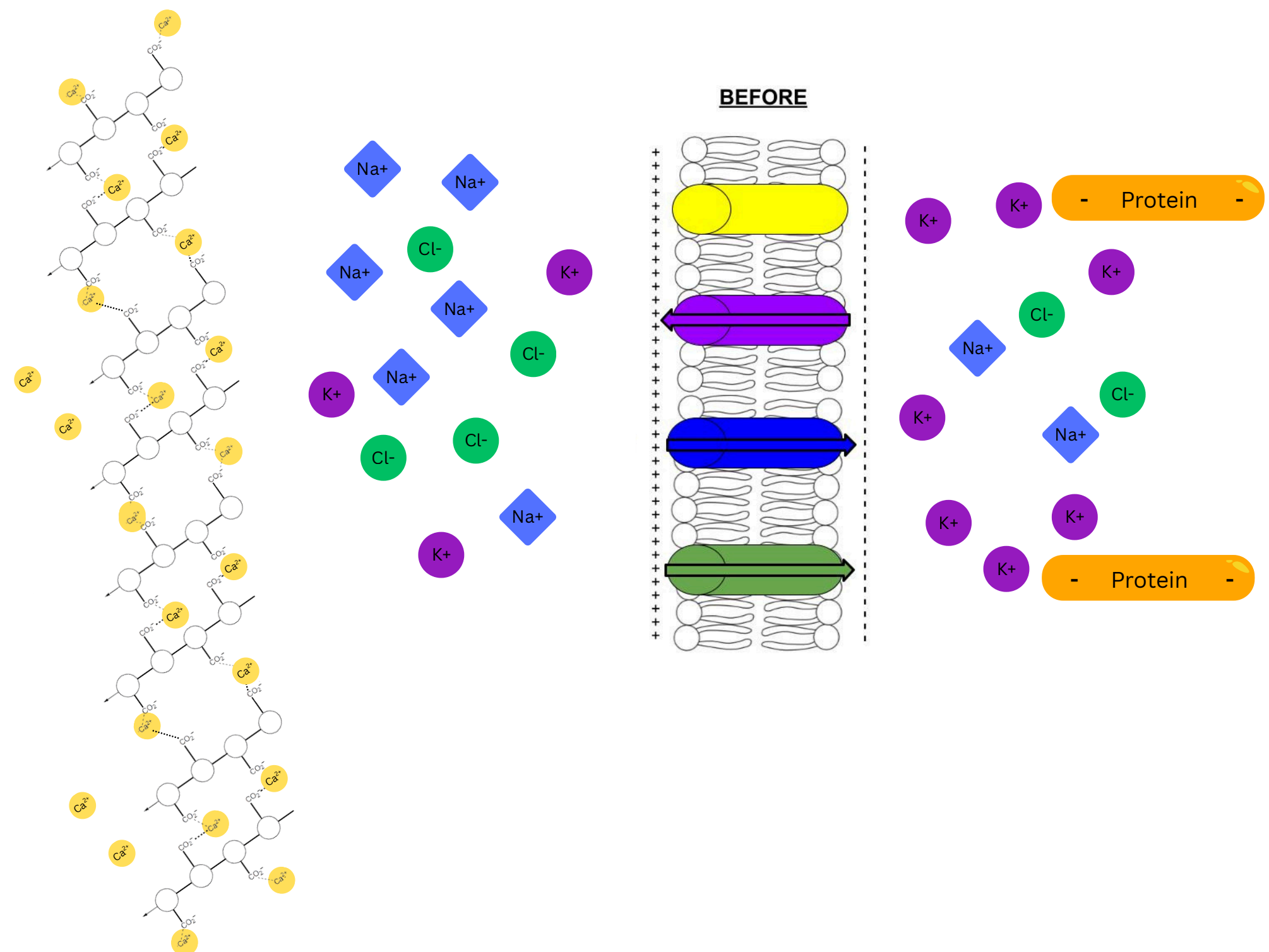

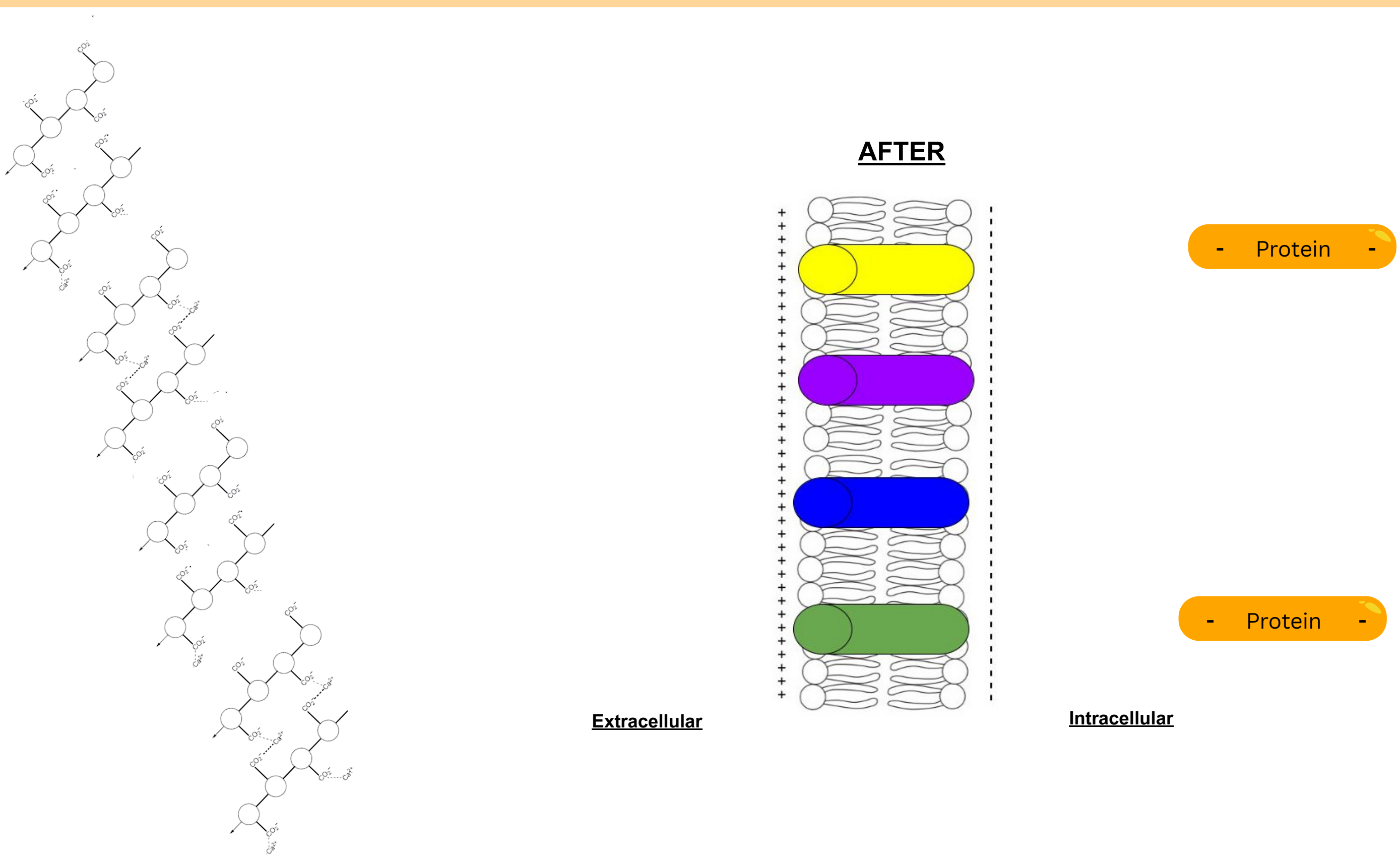

What would happen to the drug delivery hydrogel?

Make the Connection

7 What would happen if this system was put into a living organism?

How would the Ions Move?? Be prepared to write-out, illustrate, and verbally express your conclusion.

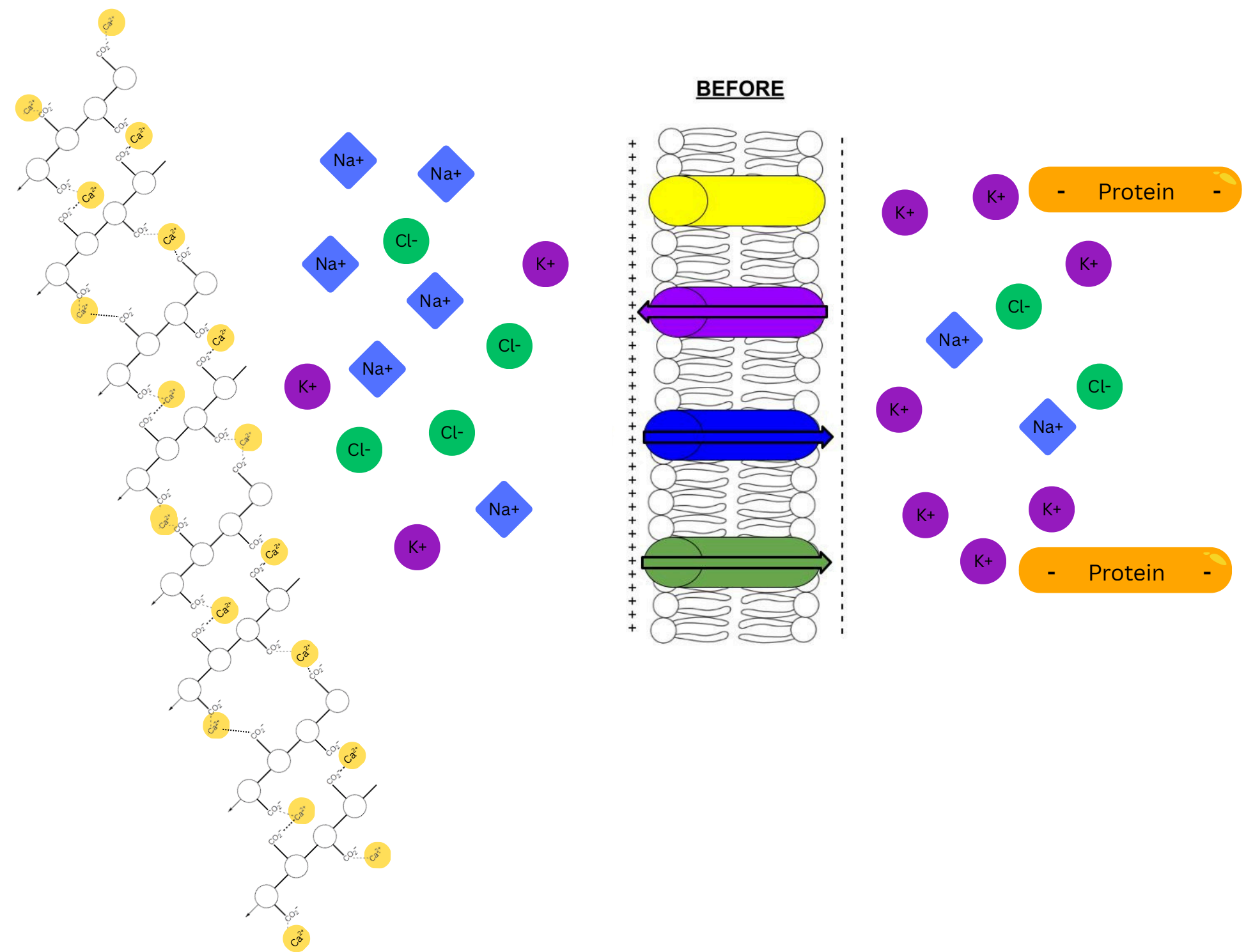

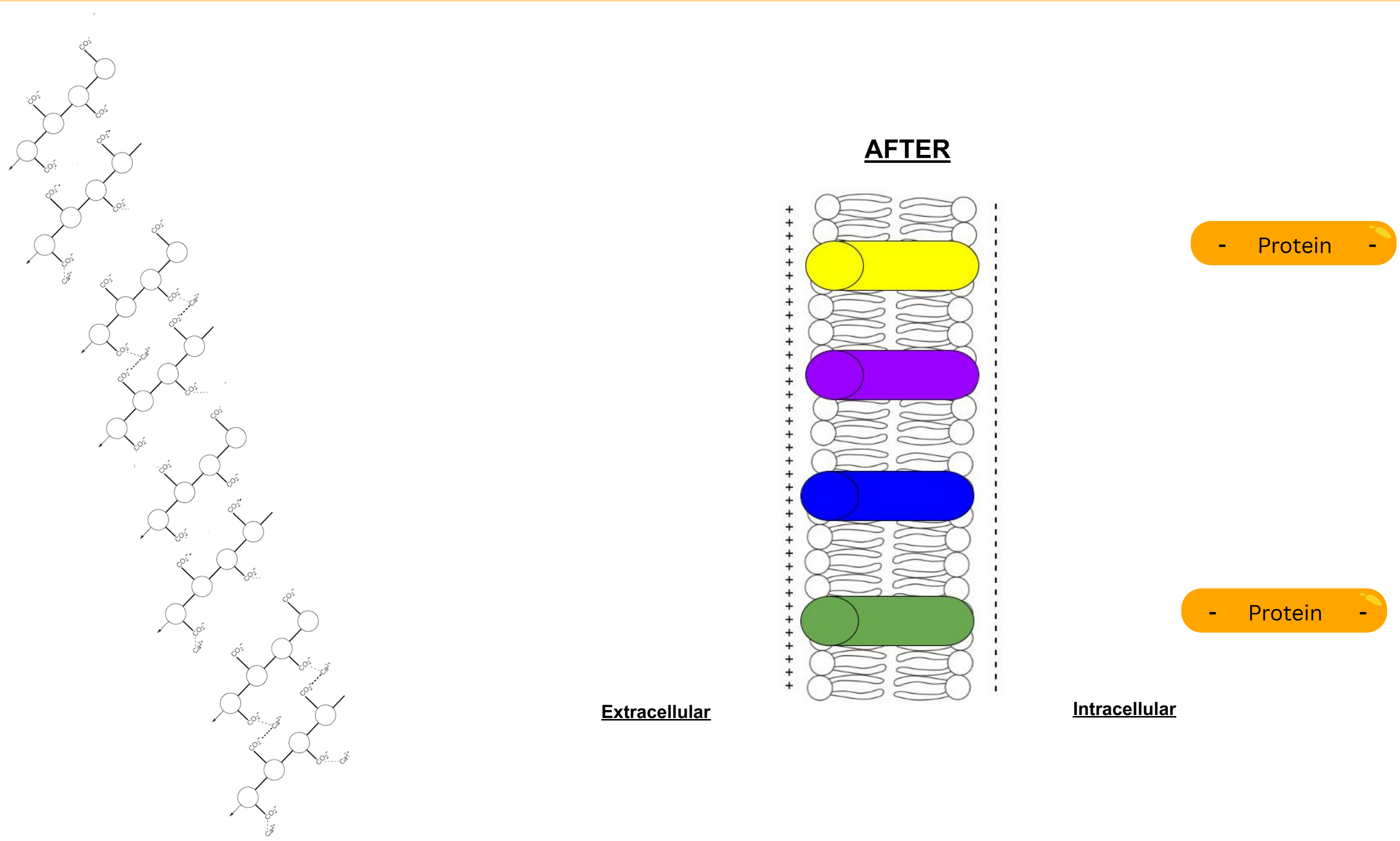

What would happen to the drug delivery hydrogel?

Make the Connection

What would happen if this system was put into a living organism?

<u>How would the Ions Move??</u> Be prepared to write-out, illustrate, and verbally express your conclusion.

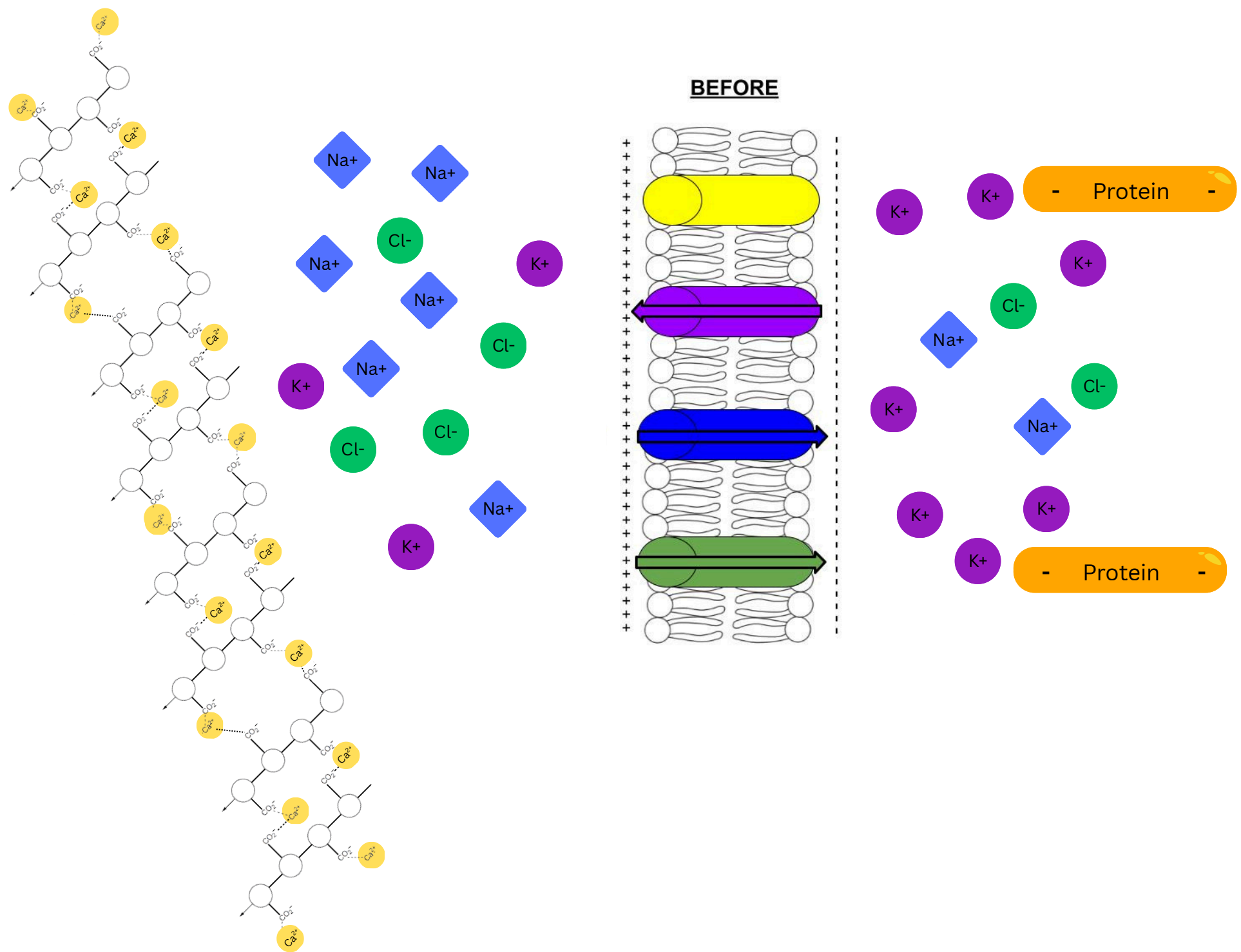

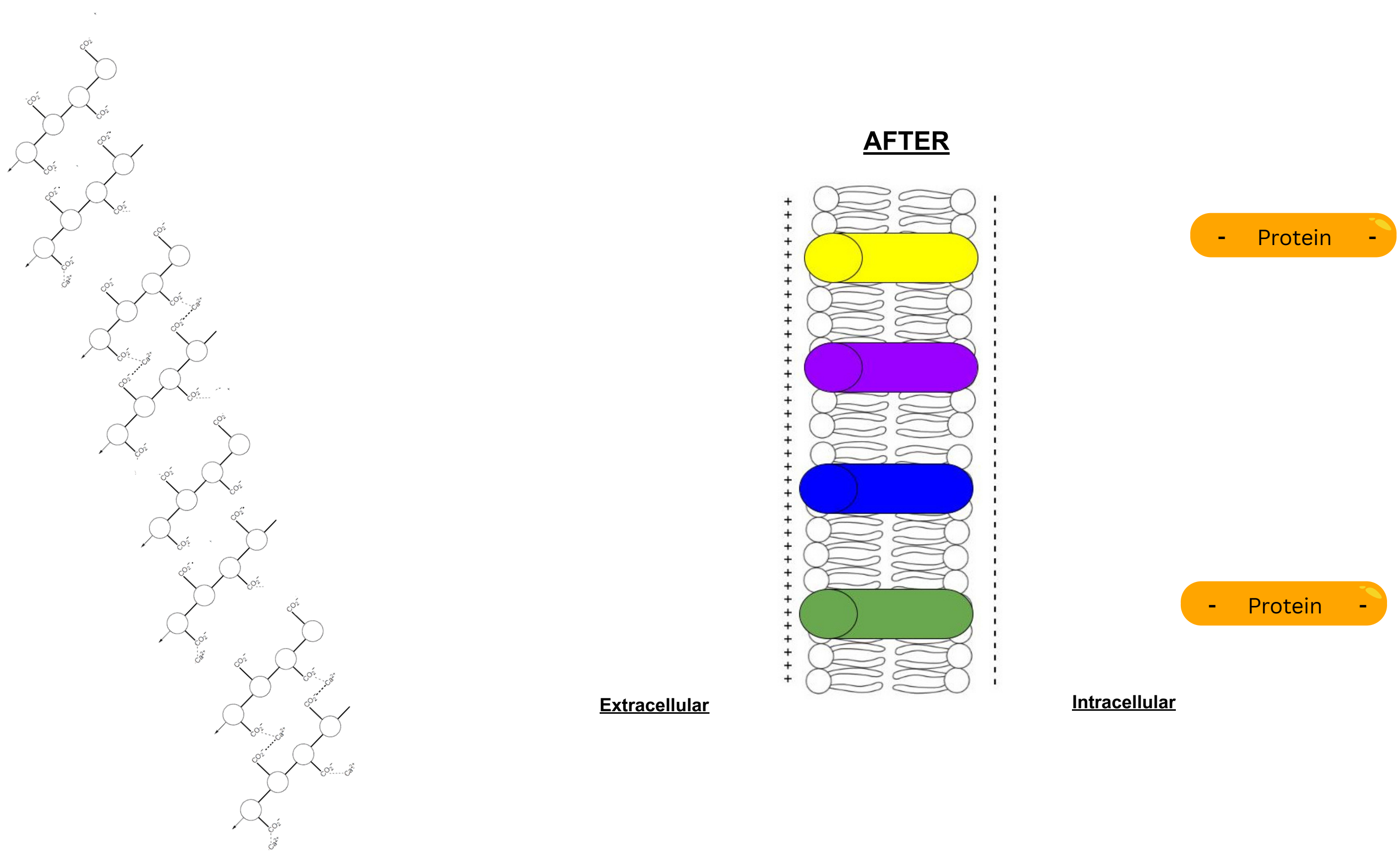

<u>What would happen to the drug delivery hydrogel?</u>

Name ______________________________ Class ____________________

1. What drives simple diffusion across a cell membrane?
A. ATP energy
B. Protein pumps
C. Concentration gradient
D. Enzyme reactions

2. An electrochemical gradient involves:
A. Only differences in pH
B. Only differences in molecule size
C. Chemical and electrical charge differences
D. Concentration of water molecules

3. Why do calcium ions move out of the alginate hydrogel into the external solution?
A. Because the gel pushes them out mechanically
B. Because the external solution is hotter
C. Because of a concentration gradient between the hydrogel and the external solution
D. Because glucose molecules pull them out

Part 2: Short Answer

4. Define Equilibrium

5. What is the difference between **chemical gradient** and **electrochemical gradient**?

6. Why does facilitated diffusion require a transport protein, while simple diffusion does not?

7. What are all the functions of the Cell Membrane?

8. How does the phosphate heads and the lipid/fatty acid tails work together to form a barrier between the inside and outside the cell?

Name ________________________________ Class ____________________

1. What drives simple diffusion across a cell membrane?
A. ATP energy
B. Protein pumps
C. Concentration gradient
D. Enzyme reactions

2. An electrochemical gradient involves:
A. Only differences in pH
B. Only differences in molecule size
C. Chemical and electrical charge differences
D. Concentration of water molecules

3. Why do calcium ions move out of the alginate hydrogel into the external solution?
A. Because the gel pushes them out mechanically
B. Because the external solution is hotter
C. Because of a concentration gradient between the hydrogel and the external solution
D. Because glucose molecules pull them out

Part 2: Short Answer

4. Define Equilibrium- The concentrations inside and outside the cell are Equal/ the Same.

5. What is the difference between **chemical gradient** and **electrochemical gradient**?
With the Chemical Gradient, materials moving from High to Low concentration.
With the Electrochemical Gradient, ions move from High to Low concentration, but can be pulled towards their opposing charge.

6. Why does facilitated diffusion require a transport protein, while simple diffusion does not?
Facilitated Diffusion is the form of transport materials use that are too big to go through the phospholipid bilayer.
With Simple Diffusion materials are small enough to move through the small spaces between the phosphate heads of the phospholipid bilayer.

7. What are all the functions of the Cell Membrane?
-Surrounds the cell
-Creates a barrier between the inside and outside of the cell
-Controls what goes in and out of the cell

8. How does the phosphate heads and the lipid/fatty acid tails work together to form a barrier between the inside and outside the cell?
Water in and outside the cell is attracted to the phosphate heads, but then repelled away from the lipid/fatty acid tails creating a barrier. The water cannot pass through.

Appendix 3

Pre/Post Student Quiz: Full Factorial Experimental Design of Experiments

Pre or Post Assessment: Full Factorial Experimental Design

Scenario

You are designing an experiment to test how two factors-temperature (hot vs cold) and pH (acidic vs neutral) -affect the rate of a chemical reaction. You have access to all necessary materials and will repeat each condition 3 times.

Part A: Multiple Choice & Short Answer (Basic Understanding)

1. What are the two independent variables in this experiment?

A. Temperature and pH
B. Reaction rate and temperature
C. Beaker size and volume
D. Time and concentration

2. How many experimental conditions (treatment combinations) are needed to test a full 2×2 factorial design?

A. 2
B. 4
C. 6
D. 8

3. Why would you replicate each condition multiple times in a factorial design? (Short answer)

____

____

____

____

4. What does it mean to randomize your trials in this context?

A. Use different chemicals
B. Run trials in a random order
C. Change the pH randomly
D. Use random measurement tools

Part B: Conceptual Understanding (Intermediate/Advanced)

5. What is an interaction effect in this experiment? Explain using temperature and pH. (Short answer)

6. Suppose your data shows that at hot temperatures, pH has no effect, but at cold temperatures, acidic pH speeds the reaction. What does this suggest?

A. Only pH matters
B. Only temperature matters
C. There is an interaction between temperature and pH
D. The experiment failed

7. If you only tested one factor at a time, what kind of conclusion would you miss compared to a factorial design? (Short answer)
